# Intrinsically Disordered Segments Affect Protein Half-Life in the Cell and during Evolution

**DOI:** 10.1016/j.celrep.2014.07.055

**Published:** 2014-09-15

**Authors:** Robin van der Lee, Benjamin Lang, Kai Kruse, Jörg Gsponer, Natalia Sánchez de Groot, Martijn A. Huynen, Andreas Matouschek, Monika Fuxreiter, M. Madan Babu

**Affiliations:** 1 MRC Laboratory of Molecular Biology, Francis Crick Avenue, Cambridge CB2 0QH, UK; 2 Centre for Molecular and Biomolecular Informatics, Radboud Institute for Molecular Life Sciences, Radboud University Medical Centre, 6500 HB Nijmegen, the Netherlands; 3 Centre for High-Throughput Biology, University of British Columbia, East Mall, Vancouver BC V6T 1Z4, Canada; 4 Department of Molecular Biosciences and Center for Systems and Synthetic Biology, University of Texas at Austin, Austin, TX 78712, USA; 5 MTA-DE Momentum Laboratory of Protein Dynamics, Department of Biochemistry and Molecular Biology, University of Debrecen, Debrecen 4032, Hungary

## Abstract

Precise control of protein turnover is essential for cellular homeostasis. The ubiquitin-proteasome system is well established as a major regulator of protein degradation, but an understanding of how inherent structural features influence the lifetimes of proteins is lacking. We report that yeast, mouse, and human proteins with terminal or internal intrinsically disordered segments have significantly shorter half-lives than proteins without these features. The lengths of the disordered segments that affect protein half-life are compatible with the structure of the proteasome. Divergence in terminal and internal disordered segments in yeast proteins originating from gene duplication leads to significantly altered half-life. Many paralogs that are affected by such changes participate in signaling, where altered protein half-life will directly impact cellular processes and function. Thus, natural variation in the length and position of disordered segments may affect protein half-life and could serve as an underappreciated source of genetic variation with important phenotypic consequences.

## Highlights

• Genomic principles describing how disordered segments influence protein half-life
• Proteins with terminal or internal disordered segments have a short half-life
• The relationship is evolutionarily conserved between yeast, mouse, and human
• Divergence in disordered segments after gene duplication impacts protein half-life

## Graphical Abstract

**Figure:**
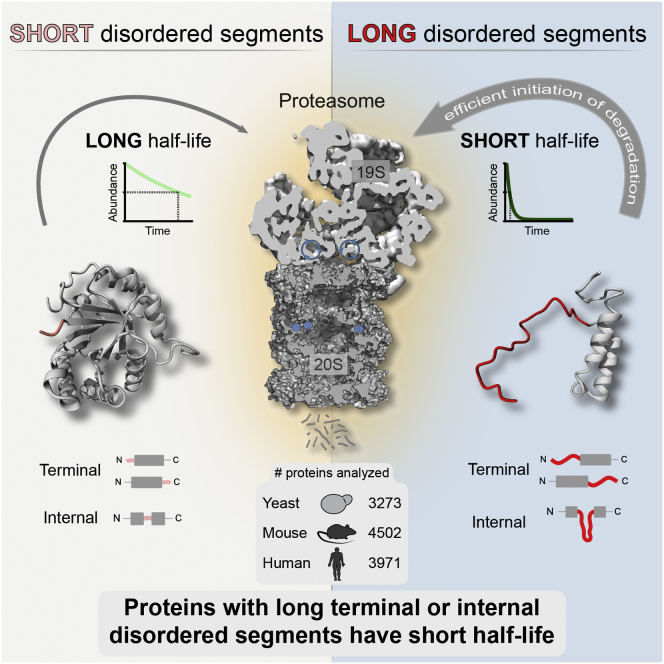

## Teaser

Cellular function requires precise adjustment of protein concentrations. Protein degradation is the endpoint of gene expression and thus a primary determinant of protein abundance. Van der Lee et al. report that the length and number of disordered regions in substrates are two fundamental genetic and evolvable parameters that affect protein half-life and hence protein abundance in cells. They suggest that natural variation in disordered segments of proteins may serve as an underappreciated source of genetic variation with important phenotypic consequences.

## Introduction

Protein degradation is the endpoint of gene expression, and correct turnover of proteins is essential for cellular function. Indeed, protein half-life impacts virtually all cellular processes including the cell cycle (Pagano et al., 1995), DNA repair (Lakin and Jackson, 1999), apoptosis and cell survival (Rutkowski et al., 2006), alternative splicing (Irimia et al., 2012), circadian rhythm (van Ooijen et al., 2011), cell differentiation (Ramakrishna et al., 2011), development (Hirata et al., 2004), and immunity (Babon et al., 2006). Altered protein half-life can lead to abnormal development and diseases such as cancer and neurodegeneration (Ciechanover, 2012). For instance, artificially extending the half-life of the Hes7 transcription factor by ∼8 min severely disorganizes embryonic development in mice (Hirata et al., 2004). Missense mutations in succinate dehydrogenase that increase turnover rates contribute to neuroendocrine tumors (Yang et al., 2012).

The proteasome mediates controlled and selective degradation of most proteins in eukaryotic cells, and access to the proteasome is key to controlling the half-life of substrates (Goldberg, 2003, Hershko and Ciechanover, 1998). Substrate recruitment to the proteasome is primarily mediated through their polyubiquitination by ubiquitin ligases (Komander and Rape, 2012, Ravid and Hochstrasser, 2008, Varshavsky, 2012). This mechanism regulates the half-life of proteins, which ranges from seconds to days (Belle et al., 2006, Kristensen et al., 2013, Schwanhäusser et al., 2011). The large number of ubiquitin ligases and deubiquitinating enzymes encoded in eukaryotic genomes highlights the importance of this system (Hutchins et al., 2013, Komander et al., 2009). Although the role of ubiquitination in delivering proteins to the proteasome is well established, it remains unclear to what extent intrinsic structural features of substrates influence their half-life once bound to the proteasome and whether such features have been exploited to alter half-life during evolution.

An important feature implicated in affecting protein half-life is the presence of polypeptide regions that do not adopt a defined 3D structure, typically called intrinsically disordered, or unstructured regions (van der Lee et al., 2014). Disordered regions are present in a large number of eukaryotic proteins and play key roles in protein function along with structured domains (Babu et al., 2012). A number of genome-scale studies have investigated the relationship between the overall fraction of disordered residues of a protein and its half-life, but these have yielded contradictory results ranging from no correlation (Yen et al., 2008) to weak correlation (Tompa et al., 2008) to a strong effect (Gsponer et al., 2008). The reason for the inconsistencies is perhaps that these studies investigated correlations without the guidance provided by the biochemical mechanism by which disordered regions might contribute to protein turnover.

In this work, we develop a theory of how disordered segments influence protein half-life, through a systematic analysis of multiple data sets describing sequence, structure, expression, evolutionary relationships, and experimental half-life measurements from both unicellular and multicellular organisms. We present evidence that proteins with a long terminal or internal disordered segment have a significantly shorter in vivo half-life in yeast on a genomic scale. The same relationship is found in mouse and human. Upon gene duplication, divergence in terminal and internal disordered segments leads to altered half-life of paralogous proteins. Many affected paralogs participate in signaling pathways, where altered half-life will influence signaling outcomes. We suggest specific biochemical mechanisms by which disordered segments may influence degradation rates, how these changes might modulate cellular function and phenotype, and how natural variation in the length and position of intrinsically disordered protein regions may contribute to the evolution of protein half-life.

## Results

To investigate the relationship between the structural architecture of proteins and their cellular stability, we inferred the disorder status of every residue in the proteomes of yeast, mouse, and human using the DISOPRED2 (Ward et al., 2004), IUPRED (Dosztányi et al., 2005), and PONDR VLS1 (Obradovic et al., 2005) software. In vivo protein half-life data for yeast were obtained from a study that used strains in which proteins expressed from their endogenous promoter contained a tandem affinity purification (TAP) tag at the C terminus (Belle et al., 2006). After inhibition of protein synthesis, protein abundance was measured at three time points by western blotting with TAP antibodies. Protein turnover in mouse and human cells was measured using isotope labeling and mass spectrometry (Kristensen et al., 2013, Schwanhäusser et al., 2011). We combined the information on protein half-life, and other large-scale data sets, with the position and length of disordered segments and analyzed the data using appropriate statistical tests (3,273 proteins in yeast, 4,502 in mouse, and 3,971 in human; Experimental Procedures; Table S1A; Figure S1A).

### Long N-Terminal Disordered Segments Contribute to Short Protein Half-Life In Vivo

**Figure 1:**
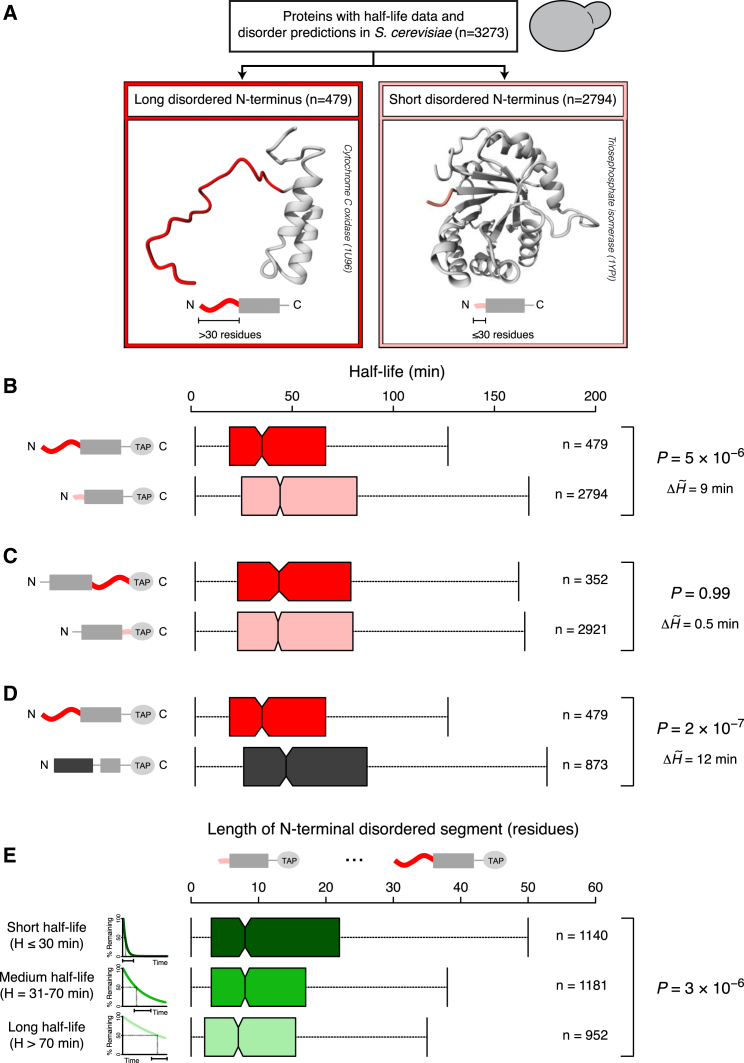
The Effects of Terminal Disordered Segments on Protein Half-Life (A) A total of 3,273 yeast proteins were grouped based on the length of the disordered segment at the N terminus. Long (dark red) and short (light red) terminal disordered segments were defined as stretches of >30 and ≤30 disordered residues. (B–D) Boxplots of protein half-life distributions. Proteins were classified based on the length of the disordered segment at the N terminus (B) or the C terminus (C) and the presence of N-terminal disordered or structured segments (D, long N-terminal structured regions [dark gray] were defined as >30 structured residues). (E) Boxplots of the distributions of N-terminal disorder length for different half-life groups, indicated with schematic exponential degradation curves (from short half-life [dark green] to long half-life [light green]). Central boxplot notches mark the median and the 95% confidence interval. Colored boxes represent the 50% of data points above (×0.75) and below (×0.25) the median (×0.50). Vertical lines (whiskers) connected to the boxes by the horizontal dashed lines represent the largest and the smallest nonoutlier data points. Outliers are not shown to improve visualization. p values reported are from Mann-Whitney *U* (B–D) and Kruskal-Wallis (E) tests. p values, the number of data points (n), and differences between the half-life medians of the compared groups $\left(\Delta \overset{˜}{\text{H}}\right)$ are shown to the right. See also Figures S1 and S3 and Table S1.

We first classified yeast proteins into two groups depending on the length of the disordered termini, treating the N and C termini separately: those with short (≤30 residues) and those with long (>30 residues) disordered tails (Figure 1A). The length cutoff was based on recent molecular models of the proteasome (da Fonseca et al., 2012, Lander et al., 2012, Lasker et al., 2012) and on in vitro biochemical studies using purified proteasomes showing that there is a critical minimum length of ∼30 residues that allows a disordered terminus of a ubiquitinated substrate to efficiently initiate degradation (Inobe et al., 2011). Indeed, analysis of the yeast data confirms that protein half-life does not depend linearly on the length of disordered segments (Figure S1B; Supplemental Experimental Procedures).

Proteins with a long disordered N terminus have a significantly shorter half-life compared to proteins with a short disordered N terminus (p = 5 × 10^−6^, Mann-Whitney *U* test, a nonparametric test for assessing whether two samples come from the same underlying distribution [H_0_]; Figure 1B). The approach for measuring half-lives in yeast involved C-terminal tagging with a TAP tag, which is 186 amino acids long and largely structured. Since all proteins had identical C termini due to the TAP tag, we should see little difference in half-life between proteins with long and short C-terminal disorder as characterized from the original genome sequence. Indeed, these groups display similar distributions of protein half-life (p = 0.99, Mann-Whitney *U* test; Figure 1C).

In order to assess to what extent the disordered state of the N terminus affects half-life, we performed three analyses. First, we investigated proteins with a highly structured N terminus (>30 residues predicted to be structured) and found that they display a longer half-life compared to proteins with a long disordered N terminus (p = 2 × 10^−7^, Mann-Whitney *U* test; Figure 1D). Second, we classified the proteome into three groups of roughly equal size, based on their half-life: (1) short-lived proteins (half-life ≤ 30 min), (2) medium half-life proteins (31–70 min), and (3) long-lived proteins (>70 min) (Figure S1A). The distributions of the length of N-terminal disorder differ significantly across the three groups in a manner consistent with the above observations: proteins with a shorter half-life tend to have longer N-terminal disordered segments (p = 3 × 10^−6^, Kruskal-Wallis test, which extends the Mann-Whitney *U* test to three or more groups, Figures 1E and S1F). Again, this relationship is not true for the C terminus, because the TAP tag causes all proteins to have the same C terminus (p = 0.2, Kruskal-Wallis test; Figures S1E and S1F). Third, we quantified the effects of disordered segments on half-life by comparing conditional probabilities for finding proteins with and without long N-terminal disorder within specific half-life ranges. The likelihood of finding a protein with a short half-life among those that have long N-terminal disorder was two times higher than the “reverse” probability of finding proteins with long N-terminal disorder among those with short half-life (p[short half-life given long N-terminal disorder] = 0.44; p[long N-terminal disorder given short half-life] = 0.18; Tables 1A and S3A). This indicates that the presence of a long disordered N terminus often results in short half-life but proteins with short half-life need not always have a long N-terminal disordered segment. Thus, the presence of a disordered N terminus is linked to short half-life, but other properties also affect protein turnover (see Discussion).

**Table 1:** Conditional Probabilities for Intrinsically Disordered Segments and Protein Half-Life

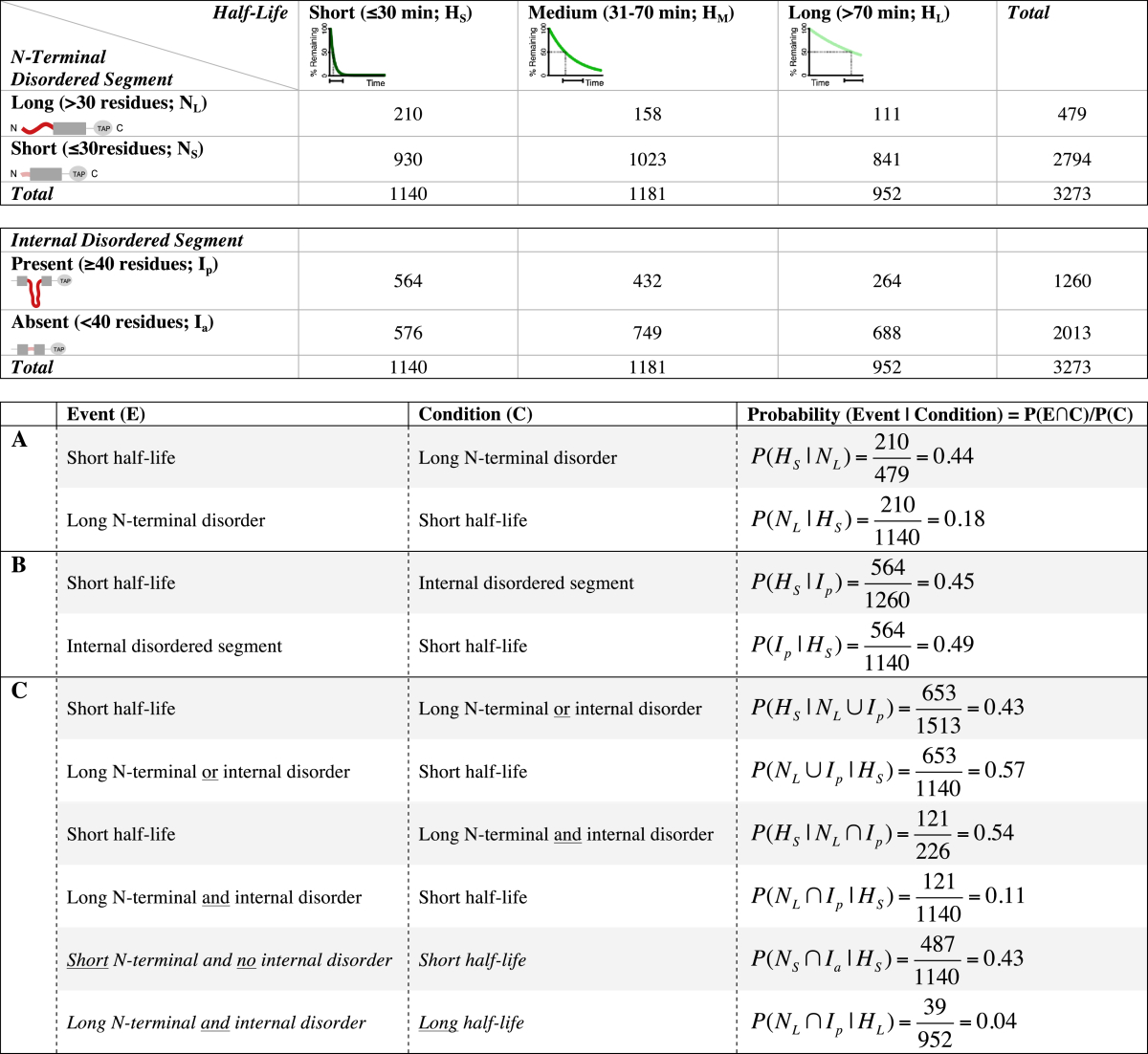

**Figure 2:**
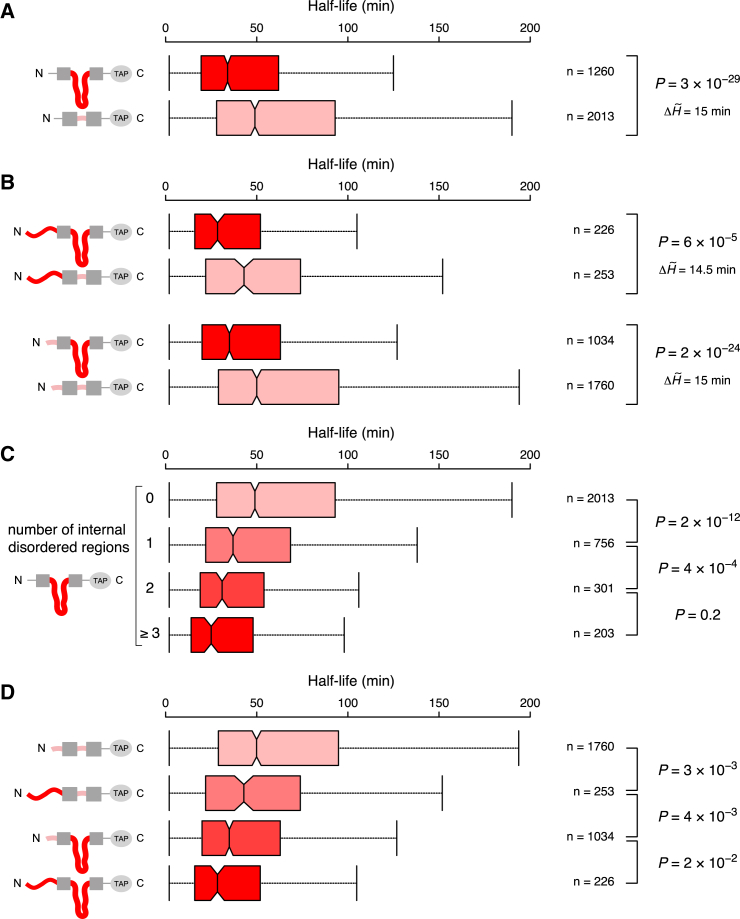
The Effects of Internal Disordered Segments on Protein Half-Life (A) Boxplots of protein half-life distributions for different groups of yeast proteins that contain (dark red) or lack (light red) an internal disordered segment (defined as a continuous stretch of ≥40 disordered residues), subclassified based on (B and D) the length of N-terminal disorder (as in Figure 1: long, >30 residues or short, ≤ 30 residues) and (C) the number of internal disordered segments (from zero, top, to three or more, bottom). Each protein is present in only one category per panel. See Figure 1 for further information. See also Figures S2 and S3 and Tables S1 and S4.

See the top panel of this table and Figures 1 and 2 for a description of the definitions. See also Tables S3A (for part A) and S3B (for part B).

### Internal Disordered Segments Also Contribute to Short Protein Half-Life

The proteasome not only digests proteins starting from their termini but also can cleave or initiate from disordered regions in the middle of the chain (Fishbain et al., 2011, Liu et al., 2003, Piwko and Jentsch, 2006, Prakash et al., 2004, Takeuchi et al., 2007, Zhao et al., 2010). The catalytic residues for proteolysis are buried deep within the proteasome core particle, accessible only through a long narrow channel, and the same is true for the ATPase motor that drives protein substrates through the degradation channel (da Fonseca et al., 2012, Lander et al., 2012, Lasker et al., 2012). To reach these sites, a disordered segment in the middle of a protein has to be longer than a segment at a protein terminus (Fishbain et al., 2011). Therefore, to investigate whether the presence of internal disorder influences protein half-life, we identified proteasome-susceptible internal disordered segments as continuous stretches of at least 40 disordered amino acids (see Discussion). Proteins that contain such an internal disordered segment have a significantly shorter half-life than proteins that do not (p = 3 × 10^−29^, Mann-Whitney *U* test; Figure 2A). This observation is robust to our choice of cutoff used for detecting internal disordered segments, but systematically varying the length cutoff revealed that maximal difference in median half-life is obtained for a value of 40 amino acids (Table S1E). Further, the relationship is independent of N-terminal disorder, as the half-life of proteins with internal disordered segments is significantly lower than of those without, regardless of the length of the disordered terminus (Figure 2B).

To quantify the contribution of internal disorder to protein half-life, we computed conditional probabilities for finding proteins with and without internal disordered segments within specific half-life ranges. The probability of observing a protein with a short half-life among those that contain an internal disordered segment is high and comparable to the “reverse” probability of finding a protein containing an internal disordered segment among those with short half-life (p[short half-life given internal disordered segment] = 0.45; p[internal disordered segment given short half-life] = 0.49; Tables 1B and S3B). This suggests that presence or absence of an internal disordered segment is an important determinant of the half-life of a protein.

### Terminal and Internal Disordered Segments Have Combined Effects on Half-Life

Interestingly, proteins with multiple internal disordered segments have even shorter half-lives than proteins with a single segment (Figures 2C and S2C). This prompted us to investigate the combinatorial effects of terminal and internal disordered segments. Indeed, proteins that have both a long terminal disordered segment and an internal disordered segment tend to have the shortest half-lives (Figure 2D). Furthermore, the probability of having either a terminal or an internal disordered segment given that a protein has a short half-life is the highest (p[long N-terminal or internal disorder given short half-life] = 0.57; Table 1C). Consistent with this observation, we find that the probability of having both terminal and internal disordered segments among proteins with a long half-life is very low (p[long N-terminal and internal disorder given long half-life] = 0.04; Table 1C). Taken together, these results suggest that disordered segments are modular in their ability to affect protein half-life and that these segments can act in a combinatorial manner to accentuate their effects.

### The Effects of Disordered Segments on Half-Life Are Independent of the Overall Disorder Degree

So far, we have investigated the effects of continuous stretches of disordered residues (i.e., disordered segments) on protein turnover. However, the fraction of disordered residues (i.e., overall degree of disorder), which is an estimate of the packing, folding, and structural stability of a protein, also correlates with half-life, although previous studies disagree on the extent of the effect (Gsponer et al., 2008, Tompa et al., 2008, Yen et al., 2008). Proteins with a greater overall disorder degree generally contain longer terminal and internal disordered segments (Figure S3A). To determine whether the effects of disordered segments on protein turnover (Figures 1 and 2) are independent of the overall degree of disorder, we matched proteins that have a similar fraction of disordered residues but have varying combinations of disordered segments (long or short N-terminal disorder and/or presence or absence of internal disordered segments; Supplemental Results; Figure S3B).

Comparison of the half-life distributions of proteins from different classes with similar overall disorder degrees (Figure S3C) reveals similar trends as the analysis that uses all proteins (Figure 2D): proteins with both long N-terminal and internal disordered segments typically have the shortest half-lives, followed by proteins with either long internal or long N-terminal disordered segments. Proteins without disordered segments typically have the longest half-lives. The effect sizes of the differences between the half-life distributions are comparable when using all or only proteins with matched overall disorder degree (Figure S3D, upper triangles). Furthermore, most half-life distributions are significantly different, though p values are less significant due to smaller sample sizes (Figure S3D, lower triangles). These results indicate that long disordered segments at the N terminus or internally are important intrinsic features that contribute to shorter protein half-life in living cells and that these effects are independent of the fraction of disordered residues across the whole protein. It should, however, be noted that this does not rule out an additional effect of the overall disorder degree on half-life, i.e., among proteins that do or do not have a disordered segment, proteins with higher degrees of overall disorder tend to have a lower half-life compared to those with a lower degree of disorder (see Discussion).

### Disordered Segments Have Direct Effects on Half-Life Rather than Acting Indirectly by Embedding Destruction Signals

Disordered segments could influence half-life either indirectly, by embedding short peptide motifs that serve as destruction signals such as ubiquitination sites or docking sites for ubiquitinating enzymes (Ravid and Hochstrasser, 2008), or directly, by better initiating degradation by the proteasome (Gödderz et al., 2011, Inobe et al., 2011, Peña et al., 2009, Piwko and Jentsch, 2006, Prakash et al., 2004, Takeuchi et al., 2007, Verhoef et al., 2009, Zhao et al., 2010). To investigate the indirect effects, we collected data on four known destruction signals: experimentally determined ubiquitination sites as well as predicted KEN box motifs, destruction box motifs, and PEST sequences. More than half (56%) of all proteins with long terminal or internal disordered segments do not contain any of these destruction signals in their disordered segments that could account for the short half-life (Supplemental Results). Consistently, half-life distributions of proteins with and without predicted destruction signals within the disordered regions were not significantly different (p = 0.1 for N-terminal disorder; p = 0.2 for internal disorder; Mann-Whitney *U* test; Supplemental Results). Indeed, the majority of experimentally determined ubiquitination sites involved in degradation are in structured rather than disordered regions (Hagai et al., 2011). Furthermore, sequence analysis revealed that disordered segments of proteins with short half-life lack enriched, uncharacterized sequence motifs that could result in rapid degradation, for example by serving as docking sites for ubiquitin ligases (Supplemental Results). Together, these findings suggest that disordered segments do not affect half-life primarily indirectly by embedding destruction motifs. Rather, the general characteristics of disordered segments seem to directly result in short half-life by forming initiation sites for degradation by the proteasome.

### Disordered Segments Have Similar Effects on Protein Turnover in Mouse and Human

**Figure 3:**
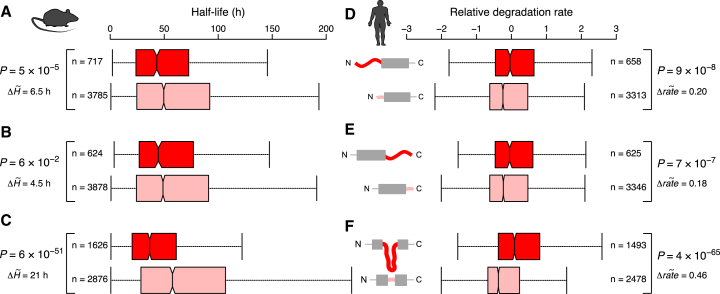
The Effects of Disordered Segments on Protein Turnover in Mouse and Human Boxplots of the distributions of half-life values in *Mus musculus* (A–C), and relative degradation rates in *Homo sapiens* (D–F), for proteins with long and short N-terminal, C-terminal, and internal disordered segments. Note that the scale for protein half-life is in hours for mouse, rather than minutes as in yeast. Values are reversed for the human data: proteins with a short half-life have a high relative degradation rate. See Figure 1 for further information. See also Table S5.

Given that the ubiquitin-proteasome system and the architecture of the proteasome itself are conserved from yeast to mammals (da Fonseca et al., 2012, Lasker et al., 2012), we hypothesized that the observed relationships may be evolutionarily conserved. We investigated the effects of terminal and internal disordered segments on protein degradation in mouse NIH 3T3 fibroblasts (Schwanhäusser et al., 2011) and in human THP-1 myelomonocytic leukemia cells (Kristensen et al., 2013) and found similar trends: the presence of N-terminal and internal disordered segments is linked with significantly faster protein turnover in both mouse (shorter half-lives) and human (higher degradation rates) (Figure 3; Table S5). In the mouse and human studies, protein degradation was monitored using isotope labeling and mass spectrometry, so that proteins did not need to be tagged at the either terminus (in contrast to the yeast study). Therefore, we could assess the contribution of the disordered segment at the C terminus and found that proteins with a long C-terminal disordered segment display increased turnover in mouse and human, though in mouse, the effect seems smaller than for N-terminal disorder and is not statistically significant (Figures 3B and 3E). These results collectively suggest that the effects of disordered regions on protein half-life are evolutionary conserved.

### Divergence in Disordered Segments during Evolution Can Impact Protein Half-Life

**Figure 4:**
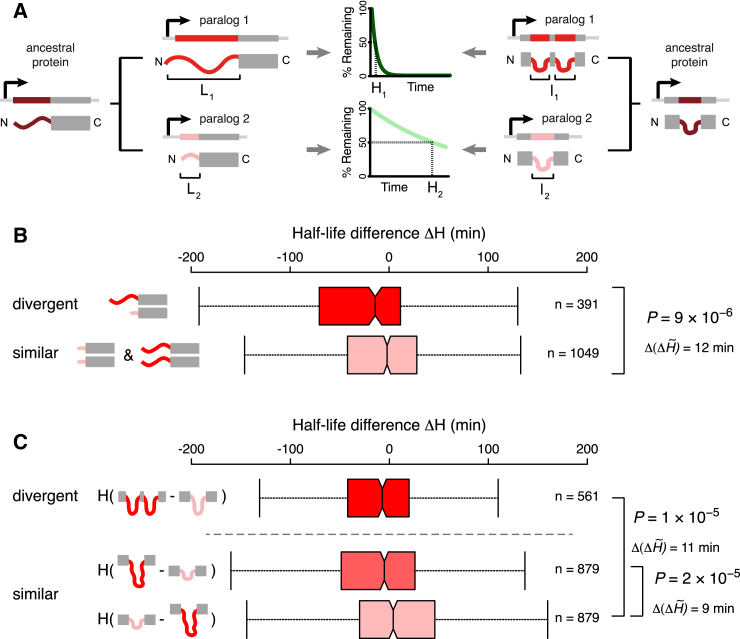
Divergence in Disordered Segments during Evolution Can Impact Protein Half-Life (A) Schematic depiction of how the half-life of paralogs could be altered by changes in N-terminal and/or internal disordered segments during evolution. The dark and light green degradation curves denote a short and long half-life. This schematic is not intended to cover all possible scenarios for divergence of disordered segments between paralogs. (B) Distributions of half-life differences (ΔH) in pairs of yeast paralogs, grouped according to the difference in the length of their N-terminal disordered segments. Top: one paralog has a short and the other paralog a long disordered N terminus (SL). Bottom: both paralogs have short (both ≤30 residues; SS) or both have long (both >30 residues; LL) disordered N termini. (C) Distribution of half-life differences (ΔH) in paralog pairs, grouped according to the difference in the number of internal disordered regions (ΔI). Top: pairs where one of the two paralogs has a higher number of internal disordered segments (ΔI ≥ 1). Bottom: pairs with identical numbers of internal disordered segment (ΔI = 0). Each paralog pair is arranged so that ΔL = L_1_ − L_2_ (B) and ΔI = I_1_ − I_2_ (C) are always positive (i.e., L_1_ ≥ L_2_ and I_1_ ≥ I_2_). This order is used for the ΔH calculation (that is, the half-life of the paralog with the shortest N-terminal disorder, or the smallest number of internal disordered segments, will be subtracted from the half-life of the other one; ΔH = H_1_ − H_2_). As a result, ΔH will be negative for pairs where an increase in N-terminal or internal disorder coincides with a shorter half-life (Experimental Procedures). For ΔI = 0 (C, bottom), two ΔH distributions were obtained by ordering the paralogs within a pair according to the total length of all internal disordered segments (increasing and decreasing to simulate gain and loss of internal disorder length during evolution; Table S6A). See Figure 1 for further information. See also Figure S4 and Table S6.

Our observations suggest that protein turnover rates could be tuned by divergence in terminal or internal disordered segments during evolution (Figure 4A). To test this, we investigated protein pairs in yeast that arose from gene duplication (i.e., paralogs) (Experimental Procedures). Since paralogs are encoded within the same genome, this makes it possible to compare half-lives between evolutionarily related proteins under similar conditions. We specifically asked whether paralogs diverged in the length of N-terminal disorder or in the number of internal disordered segments (but are otherwise largely similar) and, if they did, whether this corresponded to changes in their half-life. Protein half-life data are available for both paralogs of 1,440 pairs (Table S7), and many of these paralog pairs have diverged in the length and number of terminal and internal disordered segments (Figures 4B and 4C, Tables S7 and S8).

We classified the pairs of paralogs into (1) those that during evolution maintained N-terminal disorder of roughly equal length (i.e., both proteins have a short [≤30 residues] or both have a long [>30 residues] disordered segment at the N terminus; 1,049 pairs) and (2) pairs with disordered N termini of different length (i.e., one protein of the pair has a short and the other has a long disordered segment; 391 pairs). Paralogous protein pairs that diverged in the length of N-terminal disorder show significantly larger differences in half-lives than pairs that maintained roughly equal N-terminal disorder (p = 9 × 10^−6^, Mann-Whitney *U* test; Figure 4B), in a manner that agrees with the trends reported above: the protein with the longer N-terminal disordered segment usually has a shorter half-life than its paralog with a shorter disordered segment. More precisely, (1) paralogous proteins with similar length of terminal disorder tend to have similar half-life values (median difference in half-life is close to zero; Figure 4B, bottom boxplot; Table S6A), and (2) the half-life of proteins with longer N-terminal disordered regions tends to be 14 min shorter (median) than that of their paralogous partners (Figure 4B, top boxplot; Table S6A). The converse is also true, as paralogous pairs with large half-life changes show a large divergence in the length of N-terminal disorder (Supplemental Results; Figure S4A). A 14 min difference in half-life between paralogs is substantial in the context of yeast biology, as this is comparable to the time from division to budding (G1 phase) in laboratory strains growing exponentially in rich media at 30°C, which is 15–37 min (Di Talia et al., 2007). Thus, altered half-life due to divergence in the length of terminal disorder could have a significant impact on the duration for which a protein can impart its function in a cell and thus affect cellular behavior.

Paralogous proteins that differ in the number of internal disordered segments also show significantly larger changes in half-life than pairs with the same number of internal disordered regions (p = 1 × 10^−5^, Mann-Whitney *U* test; Figure 4C). The half-life of proteins with more internal disordered regions tends to be 7 min shorter (median) than that of their paralogs (Table S6A), which again can be a considerable amount of time considering the doubling time of yeast. In paralogous pairs that have the same number of internal disordered segments but diverged in the total internal disorder length (i.e., the sum of all internal disordered segments), the half-life of the protein with the longer total internal disorder also tends to be shorter (median half-life difference is 5 min; Table S6A).

Analysis of conditional probability values allowed us to quantify the trends (Tables 2 and S8). The majority (73%) of paralogous pairs that diverged in the length of terminal or number of internal disordered segments show a consistent change in half-life: the paralog with the longest terminal disordered segment or largest number of internal disordered segments tends to have the shorter half-life (p[shorter half-life given divergence of N-terminal or internal disorder] = 0.73; Table 2C). This effect is large even if only segments at the N terminus or only internal segments are considered (p [shorter half-life given divergence of N-terminal disorder] = 0.64 and p[shorter half-life given divergence of internal disorder] = 0.58; Tables 2A and 2B). Again, the converse is also true as the probability of observing a paralogous pair that has diverged in both terminal and internal disorder, and in which the paralog with the longest terminal disordered segment and most internal disordered segments has the longer half-life, is very small (p[divergence of N-terminal and internal disorder given longer half-life] = 0.01; Table 2C). Taken together, the results suggest that the gain or loss of long terminal or internal disordered segments can significantly influence the half-life of a protein upon gene duplication during evolution. Thus evolution of intrinsic features such as disordered segments may be an important contributor to the degradation rate of proteins.

**Table 2:** Conditional Probabilities for Intrinsically Disordered Segments and Protein Half-Life in Pairs of Paralogs

	Event (E)	Condition (C)	Probability (Event | Condition) = P(E∩C)/P(C)
A	Shorter half-life	divergence of N-terminal disorder	$P\left(\Delta H<0\min|N_{SL}\right)=\frac{252}{391}=0.64$
	Divergence of N-terminal disorder	shorter half-life	$P\left(N_{SL}|\Delta H<0\min\right)=\frac{252}{787}=0.32$
B	Shorter half-life	divergence of internal disordered segments	$P\left(\Delta H<0\min|I_{n\to n+x}\right)=\frac{323}{561}=0.58$
	Divergence of internal disordered segments	shorter half-life	$P\left(I_{n\to n+x}|\Delta H<0\min\right)=\frac{323}{799}=0.40$
C	Shorter half-life	divergence of N-terminal or internal disordered segments	$P\left(\Delta H<0\min|N_{SL}\cup I_{n\to n+x}\right)=\frac{542}{741}=0.73$
	Divergence of N-terminal or internal disordered segments	shorter half-life	$P\left(N_{SL}\cup I_{n\to n+x}|\Delta H<0\min\right)=\frac{542}{1176}=0.46$
	Shorter half-life	divergence of N-terminal and internal disordered segments	$P\left(\Delta H<0\min|N_{SL}\cap I_{n\to n+x}\right)=\frac{33}{211}=0.16$
	Divergence of N-terminal and internal disordered segments	shorter half-life	$P\left(N_{SL}\cap I_{n\to n+x}|\Delta H<0\min\right)=\frac{33}{1176}=0.03$
	Divergence of N-terminal and internal disordered segments	longer half-life	$P\left(N_{SL}\cap I_{n\to n+x}|\Delta H>0\min\right)=\frac{11}{1013}=0.01$

See Figure 4 for a description of the definitions. N_SL_ denotes pairs where one paralog has a short and the other paralog a long N-terminal disordered segment. I_n = > n+x_ denotes pairs where one of the two paralogs has a higher number of internal disordered segments (ΔI ≥ 1). See also Tables S8A (for part A) and S8B (for part B).

### Functional Analysis and Literature Evidence Support the Role of Disordered Segments in Governing Protein Half-Life and Phenotype

Disordered segments that affect half-life could be important for governing phenotypes, because precise protein turnover is important for many cellular processes. An analysis of function annotations of proteins with long N-terminal or internal disordered segments revealed enrichment for protein kinases and phosphoproteins and associations with regulatory and transcription functions, as well as cell-cycle processes (Tables S1F and S4B). Paralogs that have diverged in terminal or internal disordered segments have similar functions and are additionally involved in, for example, ATP and nucleotide binding and ubiquitin conjugation activities (Table S6C). These are all functions involved in signaling and regulation, where alteration of protein half-life can significantly affect the duration of activity of the protein and thereby impact cellular phenotype (Legewie et al., 2008) (see Discussion).

A literature search revealed several examples where changes in disordered segments lead to phenotypic differences through altered protein half-life. Stabilizing the half-life of the yeast kinase Ime2, a positive regulator of meiosis, by deletion of an internal disordered region results in altered sporulation efficiency (Guttmann-Raviv et al., 2002). Similarly, deletion of a highly disordered 47-amino-acid stretch at the N terminus of yeast Cdc6 prevents its degradation, although in this case, the deletion also abolishes the interaction with a ubiquitin ligase complex (Drury et al., 1997). Deletion of the first 31 residues of the human nuclear receptor Nurr1 significantly reduces its degradation by the ubiquitin-proteasome pathway and consequently leads to increased activation as a transcription factor (Alvarez-Castelao et al., 2013). Interestingly, the deleted region corresponds completely to a putative disordered segment at the N terminus and the size of the deletion could now explain the effects on half-life. These selected examples illustrate the importance of disordered segments for maintaining correct protein turnover.

## Discussion

**Figure 5:**
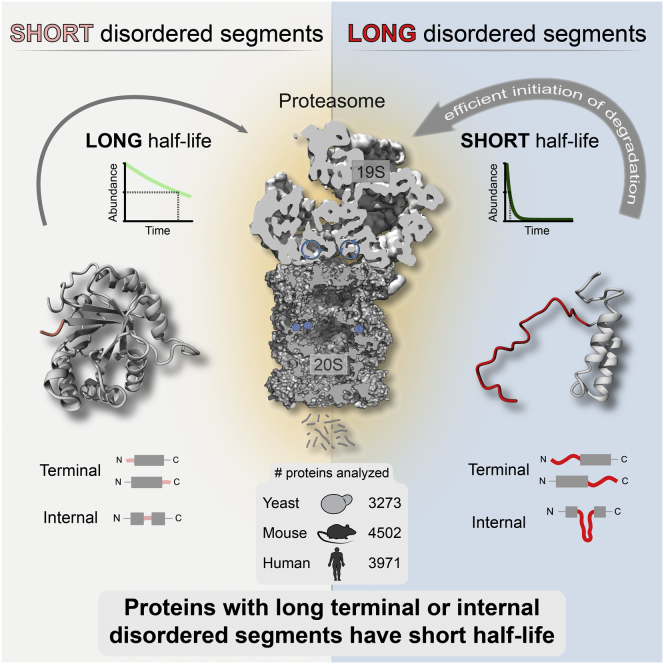
Concept Describing the General Relationship between the Presence of Long Terminal or Internal Disordered Segments and Protein Half-Life Disordered segments influence protein half-life by permitting efficient initiation of degradation by the proteasome. Ubiquitination and other factors contributing to substrate targeting to the proteasome are not depicted.

Ubiquitination by E3 ligases has a dominant role in deciding when a protein gets targeted for proteasomal degradation, but it has remained unclear how intrinsic features affect the lifetime of a protein and whether such features have been exploited to alter half-life during evolution. Here, we uncovered genome-scale principles of how intrinsically disordered segments influence protein turnover in the cell and during evolution. On a genomic scale, in vivo, sufficiently long disordered regions at the termini or in the middle of proteins can directly decrease half-life (Figure 5). A large number of control calculations confirmed that the reported trends are independent of confounding factors, such as the cutoffs used to group the proteins, the disorder prediction method, the statistical tests used, protein abundance and length, subcellular localization, membrane proteins, and the nature of the N-terminal residue (Supplemental Results). Finally, we found that changes in the length and number of disordered segments upon gene duplication are linked with altered half-life, suggesting that such variation can contribute to the tuning of half-life during evolution.

### The Structure and Composition of the Proteasome Suggest Molecular Mechanisms to Explain the Observations

The structure of the 19S regulatory particle (da Fonseca et al., 2012, Lander et al., 2012, Lasker et al., 2012) provides insights into the mechanisms by which disordered segments may increase the efficiency of proteasomal degradation and affect protein half-life. The distance between the two ubiquitin receptors, Rpn10 and Rpn13, and the ATPase unfolding channel is ∼70–80 Å. The essential deubiquitinating enzyme Rpn11 sits ∼60 Å from the ATPase ring. A terminal disordered segment of 30 residues would comfortably span these distances and could serve as a degradation initiation site. Similarly, 40 residues would be enough for an internal disordered segment to reach into the ATPase ring of the regulatory particle, even when folding back on itself. The precise distance requirements for a disordered segment to serve as an initiation site will depend on specific properties of the proteasome and the geometry and binding position of the substrate. For example, at least five different substrate receptors associate with the yeast proteasome, and some of them exhibit extensive conformational flexibility (Finley, 2009). Substrate-specific aspects that affect the distances include the state of the termini, which are frequently subject to maturation through cleavage and trimming (Lange and Overall, 2013), and properties of the polyubiquitin tag such as the linkage type (e.g., K48 and K11) and number of ubiquitin moieties (Komander and Rape, 2012), and the attachment point to the substrate (Hagai et al., 2011, Inobe et al., 2011). This could explain why, with in vivo data for thousands of different proteins, we do not observe a strict length cutoff for when disordered segments influence protein half-life: cutoffs of about 30 terminal and 40 internal disordered residues produce the largest differences between the half-lives of proteins with and without disordered segments, but shorter and longer segments also contribute to shorter half-life (Table S1E). Thus, individual proteins are likely to have specific length requirements of disordered segments that depend on a variety of factors and contribute to the range of lengths at which disordered segments decrease protein half-life on a global scale.

The ATP-independent regulators PA28 and P200 can also facilitate opening of the 20S proteasome entry gate and contribute to substrate degradation (Stadtmueller and Hill, 2011). The ATPase complex p97/VCP perhaps serves as an alternative cap that directly binds the 20S core particle as well (Barthelme and Sauer, 2012). All these complexes may have different requirements for disordered segments in the substrate proteins. In fact, it has been suggested that p97/VCP may unfold proteins lacking disordered regions (Beskow et al., 2009). In vitro, the 20S proteasome core particle by itself can degrade highly disordered proteins in a process termed degradation by default and it may also be able to do so in vivo (Tsvetkov et al., 2008). The average distance between the entry pore and the proteolytic sites in the 20S core particle is ∼70 Å (da Fonseca et al., 2012, Lasker et al., 2012). An internal disordered segment of at least 40 residues is able to span twice this distance and thus could be cleaved by the core particle alone (Figure S2D). Thus, proteins with disordered segments of specific length may be processed quickly due to efficient initiation of degradation as discussed.

The overall disorder degree of a substrate might further affect its half-life. Upon initiation of degradation, the proteasome may quickly degrade proteins with high overall levels of disorder, because its ATPase subunits spend less time to unfold these disordered proteins once they are engaged compared to proteins of similar length that are structured and need to be unfolded before they can be processed. Indeed, biochemical evidence suggests increasingly structured and stable substrates have higher turnover times and energy costs (Henderson et al., 2011, Peth et al., 2013).

### Disordered Segments Influence Half-Life as an Intrinsic Feature that Can Be Modulated by Other Mechanisms

Although proteins with long terminal or internal disordered segments tend to have a short half-life, various factors can increase or decrease the half-life of individual proteins (see also Supplemental Discussion). For example, the presence of a highly structured N-terminal domain may shield proteins with internal disordered segments from degradation (Simister et al., 2011). Disordered proteins may also be protected by forming protein complexes or through interactions with other proteins. For instance, several specialized proteins have been shown to bind to and stabilize disordered proteins (Tsvetkov et al., 2009). Furthermore, specific low-complexity sequences or tandem repeats in the degradation initiation site can attenuate initiation or progression of degradation, thereby affecting substrate half-life (Sharipo et al., 1998, Tian et al., 2005, Zhang and Coffino, 2004). This is consistent with the idea that, although disordered segments are all similar in that they lack the ability to independently fold into a compact structure, many types of sequences exist within this definition that have different biophysical and conformational characteristics. For example, some disordered sequences are relatively globular and collapsed while others are expanded, and this is determined by the overall charge and sequence composition of the disordered region (Mao et al., 2013). Indeed, the proteasome has clear amino acid sequence preferences as degradation of model substrates that differ only in their disordered initiation regions varies over at least an order of magnitude (Fishbain et al., 2014). The broad distributions of half-lives observed in our study support this, as they reflect the combined properties of many possible subtypes of disordered segments, some of which are able to efficiently initiate degradation, while others may not.

In short, one can distinguish at least three distinct determinants of protein half-life. (1) Sequence motifs and the presence of regulatory proteins such as ubiquitin ligases contribute to the overall half-life of a protein by determining when substrates will be ubiquitinated and hence targeted to the proteasome. (2) Upon recognition of the ubiquitinated substrate by the proteasome, the presence of disordered segments of sufficient size either at the terminus or internally may facilitate efficient degradation initiation, thereby leading to lower half-life. (3) The overall degree of disorder may contribute to a general trend in lowering half-life by increasing the processivity of degradation upon engagement (after recognition and initiation) by the proteasome. Thus, our observations suggest that disordered segments influence protein half-life as an underlying factor that can be modulated by other cellular mechanisms, sequence determinants and the structural stability of the substrate.

### Disordered Segments Could Influence the Dynamics and Regulation of Signaling Pathways

Disordered regions are prominent in regulatory and signaling proteins (Tables S1F, S4B, and S6C) (van der Lee et al., 2014). Since divergence in disordered segments may affect protein half-life, this could influence the response kinetics of signaling and regulatory pathways involving such proteins (see also Supplemental Discussion). In fact, among the paralogous pairs that show the largest divergence in the length of N-terminal disorder and half-life in yeast (Tables S6C and S7), there are several regulatory protein kinases such as MAP kinases (*MKK1*, *MKK2*, *HOG1*, and *STE7*), serine/threonine kinases (*YPK1*, *YPK2*, and *KIN28*), and cyclin-dependent kinases (*PHO85* and *CAK1*). Paralogs that have diverged in terminal or internal disordered segments are generally enriched in nucleotide binding, kinase regulatory activity, and phosphoproteins. Alterations in the degradation rate of kinases, for instance, can have significant implications for the dynamics of signaling networks (Legewie et al., 2008, Purvis and Lahav, 2013). Such effects have been shown for the yeast kinase Ime2 (Guttmann-Raviv et al., 2002) and mouse transcription factor Hes7 (Hirata et al., 2004), where mutations in disordered segments lead to changes in protein half-life, which in turn severely deregulate signaling and development, respectively.

Our observations raise the possibility that proteins with a long terminal disordered segment might be better presented as antigens to the immune system. This is because the immunoproteasome, a variant of the canonical proteasome, may better process such proteins into peptides for presentation by the major histocompatibility complex (MHC) molecules (Groettrup et al., 2010). In line with this idea, it has been shown that (1) N-extended epitopes are efficiently processed by the immunoproteasome and serve as better substrates for antigen presentation (Cascio et al., 2001) and (2) the presence of a disordered region determines the direction of degradation, which in turn determines the spectrum of generated peptides (Berko et al., 2012). It is tempting to speculate that one could improve vaccine efficiency by adding or extending terminal disordered regions to epitope-containing proteins. Our findings also call for careful interpretation of half-life measurements made on proteins that are tagged at their termini using constructs with a varying degree of structure or intrinsic disorder (e.g., GFP, TAP tag, His-tag).

### Divergence in Disordered Segments Provides a Means for Tuning Protein Half-Life during Evolution and Could Generate Phenotypic Variation

We observed that divergence in disordered regions might influence protein half-life among paralogs. An outstanding question is whether such changes in half-life through divergence of disordered segments are under selective pressure. Natural variation leading to alteration of disordered regions may provide a simple means for regulatory subfunctionalization of paralogous proteins upon gene duplication. It also suggests a mechanism for divergence of half-life among orthologous proteins between species. Thus, while it is clear that the emergence of destruction signals such as ubiquitination sites and dedicated ubiquitin ligases affect targeting of a protein for degradation, variation in disordered segments may provide a simple evolutionary mechanism for fine-tuning protein turnover rates.

Several genetic and molecular mechanisms may generate diversity in terminal or internal disordered segments. These include repeat expansion, alternative splicing, and alternative transcription start sites, all of which can influence the length of terminal and/or internal disorder of protein products, thereby potentially influencing the half-life. This idea is supported by the observation that protein disorder is common in insertions and deletions (Light et al., 2013). Furthermore, given that in multicellular eukaryotes (1) alternative transcription start sites commonly generate variation in N termini (Carninci et al., 2006) and (2) alternatively spliced exons are enriched in intrinsic disorder (Buljan et al., 2013), it is likely that such events that generate diversity in protein sequences in different cell types within an individual will have an effect on protein half-life. Similarly, given that disordered regions often contain homopolymeric repeat sequences (Tompa, 2003), and because tandem repeats in DNA sequences can lead to expansion or deletion of genetic material through strand slippage during replication (Levinson and Gutman, 1987), it is plausible that individuals in a population harbor genetic variants that code for proteins with altered length of disordered segments and thus have different half-lives. Changes in protein turnover in turn may disturb protein abundance and could lead to disease (Babu et al., 2011, Hirata et al., 2004, Yang et al., 2012), especially in the case of pleiotropic, regulatory, or signaling proteins. Thus mechanisms that generate diversity in the length or number of disordered segments could serve as a source of genetic variation that may have important phenotypic consequences.

## Experimental Procedures

### Protein Half-Life Data and Calculation of Disordered Segments

Protein half-life data and other data (Table S1A) were collected for yeast (*Saccharomyces cerevisiae*), mouse, and human. Intrinsic disorder was predicted for all reviewed protein sequences of these organisms (downloaded from UniProtKB/Swiss-Prot; http://www.uniprot.org/) using three complementary methods: DISOPRED2, IUPRED long, and PONDR VLS1. The presence and length of N-terminal, C-terminal, and internal disordered segments were then calculated using different algorithms and integrated with the half-life data. Proteomes were classified into groups according to the length of disordered segments: (1) proteins with short and long disordered termini (length cutoff 30 residues, treating the N and C termini separately) and (2) proteins with and without internal disordered segments (at least 40 disordered residues). The overall degree of disorder of a protein was calculated as the fraction of disordered residues (number of disordered residues divided by sequence length). The distributions of half-life values and protein disorder were analyzed using appropriate statistical tests.

See the Supplemental Experimental Procedures for more details.

### Paralog Data and Calculations

Yeast paralog pairs were obtained from an all-against-all sequence comparison using BLASTClust (Altschul et al., 1990). More divergent paralogs from the yeast whole-genome duplication event (Wolfe and Shields, 1997) were added to the list. To calculate the differences in half-life (ΔH) and N-terminal disorder length (ΔL) between the individual proteins in a paralog pair, ΔL is defined to be always positive and obtained by subtracting the N-terminal disorder length of paralog 2 from the N-terminal disorder length of paralog 1 (ΔL = L1 − L2; L1 ≥ L2). To calculate ΔH, the order of paralogs in a pair is maintained, so that ΔH can be positive or negative (ΔH = H1 − H2). Thus, ΔH is negative whenever the relationship “longer disordered N terminus = shorter half-life” holds true. Similarly, the difference in the number of internal disordered segments ΔI is defined to always be positive (ΔI = I1 − I2; I1 ≥ I2), and ΔH is calculated accordingly. Paralog pairs were separated into categories according to the divergence in N-terminal (pairs that maintained or that diverged N-terminal disorder) or internal disordered segments (pairs with an identical or different number of segments).

See the Supplemental Experimental Procedures for more details.

## Author Contributions

R.v.d.L. and M.M.B. designed the project and analyzed and interpreted the results. R.v.d.L. performed the majority of the calculations. B.L. performed additional calculations, and K.K., J.G., N.S.d.G., and M.F. carried out specific analyses. B.L., K.K., M.A.H., M.F., and A.M. assisted in interpretation. R.v.d.L. and M.M.B. wrote the manuscript with help from B.L., M.A.H., M.F., and A.M.

## Supplemental Information

Document S1. Supplemental Results, Supplemental Discussion, Supplemental Experimental Procedures, Figures S1–S4, and Tables S1, S3–S6, and S8

Table S2. The Primary Data Used in This Study

Table S7. Data on N-Terminal Disordered Regions, Internal Disordered Regions, and Half-Life for Paralog Pairs

Document S2. Article plus Supplemental Information
