# Supplementary material for: Intrinsically Disordered Segments Affect Protein Half-Life in the Cell and during Evolution
Source: Cell Rep. 2014 Sep 15;8(6):1832–44. doi: 10.1016/j.celrep.2014.07.055 (PMC4358326; doi:10.1016/j.celrep.2014.07.055)
Supplement: Document S2. Article plus Supplemental Information [file mmc4.pdf]

OPEN ACCESS
CellPress

Cell Reports
Article

Intrinsically Disordered Segments Affect Protein Half-Life in the Cell and during Evolution

Robin van der Lee,[1,2,*] Benjamin Lang,[1] Kai Kruse,[1] Jörg Gsponer,[3] Natalia Sánchez de Groot,[1] Martijn A. Huynen,[2] Andreas Matouschek,[4] Monika Fuxreiter,[5] and M. Madan Babu[1,*]
[1]MRC Laboratory of Molecular Biology, Francis Crick Avenue, Cambridge CB2 0QH, UK
[2]Centre for Molecular and Biomolecular Informatics, Radboud Institute for Molecular Life Sciences, Radboud University Medical Centre, 6500 HB Nijmegen, the Netherlands
[3]Centre for High-Throughput Biology, University of British Columbia, East Mall, Vancouver BC V6T 1Z4, Canada
[4]Department of Molecular Biosciences and Center for Systems and Synthetic Biology, University of Texas at Austin, Austin, TX 78712, USA
[5]MTA-DE Momentum Laboratory of Protein Dynamics, Department of Biochemistry and Molecular Biology, University of Debrecen, Debrecen 4032, Hungary
*Correspondence: rvdlee@mrc-lmb.cam.ac.uk (R.v.d.L.), madanm@mrc-lmb.cam.ac.uk (M.M.B.)
http://dx.doi.org/10.1016/j.celrep.2014.07.055
This is an open access article under the CC BY license (http://creativecommons.org/licenses/by/3.0/).

SUMMARY

Precise control of protein turnover is essential for cellular homeostasis. The ubiquitin-proteasome system is well established as a major regulator of protein degradation, but an understanding of how inherent structural features influence the lifetimes of proteins is lacking. We report that yeast, mouse, and human proteins with terminal or internal intrinsically disordered segments have significantly shorter half-lives than proteins without these features. The lengths of the disordered segments that affect protein half-life are compatible with the structure of the proteasome. Divergence in terminal and internal disordered segments in yeast proteins originating from gene duplication leads to significantly altered half-life. Many paralogs that are affected by such changes participate in signaling, where altered protein half-life will directly impact cellular processes and function. Thus, natural variation in the length and position of disordered segments may affect protein half-life and could serve as an underappreciated source of genetic variation with important phenotypic consequences.

INTRODUCTION

Protein degradation is the endpoint of gene expression, and correct turnover of proteins is essential for cellular function. Indeed, protein half-life impacts virtually all cellular processes including the cell cycle (Pagano et al., 1995), DNA repair (Lakin and Jackson, 1999), apoptosis and cell survival (Rutkowski et al., 2006), alternative splicing (Irimia et al., 2012), circadian rhythm (van Ooijen et al., 2011), cell differentiation (Ramakrishna et al., 2011), development (Hirata et al., 2004), and immunity (Babon et al., 2006). Altered protein half-life can lead to abnormal development and diseases such as cancer and neurodegeneration (Ciechanover, 2012). For instance, artificially extending the half-life of the Hes7 transcription factor by ~8 min severely disorganizes embryonic development in mice (Hirata et al., 2004). Missense mutations in succinate dehydrogenase that increase turnover rates contribute to neuroendocrine tumors (Yang et al., 2012).

The proteasome mediates controlled and selective degradation of most proteins in eukaryotic cells, and access to the proteasome is key to controlling the half-life of substrates (Goldberg, 2003; Hershko and Ciechanover, 1998). Substrate recruitment to the proteasome is primarily mediated through their polyubiquitination by ubiquitin ligases (Komander and Rape, 2012; Ravid and Hochstrasser, 2008; Varshavsky, 2012). This mechanism regulates the half-life of proteins, which ranges from seconds to days (Belle et al., 2006; Kristensen et al., 2013; Schwanhäusser et al., 2011). The large number of ubiquitin ligases and deubiquitinating enzymes encoded in eukaryotic genomes highlights the importance of this system (Hutchins et al., 2013; Komander et al., 2009). Although the role of ubiquitination in delivering proteins to the proteasome is well established, it remains unclear to what extent intrinsic structural features of substrates influence their half-life once bound to the proteasome and whether such features have been exploited to alter half-life during evolution.

An important feature implicated in affecting protein half-life is the presence of polypeptide regions that do not adopt a defined 3D structure, typically called intrinsically disordered, or unstructured regions (van der Lee et al., 2014). Disordered regions are present in a large number of eukaryotic proteins and play key roles in protein function along with structured domains (Babu et al., 2012). A number of genome-scale studies have investigated the relationship between the overall fraction of disordered residues of a protein and its half-life, but these have yielded contradictory results ranging from no correlation (Yen et al., 2008) to weak correlation (Tompa et al., 2008) to a strong effect (Gsponer et al., 2008). The reason for the inconsistencies is perhaps that these studies investigated correlations without the guidance provided by the biochemical mechanism by which disordered regions might contribute to protein turnover.

In this work, we develop a theory of how disordered segments influence protein half-life, through a systematic analysis of

multiple data sets describing sequence, structure, expression, evolutionary relationships, and experimental half-life measurements from both unicellular and multicellular organisms. We present evidence that proteins with a long terminal or internal disordered segment have a significantly shorter in vivo half-life in yeast on a genomic scale. The same relationship is found in mouse and human. Upon gene duplication, divergence in terminal and internal disordered segments leads to altered half-life of paralogous proteins. Many affected paralogs participate in signaling pathways, where altered half-life will influence signaling outcomes. We suggest specific biochemical mechanisms by which disordered segments may influence degradation rates, how these changes might modulate cellular function and phenotype, and how natural variation in the length and position of intrinsically disordered protein regions may contribute to the evolution of protein half-life.

RESULTS

To investigate the relationship between the structural architecture of proteins and their cellular stability, we inferred the disorder status of every residue in the proteomes of yeast, mouse, and human using the DISOPRED2 (Ward et al., 2004), IUPRED (Dosztányi et al., 2005), and PONDR VLS1 (Obradovic et al., 2005) software. In vivo protein half-life data for yeast were obtained from a study that used strains in which proteins expressed from their endogenous promoter contained a tandem affinity purification (TAP) tag at the C terminus (Belle et al., 2006). After inhibition of protein synthesis, protein abundance was measured at three time points by western blotting with TAP antibodies. Protein turnover in mouse and human cells was measured using isotope labeling and mass spectrometry (Kristensen et al., 2013; Schwanhäusser et al., 2011). We combined the information on protein half-life, and other large-scale data sets, with the position and length of disordered segments and analyzed the data using appropriate statistical tests (3,273 proteins in yeast, 4,502 in mouse, and 3,971 in human; Experimental Procedures; Table S1A; Figure S1A).

Long N-Terminal Disordered Segments Contribute to Short Protein Half-Life In Vivo

We first classified yeast proteins into two groups depending on the length of the disordered termini, treating the N and C termini separately: those with short ($\leq$30 residues) and those with long (>30 residues) disordered tails (Figure 1A). The length cutoff was based on recent molecular models of the proteasome (da Fonseca et al., 2012; Lander et al., 2012; Lasker et al., 2012) and on in vitro biochemical studies using purified proteasomes showing that there is a critical minimum length of $\sim$30 residues that allows a disordered terminus of a ubiquitinated substrate to efficiently initiate degradation (Inobe et al., 2011). Indeed, analysis of the yeast data confirms that protein half-life does not depend linearly on the length of disordered segments (Figure S1B; Supplemental Experimental Procedures).

Proteins with a long disordered N terminus have a significantly shorter half-life compared to proteins with a short disordered N terminus ($p = 5 \times 10^{-6}$, Mann-Whitney *U* test, a nonparametric test for assessing whether two samples come from the same underlying distribution [H_0]; Figure 1B). The approach for measuring half-lives in yeast involved C-terminal tagging with a TAP tag, which is 186 amino acids long and largely structured. Since all proteins had identical C termini due to the TAP tag, we should see little difference in half-life between proteins with long and short C-terminal disorder as characterized from the original genome sequence. Indeed, these groups display similar distributions of protein half-life ($p = 0.99$, Mann-Whitney *U* test; Figure 1C).

In order to assess to what extent the disordered state of the N terminus affects half-life, we performed three analyses. First, we investigated proteins with a highly structured N terminus (>30 residues predicted to be structured) and found that they display a longer half-life compared to proteins with a long disordered N terminus ($p = 2 \times 10^{-7}$, Mann-Whitney *U* test; Figure 1D). Second, we classified the proteome into three groups of roughly equal size, based on their half-life: (1) short-lived proteins (half-life $\leq$ 30 min), (2) medium half-life proteins (31–70 min), and (3) long-lived proteins (>70 min) (Figure S1A). The distributions of the length of N-terminal disorder differ significantly across the three groups in a manner consistent with the above observations: proteins with a shorter half-life tend to have longer N-terminal disordered segments ($p = 3 \times 10^{-6}$, Kruskal-Wallis test, which extends the Mann-Whitney *U* test to three or more groups, Figures 1E and S1F). Again, this relationship is not true for the C terminus, because the TAP tag causes all proteins to have the same C terminus ($p = 0.2$, Kruskal-Wallis test; Figures S1E and S1F). Third, we quantified the effects of disordered segments on half-life by comparing conditional probabilities for finding proteins with and without long N-terminal disorder within specific half-life ranges. The likelihood of finding a protein with a short half-life among those that have long N-terminal disorder was two times higher than the "reverse" probability of finding proteins with long N-terminal disorder among those with short half-life (p[short half-life given long N-terminal disorder] = 0.44; p[long N-terminal disorder given short half-life] = 0.18; Tables 1A and S3A). This indicates that the presence of a long disordered N terminus often results in short half-life but proteins with short half-life need not always have a long N-terminal disordered segment. Thus, the presence of a disordered N terminus is linked to short half-life, but other properties also affect protein turnover (see Discussion).

Internal Disordered Segments Also Contribute to Short Protein Half-Life

The proteasome not only digests proteins starting from their termini but also can cleave or initiate from disordered regions in the middle of the chain (Fishbain et al., 2011; Liu et al., 2003; Piwko and Jentsch, 2006; Prakash et al., 2004; Takeuchi et al., 2007; Zhao et al., 2010). The catalytic residues for proteolysis are buried deep within the proteasome core particle, accessible only through a long narrow channel, and the same is true for the ATPase motor that drives protein substrates through the degradation channel (da Fonseca et al., 2012; Lander et al., 2012; Lasker et al., 2012). To reach these sites, a disordered segment in the middle of a protein has to be longer than a segment at a protein terminus (Fishbain et al., 2011). Therefore, to investigate whether the presence of internal

OPEN
ACCESS
CellPress

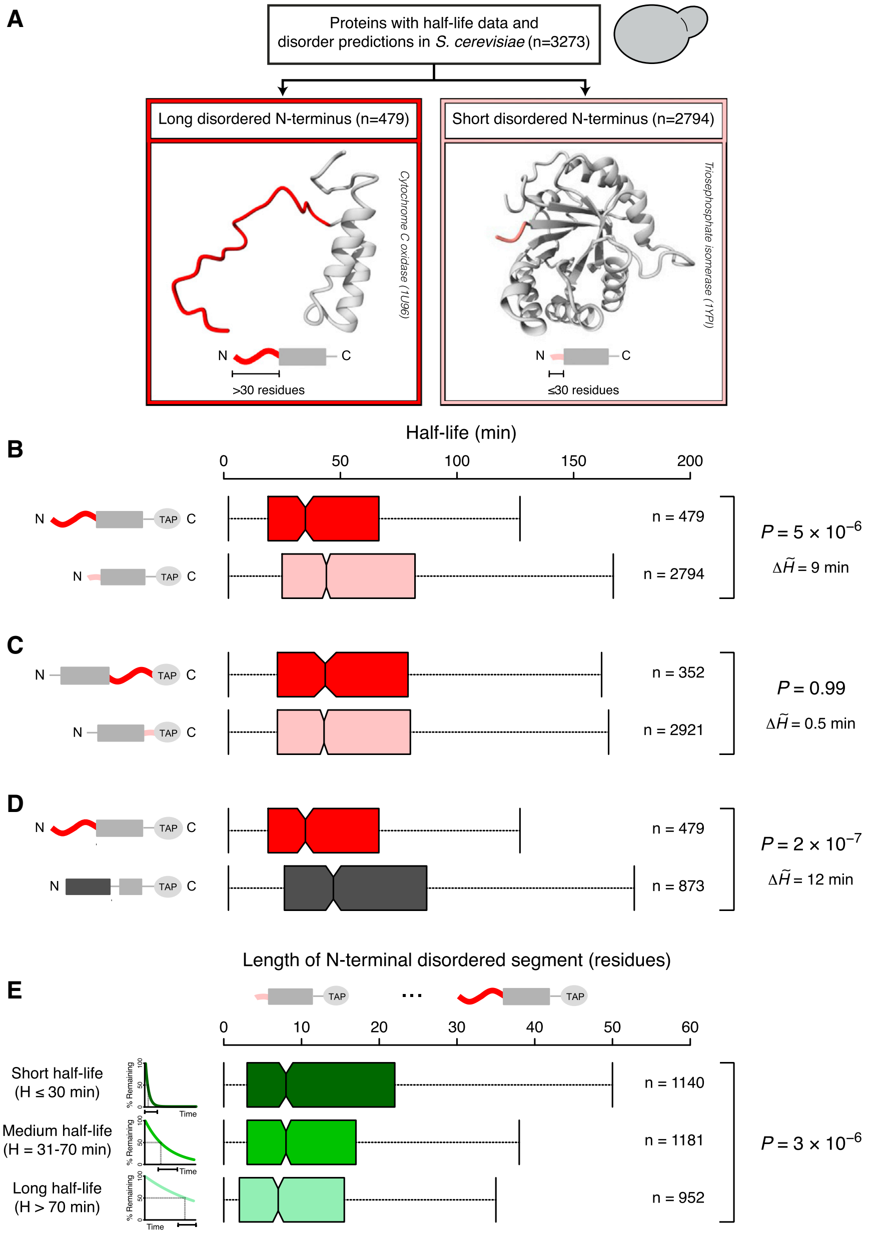

Figure 1. The Effects of Terminal Disordered Segments on Protein Half-Life

(A) A total of 3,273 yeast proteins were grouped based on the length of the disordered segment at the N terminus. Long (dark red) and short (light red) terminal disordered segments were defined as stretches of >30 and ≤30 disordered residues.

(B–D) Boxplots of protein half-life distributions. Proteins were classified based on the length of the disordered segment at the N terminus (B) or the C terminus (C) and the presence of N-terminal disordered or structured segments (D, long N-terminal structured regions [dark gray] were defined as >30 structured residues).

(E) Boxplots of the distributions of N-terminal disorder length for different half-life groups, indicated with schematic exponential degradation curves (from short half-life [dark green] to long half-life [light green]).

Central boxplot notches mark the median and the 95% confidence interval. Colored boxes represent the 50% of data points above (×0.75) and below (×0.25) the median (×0.50). Vertical lines (whiskers) connected to the boxes by the horizontal dashed lines represent the largest and the smallest nonoutlier data points. Outliers are not shown to improve visualization. p values reported are from Mann-Whitney *U* (B–D) and Kruskal-Wallis (E) tests. p values, the number of data points (n), and differences between the half-life medians of the compared groups ($\Delta\tilde{H}$) are shown to the right.

See also Figures S1 and S3 and Table S1.

disorder influences protein half-life, we identified proteasome-susceptible internal disordered segments as continuous stretches of at least 40 disordered amino acids (see Discussion). Proteins that contain such an internal disordered segment have a significantly shorter half-life than proteins that do not ($p = 3 \times 10^{-29}$, Mann-Whitney *U* test; Figure 2A). This observation is robust to our choice of cutoff used for detecting internal disordered segments, but systematically varying the length cutoff revealed that maximal difference in median half-life is obtained for a value of 40 amino acids (Table S1E). Further, the relationship is independent of N-terminal disorder, as the half-life of proteins with internal disordered segments is significantly lower than of those without, regardless of the length of the disordered terminus (Figure 2B).

To quantify the contribution of internal disorder to protein half-life, we computed conditional probabilities for finding proteins with and without internal disordered segments within specific half-life ranges. The probability of observing a protein with a short half-life among those that contain an internal disordered segment is high and comparable to the "reverse" probability of finding a protein containing an internal disordered segment among those with short half-life (p[short half-life given internal disordered segment] = 0.45; p[internal disordered segment given short half-life] = 0.49; Tables 1B and S3B). This suggests that presence or absence of an internal disordered segment is an important determinant of the half-life of a protein.

Terminal and Internal Disordered Segments Have Combined Effects on Half-Life

Interestingly, proteins with multiple internal disordered segments have even shorter half-lives than proteins with a single segment (Figures 2C and S2C). This prompted us to investigate the combinatorial effects of terminal and internal disordered segments. Indeed, proteins that have both a long terminal disordered segment and an internal disordered segment tend to have the shortest half-lives (Figure 2D). Furthermore, the

Table 1. Conditional Probabilities for Intrinsically Disordered Segments and Protein Half-Life

Half-Life / *N-Terminal Disordered Segment*	**Short (≤30 min; H_S)** 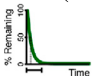	**Medium (31-70 min; H_M)** 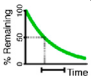	**Long (>70 min; H_L)** 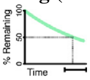	*Total*
**Long (>30 residues; N_L)** 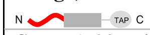	210	158	111	479
**Short (≤30residues; N_S)** 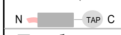	930	1023	841	2794
Total	1140	1181	952	3273

Internal Disordered Segment				
**Present (≥40 residues; I_p)** 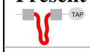	564	432	264	1260
**Absent (<40 residues; I_a)** 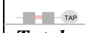	576	749	688	2013
Total	1140	1181	952	3273

	Event (E)	Condition (C)	Probability (Event I Condition) = P(E∩C)/P(C)
**A**	Short half-life	Long N-terminal disorder	$P(H_S \mid N_L) = \frac{210}{479} = 0.44$
	Long N-terminal disorder	Short half-life	$P(N_L \mid H_S) = \frac{210}{1140} = 0.18$
**B**	Short half-life	Internal disordered segment	$P(H_S \mid I_p) = \frac{564}{1260} = 0.45$
	Internal disordered segment	Short half-life	$P(I_p \mid H_S) = \frac{564}{1140} = 0.49$
**C**	Short half-life	Long N-terminal or internal disorder	$P(H_S \mid N_L \cup I_p) = \frac{653}{1513} = 0.43$
	Long N-terminal or internal disorder	Short half-life	$P(N_L \cup I_p \mid H_S) = \frac{653}{1140} = 0.57$
	Short half-life	Long N-terminal and internal disorder	$P(H_S \mid N_L \cap I_p) = \frac{121}{226} = 0.54$
	Long N-terminal and internal disorder	Short half-life	$P(N_L \cap I_p \mid H_S) = \frac{121}{1140} = 0.11$
	Short N-terminal and no internal disorder	*Short half-life*	$P(N_S \cap I_a \mid H_S) = \frac{487}{1140} = 0.43$
	Long N-terminal and internal disorder	*Long half-life*	$P(N_L \cap I_p \mid H_L) = \frac{39}{952} = 0.04$

See the top panel of this table and Figures 1 and 2 for a description of the definitions. See also Tables S3A (for part A) and S3B (for part B).

probability of having either a terminal or an internal disordered segment given that a protein has a short half-life is the highest (p[long N-terminal or internal disorder given short half-life] = 0.57; Table 1C). Consistent with this observation, we find that the probability of having both terminal and internal disordered segments among proteins with a long half-life is very low (p[long N-terminal and internal disorder given long half-life] = 0.04; Table 1C). Taken together, these results suggest that disordered segments are modular in their ability to affect protein half-life and that these segments can act in a combinatorial manner to accentuate their effects.

The Effects of Disordered Segments on Half-Life Are Independent of the Overall Disorder Degree

So far, we have investigated the effects of continuous stretches of disordered residues (i.e., disordered segments) on protein turnover. However, the fraction of disordered residues (i.e., overall degree of disorder), which is an estimate of the packing, folding, and structural stability of a protein, also correlates with half-life, although previous studies disagree on the extent of the effect (Gsponer et al., 2008; Tompa et al., 2008; Yen et al., 2008). Proteins with a greater overall disorder degree generally contain longer terminal and internal disordered segments

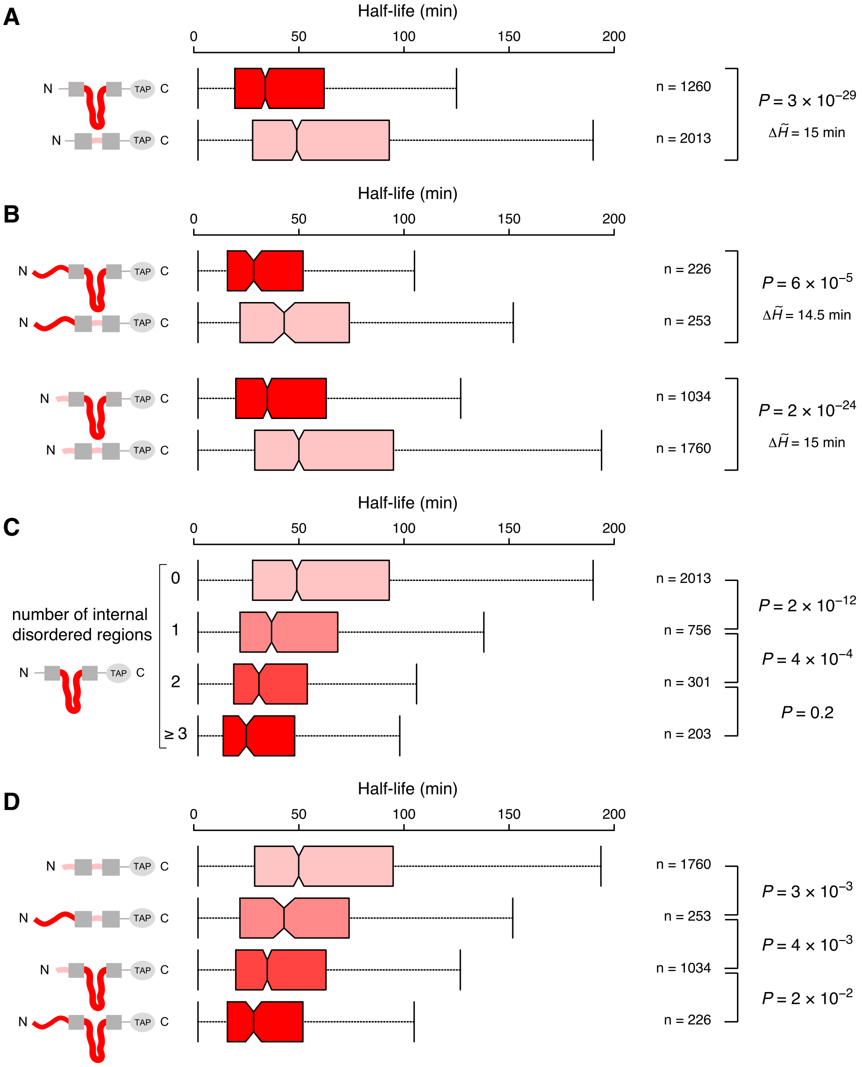

Figure 2. The Effects of Internal Disordered Segments on Protein Half-Life

(A) Boxplots of protein half-life distributions for different groups of yeast proteins that contain (dark red) or lack (light red) an internal disordered segment (defined as a continuous stretch of ≥40 disordered residues), subclassified based on (B and D) the length of N-terminal disorder (as in Figure 1: long, >30 residues or short, ≤ 30 residues) and (C) the number of internal disordered segments (from zero, top, to three or more, bottom). Each protein is present in only one category per panel. See Figure 1 for further information. See also Figures S2 and S3 and Tables S1 and S4.

(Figure S3A). To determine whether the effects of disordered segments on protein turnover (Figures 1 and 2) are independent of the overall degree of disorder, we matched proteins that have a similar fraction of disordered residues but have varying combinations of disordered segments (long or short N-terminal disorder and/or presence or absence of internal disordered segments; Supplemental Results; Figure S3B).

Comparison of the half-life distributions of proteins from different classes with similar overall disorder degrees (Figure S3C) reveals similar trends as the analysis that uses all proteins (Figure 2D): proteins with both long N-terminal and internal disordered segments typically have the shortest half-lives, followed by proteins with either long internal or long N-terminal disordered segments. Proteins without disordered segments typically have the longest half-lives. The effect sizes of the differences between the half-life distributions are comparable when using all or only proteins with matched overall disorder degree (Figure S3D, upper triangles). Furthermore, most half-life distributions are significantly different, though p values are less significant due to smaller sample sizes (Figure S3D, lower triangles).

These results indicate that long disordered segments at the N terminus or internally are important intrinsic features that contribute to shorter protein half-life in living cells and that these effects are independent of the fraction of disordered residues across the whole protein. It should, however, be noted that this does not rule out an additional effect of the overall disorder degree on half-life, i.e., among proteins that do or do not have a disordered segment, proteins with higher degrees of overall disorder tend to have a lower half-life compared to those with a lower degree of disorder (see Discussion).

Disordered Segments Have Direct Effects on Half-Life Rather than Acting Indirectly by Embedding Destruction Signals

Disordered segments could influence half-life either indirectly, by embedding short peptide motifs that serve as destruction signals such as ubiquitination sites or docking sites for ubiquitinating enzymes (Ravid and Hochstrasser, 2008), or directly, by better initiating degradation by the proteasome (Gödderz et al., 2011; Inobe et al., 2011; Peña et al., 2009; Piwko and Jentsch, 2006; Prakash et al., 2004; Takeuchi et al., 2007; Verhoef et al., 2009; Zhao et al., 2010). To investigate the indirect effects, we collected data on four known destruction signals: experimentally determined ubiquitination sites as well as predicted KEN box motifs, destruction box motifs, and PEST sequences. More than half (56%) of all proteins with long terminal or internal disordered segments do not contain any of these destruction signals in their disordered segments that could account for the short half-life (Supplemental Results). Consistently, half-life distributions of proteins with and without predicted destruction signals within the disordered regions were not significantly different (p = 0.1 for N-terminal disorder; p = 0.2 for internal disorder; Mann-Whitney *U* test; Supplemental Results). Indeed, the majority of experimentally determined ubiquitination sites involved in degradation are in structured rather than disordered regions (Hagai et al., 2011).

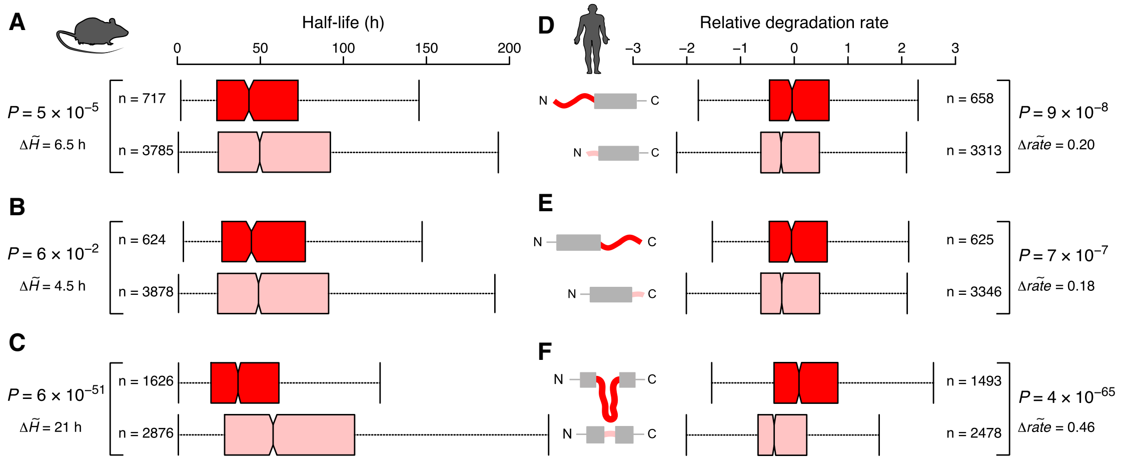

Figure 3. The Effects of Disordered Segments on Protein Turnover in Mouse and Human

Boxplots of the distributions of half-life values in *Mus musculus* (A–C), and relative degradation rates in *Homo sapiens* (D–F), for proteins with long and short N-terminal, C-terminal, and internal disordered segments. Note that the scale for protein half-life is in hours for mouse, rather than minutes as in yeast. Values are reversed for the human data: proteins with a short half-life have a high relative degradation rate. See Figure 1 for further information. See also Table S5.

Furthermore, sequence analysis revealed that disordered segments of proteins with short half-life lack enriched, uncharacterized sequence motifs that could result in rapid degradation, for example by serving as docking sites for ubiquitin ligases (Supplemental Results). Together, these findings suggest that disordered segments do not affect half-life primarily indirectly by embedding destruction motifs. Rather, the general characteristics of disordered segments seem to directly result in short half-life by forming initiation sites for degradation by the proteasome.

Disordered Segments Have Similar Effects on Protein Turnover in Mouse and Human

Given that the ubiquitin-proteasome system and the architecture of the proteasome itself are conserved from yeast to mammals (da Fonseca et al., 2012; Lasker et al., 2012), we hypothesized that the observed relationships may be evolutionarily conserved. We investigated the effects of terminal and internal disordered segments on protein degradation in mouse NIH 3T3 fibroblasts (Schwanhäusser et al., 2011) and in human THP-1 myelomonocytic leukemia cells (Kristensen et al., 2013) and found similar trends: the presence of N-terminal and internal disordered segments is linked with significantly faster protein turnover in both mouse (shorter half-lives) and human (higher degradation rates) (Figure 3; Table S5). In the mouse and human studies, protein degradation was monitored using isotope labeling and mass spectrometry, so that proteins did not need to be tagged at the either terminus (in contrast to the yeast study). Therefore, we could assess the contribution of the disordered segment at the C terminus and found that proteins with a long C-terminal disordered segment display increased turnover in mouse and human, though in mouse, the effect seems smaller than for N-terminal disorder and is not statistically significant (Figures 3B and 3E). These results collectively suggest that the effects of disordered regions on protein half-life are evolutionary conserved.

Divergence in Disordered Segments during Evolution Can Impact Protein Half-Life

Our observations suggest that protein turnover rates could be tuned by divergence in terminal or internal disordered segments during evolution (Figure 4A). To test this, we investigated protein pairs in yeast that arose from gene duplication (i.e., paralogs) (Experimental Procedures). Since paralogs are encoded within the same genome, this makes it possible to compare half-lives between evolutionarily related proteins under similar conditions. We specifically asked whether paralogs diverged in the length of N-terminal disorder or in the number of internal disordered segments (but are otherwise largely similar) and, if they did, whether this corresponded to changes in their half-life. Protein half-life data are available for both paralogs of 1,440 pairs (Table S7), and many of these paralog pairs have diverged in the length and number of terminal and internal disordered segments (Figures 4B and 4C, Tables S7 and S8).

We classified the pairs of paralogs into (1) those that during evolution maintained N-terminal disorder of roughly equal length (i.e., both proteins have a short [$\leq$30 residues] or both have a long [>30 residues] disordered segment at the N terminus; 1,049 pairs) and (2) pairs with disordered N termini of different length (i.e., one protein of the pair has a short and the other has a long disordered segment; 391 pairs). Paralogous protein pairs that diverged in the length of N-terminal disorder show significantly larger differences in half-lives than pairs that maintained roughly equal N-terminal disorder ($p = 9 \times 10^{-6}$, Mann-Whitney *U* test; Figure 4B), in a manner that agrees with the trends reported above: the protein with the longer N-terminal disordered segment usually has a shorter half-life than its paralog with a shorter disordered segment. More precisely, (1) paralogous proteins with similar length of terminal disorder tend to

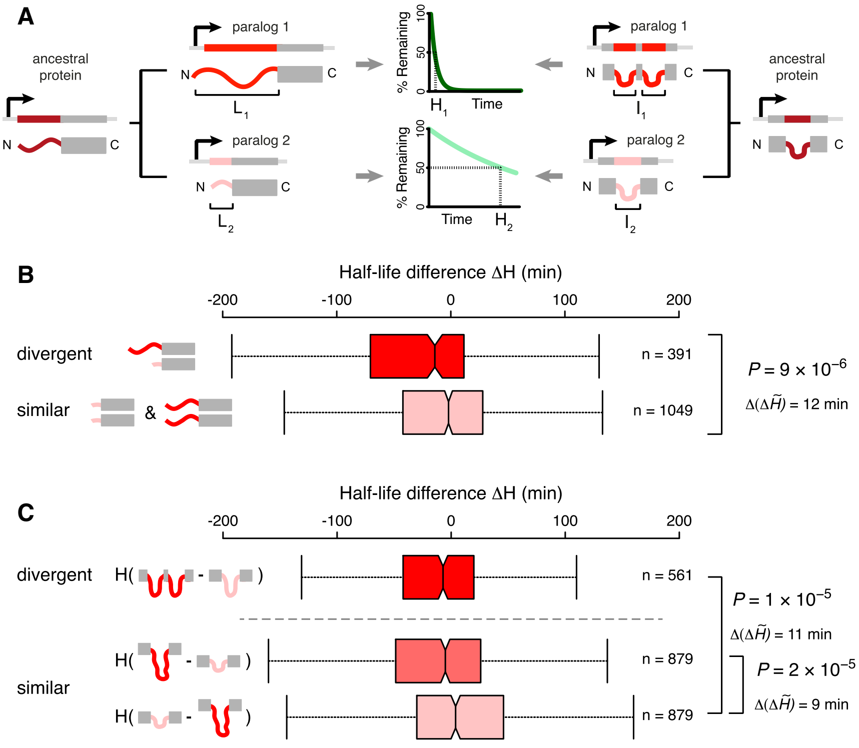

Figure 4. Divergence in Disordered Segments during Evolution Can Impact Protein Half-Life

(A) Schematic depiction of how the half-life of paralogs could be altered by changes in N-terminal and/or internal disordered segments during evolution. The dark and light green degradation curves denote a short and long half-life. This schematic is not intended to cover all possible scenarios for divergence of disordered segments between paralogs.

(B) Distributions of half-life differences (ΔH) in pairs of yeast paralogs, grouped according to the difference in the length of their N-terminal disordered segments. Top: one paralog has a short and the other paralog a long disordered N terminus (SL). Bottom: both paralogs have short (both $\leq$30 residues; SS) or both have long (both >30 residues; LL) disordered N termini.

(C) Distribution of half-life differences (ΔH) in paralog pairs, grouped according to the difference in the number of internal disordered regions (ΔI). Top: pairs where one of the two paralogs has a higher number of internal disordered segments ($\Delta I \geq 1$). Bottom: pairs with identical numbers of internal disordered segment ($\Delta I = 0$).

Each paralog pair is arranged so that $\Delta L = L_1 - L_2$ (B) and $\Delta I = I_1 - I_2$ (C) are always positive (i.e., $L_1 \geq L_2$ and $I_1 \geq I_2$). This order is used for the ΔH calculation (that is, the half-life of the paralog with the shortest N-terminal disorder, or the smallest number of internal disordered segments, will be subtracted from the half-life of the other one; $\Delta H = H_1 - H_2$). As a result, ΔH will be negative for pairs where an increase in N-terminal or internal disorder coincides with a shorter half-life (Experimental Procedures). For $\Delta I = 0$ (C, bottom), two ΔH distributions were obtained by ordering the paralogs within a pair according to the total length of all internal disordered segments (increasing and decreasing to simulate gain and loss of internal disorder length during evolution; Table S6A). See Figure 1 for further information. See also Figure S4 and Table S6.

have similar half-life values (median difference in half-life is close to zero; Figure 4B, bottom boxplot; Table S6A), and (2) the half-life of proteins with longer N-terminal disordered regions tends to be 14 min shorter (median) than that of their paralogous partners (Figure 4B, top boxplot; Table S6A). The converse is also true, as paralogous pairs with large half-life changes show a large divergence in the length of N-terminal disorder (Supplemental Results; Figure S4A). A 14 min difference in half-life between paralogs is substantial in the context of yeast biology, as this is comparable to the time from division to budding (G1 phase) in laboratory strains growing exponentially in rich media at 30°C, which is 15–37 min (Di Talia et al., 2007). Thus, altered half-life due to divergence in the length of terminal disorder could have a significant impact on the duration for which a protein can impart its function in a cell and thus affect cellular behavior.

Paralogous proteins that differ in the number of internal disordered segments also show significantly larger changes in half-life than pairs with the same number of internal disordered regions ($p = 1 \times 10^{-5}$, Mann-Whitney *U* test; Figure 4C). The half-life of proteins with more internal disordered regions tends to be 7 min shorter (median) than that of their paralogs (Table S6A), which again can be a considerable amount of time considering the doubling time of yeast. In paralogous pairs that have the same number of internal disordered segments but diverged in the total internal disorder length (i.e., the sum of all internal disordered segments), the half-life of the protein with the longer total internal disorder also tends to be shorter (median half-life difference is 5 min; Table S6A).

Analysis of conditional probability values allowed us to quantify the trends (Tables 2 and S8). The majority (73%) of paralogous pairs that diverged in the length of terminal or number of internal disordered segments show a consistent change in half-life: the paralog with the longest terminal disordered segment or largest number of internal disordered segments tends to have the shorter half-life (p[shorter half-life given divergence of N-terminal or internal disorder] = 0.73; Table 2C). This effect is large even if only segments at the N terminus or only internal segments are considered (p [shorter half-life given divergence of N-terminal disorder] = 0.64 and p[shorter half-life given divergence of internal disorder] = 0.58; Tables 2A and 2B). Again, the converse is also true as the probability of observing a paralogous pair that has diverged in both terminal and internal disorder, and in which the paralog with the longest terminal disordered segment and most internal disordered segments has the longer half-life, is very small (p[divergence of N-terminal and internal disorder given longer half-life] = 0.01; Table 2C). Taken together, the results suggest that the gain or loss of long terminal or internal disordered segments can significantly influence the half-life of a protein upon gene duplication during evolution. Thus evolution of intrinsic features such as disordered segments may be an important contributor to the degradation rate of proteins.

Table 2. Conditional Probabilities for Intrinsically Disordered Segments and Protein Half-Life in Pairs of Paralogs

	Event (E)	Condition (C)	Probability (Event | Condition) = P(E∩C)/P(C)
A	Shorter half-life	divergence of N-terminal disorder	$P(\Delta H<0\text{min}\|N_{SL}) = \frac{252}{391} = 0.64$
	Divergence of N-terminal disorder	shorter half-life	$P(N_{SL}\|\Delta H<0\text{min}) = \frac{252}{787} = 0.32$
B	Shorter half-life	divergence of internal disordered segments	$P(\Delta H<0\text{min}\|I_{n\to n+x}) = \frac{323}{561} = 0.58$
	Divergence of internal disordered segments	shorter half-life	$P(I_{n\to n+x}\|\Delta H<0\text{min}) = \frac{323}{799} = 0.40$
C	Shorter half-life	divergence of N-terminal or internal disordered segments	$P(\Delta H<0\text{min}\|N_{SL}\cup I_{n\to n+x}) = \frac{542}{741} = 0.73$
	Divergence of N-terminal or internal disordered segments	shorter half-life	$P(N_{SL}\cup I_{n\to n+x}\|\Delta H<0\text{min}) = \frac{542}{1176} = 0.46$
	Shorter half-life	divergence of N-terminal and internal disordered segments	$P(\Delta H<0\text{min}\|N_{SL}\cap I_{n\to n+x}) = \frac{33}{211} = 0.16$
	Divergence of N-terminal and internal disordered segments	shorter half-life	$P(N_{SL}\cap I_{n\to n+x}\|\Delta H<0\text{min}) = \frac{33}{1176} = 0.03$
	Divergence of N-terminal and internal disordered segments	longer half-life	$P(N_{SL}\cap I_{n\to n+x}\|\Delta H>0\text{min}) = \frac{11}{1013} = 0.01$

See Figure 4 for a description of the definitions. N_{SL} denotes pairs where one paralog has a short and the other paralog a long N-terminal disordered segment. $I_{n => n+x}$ denotes pairs where one of the two paralogs has a higher number of internal disordered segments ($\Delta I \geq 1$). See also Tables S8A (for part A) and S8B (for part B).

Functional Analysis and Literature Evidence Support the Role of Disordered Segments in Governing Protein Half-Life and Phenotype

Disordered segments that affect half-life could be important for governing phenotypes, because precise protein turnover is important for many cellular processes. An analysis of function annotations of proteins with long N-terminal or internal disordered segments revealed enrichment for protein kinases and phosphoproteins and associations with regulatory and transcription functions, as well as cell-cycle processes (Tables S1F and S4B). Paralogs that have diverged in terminal or internal disordered segments have similar functions and are additionally involved in, for example, ATP and nucleotide binding and ubiquitin conjugation activities (Table S6C). These are all functions involved in signaling and regulation, where alteration of protein half-life can significantly affect the duration of activity of the protein and thereby impact cellular phenotype (Legewie et al., 2008) (see Discussion).

A literature search revealed several examples where changes in disordered segments lead to phenotypic differences through altered protein half-life. Stabilizing the half-life of the yeast kinase Ime2, a positive regulator of meiosis, by deletion of an internal disordered region results in altered sporulation efficiency (Guttmann-Raviv et al., 2002). Similarly, deletion of a highly disordered 47-amino-acid stretch at the N terminus of yeast Cdc6 prevents its degradation, although in this case, the deletion also abolishes the interaction with a ubiquitin ligase complex (Drury et al., 1997). Deletion of the first 31 residues of the human nuclear receptor Nurr1 significantly reduces its degradation by the ubiquitin-proteasome pathway and consequently leads to increased activation as a transcription factor (Alvarez-Castelao et al., 2013). Interestingly, the deleted region corresponds completely to a putative disordered segment at the N terminus and the size of the deletion could now explain the effects on half-life. These selected examples illustrate the importance of disordered segments for maintaining correct protein turnover.

DISCUSSION

Ubiquitination by E3 ligases has a dominant role in deciding when a protein gets targeted for proteasomal degradation, but it has remained unclear how intrinsic features affect the lifetime of a protein and whether such features have been exploited to alter half-life during evolution. Here, we uncovered genome-scale principles of how intrinsically disordered segments influence protein turnover in the cell and during evolution. On a genomic scale, in vivo, sufficiently long disordered regions at the termini or in the middle of proteins can directly decrease half-life (Figure 5). A large number of control calculations confirmed that the reported trends are independent of confounding factors, such as the cutoffs used to group the proteins, the disorder prediction method, the statistical tests used, protein abundance and length, subcellular localization, membrane proteins, and the nature of the N-terminal residue (Supplemental Results). Finally, we found that changes in the length and number of disordered segments upon gene duplication are linked with altered half-life, suggesting

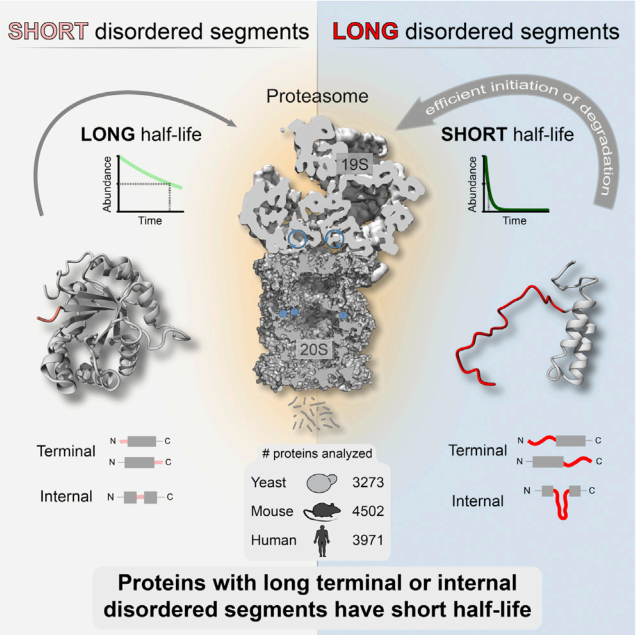

Figure 5. Concept Describing the General Relationship between the Presence of Long Terminal or Internal Disordered Segments and Protein Half-Life

Disordered segments influence protein half-life by permitting efficient initiation of degradation by the proteasome. Ubiquitination and other factors contributing to substrate targeting to the proteasome are not depicted.

that such variation can contribute to the tuning of half-life during evolution.

The Structure and Composition of the Proteasome Suggest Molecular Mechanisms to Explain the Observations

The structure of the 19S regulatory particle (da Fonseca et al., 2012; Lander et al., 2012; Lasker et al., 2012) provides insights into the mechanisms by which disordered segments may increase the efficiency of proteasomal degradation and affect protein half-life. The distance between the two ubiquitin receptors, Rpn10 and Rpn13, and the ATPase unfolding channel is ~70–80 Å. The essential deubiquitinating enzyme Rpn11 sits ~60 Å from the ATPase ring. A terminal disordered segment of 30 residues would comfortably span these distances and could serve as a degradation initiation site. Similarly, 40 residues would be enough for an internal disordered segment to reach into the ATPase ring of the regulatory particle, even when folding back on itself. The precise distance requirements for a disordered segment to serve as an initiation site will depend on specific properties of the proteasome and the geometry and binding position of the substrate. For example, at least five different substrate receptors associate with the yeast proteasome, and some of them exhibit extensive conformational flexibility (Finley, 2009). Substrate-specific aspects that affect the distances include the state of the termini, which are frequently subject to maturation through cleavage and trimming (Lange and Overall, 2013), and properties of the polyubiquitin tag such as the linkage type (e.g., K48 and K11) and number of ubiquitin moieties (Komander and Rape, 2012), and the attachment point to the substrate (Hagai et al., 2011; Inobe et al., 2011). This could explain why, with in vivo data for thousands of different proteins, we do not observe a strict length cutoff for when disordered segments influence protein half-life: cutoffs of about 30 terminal and 40 internal disordered residues produce the largest differences between the half-lives of proteins with and without disordered segments, but shorter and longer segments also contribute to shorter half-life (Table S1E). Thus, individual proteins are likely to have specific length requirements of disordered segments that depend on a variety of factors and contribute to the range of lengths at which disordered segments decrease protein half-life on a global scale.

The ATP-independent regulators PA28 and P200 can also facilitate opening of the 20S proteasome entry gate and contribute to substrate degradation (Stadtmueller and Hill, 2011). The ATPase complex p97/VCP perhaps serves as an alternative cap that directly binds the 20S core particle as well (Barthelme and Sauer, 2012). All these complexes may have different requirements for disordered segments in the substrate proteins. In fact, it has been suggested that p97/VCP may unfold proteins lacking disordered regions (Beskow et al., 2009). In vitro, the 20S proteasome core particle by itself can degrade highly disordered proteins in a process termed degradation by default and it may also be able to do so in vivo (Tsvetkov et al., 2008). The average distance between the entry pore and the proteolytic sites in the 20S core particle is ~70 Å (da Fonseca et al., 2012; Lasker et al., 2012). An internal disordered segment of at least 40 residues is able to span twice this distance and thus could be cleaved by the core particle alone (Figure S2D). Thus, proteins with disordered segments of specific length may be processed quickly due to efficient initiation of degradation as discussed.

The overall disorder degree of a substrate might further affect its half-life. Upon initiation of degradation, the proteasome may quickly degrade proteins with high overall levels of disorder, because its ATPase subunits spend less time to unfold these disordered proteins once they are engaged compared to proteins of similar length that are structured and need to be unfolded before they can be processed. Indeed, biochemical evidence suggests increasingly structured and stable substrates have higher turnover times and energy costs (Henderson et al., 2011; Peth et al., 2013).

Disordered Segments Influence Half-Life as an Intrinsic Feature that Can Be Modulated by Other Mechanisms

Although proteins with long terminal or internal disordered segments tend to have a short half-life, various factors can increase or decrease the half-life of individual proteins (see also Supplemental Discussion). For example, the presence of a highly structured N-terminal domain may shield proteins with internal disordered segments from degradation (Simister et al., 2011). Disordered proteins may also be protected by forming protein complexes or through interactions with other proteins. For instance, several specialized proteins have been shown to bind to and stabilize disordered proteins (Tsvetkov et al., 2009). Furthermore, specific low-complexity sequences or tandem

repeats in the degradation initiation site can attenuate initiation or progression of degradation, thereby affecting substrate half-life (Sharipo et al., 1998; Tian et al., 2005; Zhang and Coffino, 2004). This is consistent with the idea that, although disordered segments are all similar in that they lack the ability to independently fold into a compact structure, many types of sequences exist within this definition that have different biophysical and conformational characteristics. For example, some disordered sequences are relatively globular and collapsed while others are expanded, and this is determined by the overall charge and sequence composition of the disordered region (Mao et al., 2013). Indeed, the proteasome has clear amino acid sequence preferences as degradation of model substrates that differ only in their disordered initiation regions varies over at least an order of magnitude (Fishbain et al., 2014). The broad distributions of half-lives observed in our study support this, as they reflect the combined properties of many possible subtypes of disordered segments, some of which are able to efficiently initiate degradation, while others may not.

In short, one can distinguish at least three distinct determinants of protein half-life. (1) Sequence motifs and the presence of regulatory proteins such as ubiquitin ligases contribute to the overall half-life of a protein by determining when substrates will be ubiquitinated and hence targeted to the proteasome. (2) Upon recognition of the ubiquitinated substrate by the proteasome, the presence of disordered segments of sufficient size either at the terminus or internally may facilitate efficient degradation initiation, thereby leading to lower half-life. (3) The overall degree of disorder may contribute to a general trend in lowering half-life by increasing the processivity of degradation upon engagement (after recognition and initiation) by the proteasome. Thus, our observations suggest that disordered segments influence protein half-life as an underlying factor that can be modulated by other cellular mechanisms, sequence determinants and the structural stability of the substrate.

Disordered Segments Could Influence the Dynamics and Regulation of Signaling Pathways

Disordered regions are prominent in regulatory and signaling proteins (Tables S1F, S4B, and S6C) (van der Lee et al., 2014). Since divergence in disordered segments may affect protein half-life, this could influence the response kinetics of signaling and regulatory pathways involving such proteins (see also Supplemental Discussion). In fact, among the paralogous pairs that show the largest divergence in the length of N-terminal disorder and half-life in yeast (Tables S6C and S7), there are several regulatory protein kinases such as MAP kinases (*MKK1*, *MKK2*, *HOG1*, and *STE7*), serine/threonine kinases (*YPK1*, *YPK2*, and *KIN28*), and cyclin-dependent kinases (*PHO85* and *CAK1*). Paralogs that have diverged in terminal or internal disordered segments are generally enriched in nucleotide binding, kinase regulatory activity, and phosphoproteins. Alterations in the degradation rate of kinases, for instance, can have significant implications for the dynamics of signaling networks (Legewie et al., 2008; Purvis and Lahav, 2013). Such effects have been shown for the yeast kinase Ime2 (Guttmann-Raviv et al., 2002) and mouse transcription factor Hes7 (Hirata et al., 2004), where mutations in disordered segments lead to changes in protein half-life, which in turn severely deregulate signaling and development, respectively.

Our observations raise the possibility that proteins with a long terminal disordered segment might be better presented as antigens to the immune system. This is because the immunoproteasome, a variant of the canonical proteasome, may better process such proteins into peptides for presentation by the major histocompatibility complex (MHC) molecules (Groettrup et al., 2010). In line with this idea, it has been shown that (1) N-extended epitopes are efficiently processed by the immunoproteasome and serve as better substrates for antigen presentation (Cascio et al., 2001) and (2) the presence of a disordered region determines the direction of degradation, which in turn determines the spectrum of generated peptides (Berko et al., 2012). It is tempting to speculate that one could improve vaccine efficiency by adding or extending terminal disordered regions to epitope-containing proteins. Our findings also call for careful interpretation of half-life measurements made on proteins that are tagged at their termini using constructs with a varying degree of structure or intrinsic disorder (e.g., GFP, TAP tag, His-tag).

Divergence in Disordered Segments Provides a Means for Tuning Protein Half-Life during Evolution and Could Generate Phenotypic Variation

We observed that divergence in disordered regions might influence protein half-life among paralogs. An outstanding question is whether such changes in half-life through divergence of disordered segments are under selective pressure. Natural variation leading to alteration of disordered regions may provide a simple means for regulatory subfunctionalization of paralogous proteins upon gene duplication. It also suggests a mechanism for divergence of half-life among orthologous proteins between species. Thus, while it is clear that the emergence of destruction signals such as ubiquitination sites and dedicated ubiquitin ligases affect targeting of a protein for degradation, variation in disordered segments may provide a simple evolutionary mechanism for fine-tuning protein turnover rates.

Several genetic and molecular mechanisms may generate diversity in terminal or internal disordered segments. These include repeat expansion, alternative splicing, and alternative transcription start sites, all of which can influence the length of terminal and/or internal disorder of protein products, thereby potentially influencing the half-life. This idea is supported by the observation that protein disorder is common in insertions and deletions (Light et al., 2013). Furthermore, given that in multicellular eukaryotes (1) alternative transcription start sites commonly generate variation in N termini (Carninci et al., 2006) and (2) alternatively spliced exons are enriched in intrinsic disorder (Buljan et al., 2013), it is likely that such events that generate diversity in protein sequences in different cell types within an individual will have an effect on protein half-life. Similarly, given that disordered regions often contain homopolymeric repeat sequences (Tompa, 2003), and because tandem repeats in DNA sequences can lead to expansion or deletion of genetic material through strand slippage during replication (Levinson and Gutman, 1987), it is plausible that individuals in a population harbor genetic variants that code for proteins with altered length of disordered segments and thus have different half-lives. Changes

in protein turnover in turn may disturb protein abundance and could lead to disease (Babu et al., 2011; Hirata et al., 2004; Yang et al., 2012), especially in the case of pleiotropic, regulatory, or signaling proteins. Thus mechanisms that generate diversity in the length or number of disordered segments could serve as a source of genetic variation that may have important phenotypic consequences.

EXPERIMENTAL PROCEDURES

Protein Half-Life Data and Calculation of Disordered Segments

Protein half-life data and other data (Table S1A) were collected for yeast (*Saccharomyces cerevisiae*), mouse, and human. Intrinsic disorder was predicted for all reviewed protein sequences of these organisms (downloaded from UniProtKB/Swiss-Prot; http://www.uniprot.org/) using three complementary methods: DISOPRED2, IUPRED long, and PONDR VLS1. The presence and length of N-terminal, C-terminal, and internal disordered segments were then calculated using different algorithms and integrated with the half-life data. Proteomes were classified into groups according to the length of disordered segments: (1) proteins with short and long disordered termini (length cutoff 30 residues, treating the N and C termini separately) and (2) proteins with and without internal disordered segments (at least 40 disordered residues). The overall degree of disorder of a protein was calculated as the fraction of disordered residues (number of disordered residues divided by sequence length). The distributions of half-life values and protein disorder were analyzed using appropriate statistical tests.

See the Supplemental Experimental Procedures for more details.

Paralog Data and Calculations

Yeast paralog pairs were obtained from an all-against-all sequence comparison using BLASTClust (Altschul et al., 1990). More divergent paralogs from the yeast whole-genome duplication event (Wolfe and Shields, 1997) were added to the list. To calculate the differences in half-life (ΔH) and N-terminal disorder length (ΔL) between the individual proteins in a paralog pair, ΔL is defined to be always positive and obtained by subtracting the N-terminal disorder length of paralog 2 from the N-terminal disorder length of paralog 1 ($\Delta L = L1 - L2$; $L1 \geq L2$). To calculate ΔH, the order of paralogs in a pair is maintained, so that ΔH can be positive or negative ($\Delta H = H1 - H2$). Thus, ΔH is negative whenever the relationship "longer disordered N terminus = shorter half-life" holds true. Similarly, the difference in the number of internal disordered segments ΔI is defined to always be positive ($\Delta I = I1 - I2$; $I1 \geq I2$), and ΔH is calculated accordingly. Paralog pairs were separated into categories according to the divergence in N-terminal (pairs that maintained or that diverged N-terminal disorder) or internal disordered segments (pairs with an identical or different number of segments).

See the Supplemental Experimental Procedures for more details.

SUPPLEMENTAL INFORMATION

Supplemental Information includes Supplemental Results, Supplemental Discussion, Supplemental Experimental Procedures, four figures, and eight tables and can be found with this article online at http://dx.doi.org/10.1016/j.celrep.2014.07.055.

AUTHOR CONTRIBUTIONS

R.v.d.L. and M.M.B. designed the project and analyzed and interpreted the results. R.v.d.L. performed the majority of the calculations. B.L. performed additional calculations, and K.K., J.G., N.S.d.G., and M.F. carried out specific analyses. B.L., K.K., M.A.H., M.F., and A.M. assisted in interpretation. R.v.d.L. and M.M.B. wrote the manuscript with help from B.L., M.A.H., M.F., and A.M.

ACKNOWLEDGMENTS

We acknowledge the Medical Research Council (U105185859). We thank A. Bertolotti, C. Chothia, C. Ravarani, D. Komander, G. Chalancon, J. Marsh, R. ter Horst, M. Buljan, R. Hegde, R. Weatheritt, S. Balaji, S. Chavali, S. Teichmann, and the anonymous reviewers for their comments on this work. We acknowledge financial support from HFSP (RGY0073/2010, R.v.d.L., B.L., and M.M.B.), the Virgo consortium funded by the Dutch government (FES0908) and the Netherlands Genomics Initiative (050-060-452; R.v.d.L. and M.A.H.), the Herchel Smith Research Studentship Fund (B.L.), the LMB International PhD Programme (K.K.), an EMBO STF 312-2011 and Momentum award (LP2012-41/2012) by the Hungarian Academy of Sciences (M.F.), FEBS and FP7-PEOPLE-2012-IEF 299105 fellowships (N.S.d.G.), PrioNet Canada and UBC (J.G.), NIH (R01GM63004 and U54GM105816, A.M.), and the Welch Foundation (F-1817, A.M.). M.M.B. acknowledges the MRC, EMBO Young Investigator Programme, Trinity College, and ERASysBio+ for support and funding his research program.

Received: June 6, 2013
Revised: June 12, 2014
Accepted: July 29, 2014
Published: September 11, 2014

REFERENCES

Altschul, S.F., Gish, W., Miller, W., Myers, E.W., and Lipman, D.J. (1990). Basic local alignment search tool. J. Mol. Biol. *215*, 403–410.

Alvarez-Castelao, B., Losada, F., Ahicart, P., and Castaño, J.G. (2013). The N-terminal region of Nurr1 (a.a 1-31) is essential for its efficient degradation by the ubiquitin proteasome pathway. PLoS ONE *8*, e55999.

Babon, J.J., McManus, E.J., Yao, S., DeSouza, D.P., Mielke, L.A., Sprigg, N.S., Willson, T.A., Hilton, D.J., Nicola, N.A., Baca, M., et al. (2006). The structure of SOCS3 reveals the basis of the extended SH2 domain function and identifies an unstructured insertion that regulates stability. Mol. Cell *22*, 205–216.

Babu, M.M., van der Lee, R., de Groot, N.S., and Gsponer, J. (2011). Intrinsically disordered proteins: regulation and disease. Curr. Opin. Struct. Biol. *21*, 432–440.

Babu, M.M., Kriwacki, R.W., and Pappu, R.V. (2012). Structural biology. Versatility from protein disorder. Science *337*, 1460–1461.

Barthelme, D., and Sauer, R.T. (2012). Identification of the Cdc48●20S proteasome as an ancient AAA+ proteolytic machine. Science *337*, 843–846.

Belle, A., Tanay, A., Bitincka, L., Shamir, R., and O'Shea, E.K. (2006). Quantification of protein half-lives in the budding yeast proteome. Proc. Natl. Acad. Sci. USA *103*, 13004–13009.

Berko, D., Tabachnick-Cherny, S., Shental-Bechor, D., Cascio, P., Mioletti, S., Levy, Y., Admon, A., Ziv, T., Tirosh, B., Goldberg, A.L., and Navon, A. (2012). The direction of protein entry into the proteasome determines the variety of products and depends on the force needed to unfold its two termini. Mol. Cell *48*, 601–611.

Beskow, A., Grimberg, K.B., Bott, L.C., Salomons, F.A., Dantuma, N.P., and Young, P. (2009). A conserved unfoldase activity for the p97 AAA-ATPase in proteasomal degradation. J. Mol. Biol. *394*, 732–746.

Buljan, M., Chalancon, G., Dunker, A.K., Bateman, A., Balaji, S., Fuxreiter, M., and Babu, M.M. (2013). Alternative splicing of intrinsically disordered regions and rewiring of protein interactions. Curr. Opin. Struct. Biol. *23*, 443–450.

Carninci, P., Sandelin, A., Lenhard, B., Katayama, S., Shimokawa, K., Ponjavic, J., Semple, C.A., Taylor, M.S., Engström, P.G., Frith, M.C., et al. (2006). Genome-wide analysis of mammalian promoter architecture and evolution. Nat. Genet. *38*, 626–635.

Cascio, P., Hilton, C., Kisselev, A.F., Rock, K.L., and Goldberg, A.L. (2001). 26S proteasomes and immunoproteasomes produce mainly N-extended versions of an antigenic peptide. EMBO J. *20*, 2357–2366.

Ciechanover, A. (2012). Intracellular protein degradation: from a vague idea thru the lysosome and the ubiquitin-proteasome system and onto human diseases and drug targeting. Biochim. Biophys. Acta *1824*, 3–13.

da Fonseca, P.C., He, J., and Morris, E.P. (2012). Molecular model of the human 26S proteasome. Mol. Cell *46*, 54–66.

Di Talia, S., Skotheim, J.M., Bean, J.M., Siggia, E.D., and Cross, F.R. (2007). The effects of molecular noise and size control on variability in the budding yeast cell cycle. Nature *448*, 947–951.

Dosztányi, Z., Csizmók, V., Tompa, P., and Simon, I. (2005). The pairwise energy content estimated from amino acid composition discriminates between folded and intrinsically unstructured proteins. J. Mol. Biol. *347*, 827–839.

Drury, L.S., Perkins, G., and Diffley, J.F. (1997). The Cdc4/34/53 pathway targets Cdc6p for proteolysis in budding yeast. EMBO J. *16*, 5966–5976.

Finley, D. (2009). Recognition and processing of ubiquitin-protein conjugates by the proteasome. Annu. Rev. Biochem. *78*, 477–513.

Fishbain, S., Prakash, S., Herrig, A., Elsasser, S., and Matouschek, A. (2011). Rad23 escapes degradation because it lacks a proteasome initiation region. Nat. Commun. *2*, 192.

Fishbain, S., Inobe, T., Israeli, E., Zokarkar, A., Chavali, S., Babu, M.M., and Matouschek, A. (2014). The sequence composition of disordered regions affects protein half-life by controlling the initiation step of proteasomal degradation. Nat. Struct. Mol. Biol..

Gödderz, D., Schäfer, E., Palanimurugan, R., and Dohmen, R.J. (2011). The N-terminal unstructured domain of yeast ODC functions as a transplantable and replaceable ubiquitin-independent degron. J. Mol. Biol. *407*, 354–367.

Goldberg, A.L. (2003). Protein degradation and protection against misfolded or damaged proteins. Nature *426*, 895–899.

Groettrup, M., Kirk, C.J., and Basler, M. (2010). Proteasomes in immune cells: more than peptide producers? Nat. Rev. Immunol. *10*, 73–78.

Gsponer, J., Futschik, M.E., Teichmann, S.A., and Babu, M.M. (2008). Tight regulation of unstructured proteins: from transcript synthesis to protein degradation. Science *322*, 1365–1368.

Guttmann-Raviv, N., Martin, S., and Kassir, Y. (2002). Ime2, a meiosis-specific kinase in yeast, is required for destabilization of its transcriptional activator, Ime1. Mol. Cell. Biol. *22*, 2047–2056.

Hagai, T., Azia, A., Tóth-Petróczy, Á., and Levy, Y. (2011). Intrinsic disorder in ubiquitination substrates. J. Mol. Biol. *412*, 319–324.

Henderson, A., Erales, J., Hoyt, M.A., and Coffino, P. (2011). Dependence of proteasome processing rate on substrate unfolding. J. Biol. Chem. *286*, 17495–17502.

Hershko, A., and Ciechanover, A. (1998). The ubiquitin system. Annu. Rev. Biochem. *67*, 425–479.

Hirata, H., Bessho, Y., Kokubu, H., Masamizu, Y., Yamada, S., Lewis, J., and Kageyama, R. (2004). Instability of Hes7 protein is crucial for the somite segmentation clock. Nat. Genet. *36*, 750–754.

Hutchins, A.P., Liu, S., Diez, D., and Miranda-Saavedra, D. (2013). The repertoires of ubiquitinating and deubiquitinating enzymes in eukaryotic genomes. Mol. Biol. Evol. *30*, 1172–1187.

Inobe, T., Fishbain, S., Prakash, S., and Matouschek, A. (2011). Defining the geometry of the two-component proteasome degron. Nat. Chem. Biol. *7*, 161–167.

Irimia, M., Denuc, A., Ferran, J.L., Pernaute, B., Puelles, L., Roy, S.W., Garcia-Fernàndez, J., and Marfany, G. (2012). Evolutionarily conserved A-to-I editing increases protein stability of the alternative splicing factor Nova1. RNA Biol. *9*, 12–21.

Komander, D., and Rape, M. (2012). The ubiquitin code. Annu. Rev. Biochem. *81*, 203–229.

Komander, D., Clague, M.J., and Urbé, S. (2009). Breaking the chains: structure and function of the deubiquitinases. Nat. Rev. Mol. Cell Biol. *10*, 550–563.

Kristensen, A.R., Gsponer, J., and Foster, L.J. (2013). Protein synthesis rate is the predominant regulator of protein expression during differentiation. Mol. Syst. Biol. *9*, 689.

Lakin, N.D., and Jackson, S.P. (1999). Regulation of p53 in response to DNA damage. Oncogene *18*, 7644–7655.

Lander, G.C., Estrin, E., Matyskiela, M.E., Bashore, C., Nogales, E., and Martin, A. (2012). Complete subunit architecture of the proteasome regulatory particle. Nature *482*, 186–191.

Lange, P.F., and Overall, C.M. (2013). Protein TAILS: when termini tell tales of proteolysis and function. Curr. Opin. Chem. Biol. *17*, 73–82.

Lasker, K., Förster, F., Bohn, S., Walzthoeni, T., Villa, E., Unverdorben, P., Beck, F., Aebersold, R., Sali, A., and Baumeister, W. (2012). Molecular architecture of the 26S proteasome holocomplex determined by an integrative approach. Proc. Natl. Acad. Sci. USA *109*, 1380–1387.

Legewie, S., Herzel, H., Westerhoff, H.V., and Blüthgen, N. (2008). Recurrent design patterns in the feedback regulation of the mammalian signalling network. Mol. Syst. Biol. *4*, 190.

Levinson, G., and Gutman, G.A. (1987). Slipped-strand mispairing: a major mechanism for DNA sequence evolution. Mol. Biol. Evol. *4*, 203–221.

Light, S., Sagit, R., Ekman, D., and Elofsson, A. (2013). Long indels are disordered: a study of disorder and indels in homologous eukaryotic proteins. Biochim. Biophys. Acta *1834*, 890–897.

Liu, C.W., Corboy, M.J., DeMartino, G.N., and Thomas, P.J. (2003). Endoproteolytic activity of the proteasome. Science *299*, 408–411.

Mao, A.H., Lyle, N., and Pappu, R.V. (2013). Describing sequence-ensemble relationships for intrinsically disordered proteins. Biochem. J. *449*, 307–318.

Obradovic, Z., Peng, K., Vucetic, S., Radivojac, P., and Dunker, A.K. (2005). Exploiting heterogeneous sequence properties improves prediction of protein disorder. Proteins *61* (*Suppl 7*), 176–182.

Pagano, M., Tam, S.W., Theodoras, A.M., Beer-Romero, P., Del Sal, G., Chau, V., Yew, P.R., Draetta, G.F., and Rolfe, M. (1995). Role of the ubiquitin-proteasome pathway in regulating abundance of the cyclin-dependent kinase inhibitor p27. Science *269*, 682–685.

Peña, M.M., Melo, S.P., Xing, Y.Y., White, K., Barbour, K.W., and Berger, F.G. (2009). The intrinsically disordered N-terminal domain of thymidylate synthase targets the enzyme to the ubiquitin-independent proteasomal degradation pathway. J. Biol. Chem. *284*, 31597–31607.

Peth, A., Nathan, J.A., and Goldberg, A.L. (2013). The ATP costs and time required to degrade ubiquitinated proteins by the 26 S proteasome. J. Biol. Chem. *288*, 29215–29222.

Piwko, W., and Jentsch, S. (2006). Proteasome-mediated protein processing by bidirectional degradation initiated from an internal site. Nat. Struct. Mol. Biol. *13*, 691–697.

Prakash, S., Tian, L., Ratliff, K.S., Lehotzky, R.E., and Matouschek, A. (2004). An unstructured initiation site is required for efficient proteasome-mediated degradation. Nat. Struct. Mol. Biol. *11*, 830–837.

Purvis, J.E., and Lahav, G. (2013). Encoding and decoding cellular information through signaling dynamics. Cell *152*, 945–956.

Ramakrishna, S., Suresh, B., Lim, K.H., Cha, B.H., Lee, S.H., Kim, K.S., and Baek, K.H. (2011). PEST motif sequence regulating human NANOG for proteasomal degradation. Stem Cells Dev. *20*, 1511–1519.

Ravid, T., and Hochstrasser, M. (2008). Diversity of degradation signals in the ubiquitin-proteasome system. Nat. Rev. Mol. Cell Biol. *9*, 679–690.

Rutkowski, D.T., Arnold, S.M., Miller, C.N., Wu, J., Li, J., Gunnison, K.M., Mori, K., Sadighi Akha, A.A., Raden, D., and Kaufman, R.J. (2006). Adaptation to ER stress is mediated by differential stabilities of pro-survival and pro-apoptotic mRNAs and proteins. PLoS Biol. *4*, e374.

Schwanhäusser, B., Busse, D., Li, N., Dittmar, G., Schuchhardt, J., Wolf, J., Chen, W., and Selbach, M. (2011). Global quantification of mammalian gene expression control. Nature *473*, 337–342.

Sharipo, A., Imreh, M., Leonchiks, A., Imreh, S., and Masucci, M.G. (1998). A minimal glycine-alanine repeat prevents the interaction of ubiquitinated I kappaB alpha with the proteasome: a new mechanism for selective inhibition of proteolysis. Nat. Med. *4*, 939–944.

Simister, P.C., Schaper, F., O'Reilly, N., McGowan, S., and Feller, S.M. (2011). Self-organization and regulation of intrinsically disordered proteins with folded N-termini. PLoS Biol. *9*, e1000591.

Stadtmueller, B.M., and Hill, C.P. (2011). Proteasome activators. Mol. Cell *41*, 8–19.

Takeuchi, J., Chen, H., and Coffino, P. (2007). Proteasome substrate degradation requires association plus extended peptide. EMBO J. *26*, 123–131.

Tian, L., Holmgren, R.A., and Matouschek, A. (2005). A conserved processing mechanism regulates the activity of transcription factors Cubitus interruptus and NF-kappaB. Nat. Struct. Mol. Biol. *12*, 1045–1053.

Tompa, P. (2003). Intrinsically unstructured proteins evolve by repeat expansion. BioEssays *25*, 847–855.

Tompa, P., Prilusky, J., Silman, I., and Sussman, J.L. (2008). Structural disorder serves as a weak signal for intracellular protein degradation. Proteins *71*, 903–909.

Tsvetkov, P., Asher, G., Paz, A., Reuven, N., Sussman, J.L., Silman, I., and Shaul, Y. (2008). Operational definition of intrinsically unstructured protein sequences based on susceptibility to the 20S proteasome. Proteins *70*, 1357–1366.

Tsvetkov, P., Reuven, N., and Shaul, Y. (2009). The nanny model for IDPs. Nat. Chem. Biol. *5*, 778–781.

van der Lee, R., Buljan, M., Lang, B., Weatheritt, R.J., Daughdrill, G.W., Dunker, A.K., Fuxreiter, M., Gough, J., Gsponer, J., Jones, D.T., et al. (2014). Classification of intrinsically disordered regions and proteins. Chem. Rev. *114*, 6589–6631.

van Ooijen, G., Dixon, L.E., Troein, C., and Millar, A.J. (2011). Proteasome function is required for biological timing throughout the twenty-four hour cycle. Curr. Biol. *21*, 869–875.

Varshavsky, A. (2012). The ubiquitin system, an immense realm. Annu. Rev. Biochem. *81*, 167–176.

Verhoef, L.G., Heinen, C., Selivanova, A., Halff, E.F., Salomons, F.A., and Dantuma, N.P. (2009). Minimal length requirement for proteasomal degradation of ubiquitin-dependent substrates. FASEB J. *23*, 123–133.

Ward, J.J., Sodhi, J.S., McGuffin, L.J., Buxton, B.F., and Jones, D.T. (2004). Prediction and functional analysis of native disorder in proteins from the three kingdoms of life. J. Mol. Biol. *337*, 635–645.

Wolfe, K.H., and Shields, D.C. (1997). Molecular evidence for an ancient duplication of the entire yeast genome. Nature *387*, 708–713.

Yang, C., Matro, J.C., Huntoon, K.M., Ye, D.Y., Huynh, T.T., Fliedner, S.M., Breza, J., Zhuang, Z., and Pacak, K. (2012). Missense mutations in the human SDHB gene increase protein degradation without altering intrinsic enzymatic function. FASEB J. *26*, 4506–4516.

Yen, H.C., Xu, Q., Chou, D.M., Zhao, Z., and Elledge, S.J. (2008). Global protein stability profiling in mammalian cells. Science *322*, 918–923.

Zhang, M., and Coffino, P. (2004). Repeat sequence of Epstein-Barr virus-encoded nuclear antigen 1 protein interrupts proteasome substrate processing. J. Biol. Chem. *279*, 8635–8641.

Zhao, M., Zhang, N.Y., Zurawel, A., Hansen, K.C., and Liu, C.W. (2010). Degradation of some polyubiquitinated proteins requires an intrinsic proteasomal binding element in the substrates. J. Biol. Chem. *285*, 4771–4780.

Cell Reports, Volume *8*

Supplemental Information

Intrinsically Disordered Segments Affect Protein Half-Life in the Cell and during Evolution

Robin van der Lee, Benjamin Lang, Kai Kruse, Jörg Gsponer, Natalia Sánchez de Groot, Martijn A. Huynen, Andreas Matouschek, Monika Fuxreiter, and M. Madan Babu

INVENTORY OF MAIN DISPLAY AND SUPPLEMENTAL DATA

Figure 1. The effects of terminal disordered segments on protein half-life

Figure S1A. Description of the yeast data used in our study
Figure S1B. The raw data show the non-linear relationship between the length of disordered segments and protein half-life
Figure S1C. The results for N-terminal disorder are independent of the average disorder scores
Figure S1D. The results are independent of the average disorder scores of the long N-terminal disordered segments
Figure S1E. C-terminal disorder lengths for proteins in different half-life groups do not differ significantly as a result of the experimental design used for half-life measurements
Figure S1F. Terminal disorder lengths for proteins in different half-life groups, divided along the median
Figure S1G. The results are independent of protein length
Figure S1H. The relationship between N-terminal disorder and half-life does not appear connected to the N-end rule

Table S1A. Compendium of datasets used in our study
Table S1B. Summary of boxplot parameters and significance estimates for the effects of disordered segments on protein half-life in yeast (DISOPRED2)
Table S1C. The results are independent of the method used to predict intrinsic protein disorder – IUPred
Table S1D. The results are independent of the method used to predict intrinsic protein disorder – PONDR VSL1
Table S1E. Disordered segments of different lengths influence protein half-life
Table S1F. Function enrichment analysis of proteins with long N-terminal disorder, long C-terminal disorder, and long N-terminal structure
Table S1G. The results are independent of protein length
Table S1H. The results are independent of protein abundance
Table S1I. The trends are not affected by cytoplasmic or nuclear localization
Table S1J. Removal of membrane proteins does not affect the observed trends
Table S1K. Using the Kolmogorov-Smirnov test to assess half-life differences yields equivalent results

Table S2 (separate Excel file). The primary data used in this study

Table 1. Conditional probabilities for intrinsically disordered segments and protein half-life

Table S3A. Extended conditional probabilities for N-terminal disorder and protein half-life
Table S3B. Extended conditional probabilities for internal disorder and protein half-life

Figure 2. The effects of internal disordered segments on protein half-life

Figure S2A. The results for internal disorder are independent of the average disorder scores of the proteins
Figure S2B. The results are independent of the average disorder scores of the internal disordered segments
Figure S2C. Proteins with multiple internal disordered segments generally have shorter half-lives than those with fewer
Figure S2D. Calculation of the minimal number of residues that may constitute an internal disordered segment that is directly cleavable by the 20S proteasome

Table S4A. The results for internal disorder are independent of the length of N-terminal disorder of the proteins
Table S4B. Function enrichment analysis of proteins with internal disordered segment(s)

Figure S3. The effects of terminal and internal disordered segments on protein half-life are independent of the overall degree of disorder

Figure S3A. The overall degree of disorder correlates with the length of disordered segments
Figure S3B. Proteins with various combinations of disordered segments were matched by their overall degree of disorder
Figure S3C. The combined effects of disordered segments on protein half-life are independent of the overall degree of disorder
Figure S3D. Statistics for the differences between half-life distributions are comparable when assessing only proteins with similar overall disorder degree or when assessing all proteins
Figure S3E. Proteins with and without disordered segments were paired based on their overall degree of disorder
Figure S3F. The individual effects of disordered segments on protein half-life are independent of the overall degree of disorder
Figure S3G. The number of unique proteins in pairs with and without disordered segments
Figure S3H. Statistics of half-life differences of paired proteins with and without disordered segments

Figure S3I. Proteins with different numbers of internal disordered segments were matched by their overall degree of disorder
Figure S3J. The effects of multiple internal disordered segments on protein half-life are independent of the overall degree of disorder
Figure S3K. Statistics for the differences between half-life distributions are comparable when assessing only proteins with similar overall disorder degree or when assessing all proteins

Figure 3. The effects of disordered segments on protein turnover in mouse and human

Table S5. Summary of boxplot parameters and significance estimates for the effects of disordered segments on protein turnover in mouse and human

Figure 4. Divergence in disordered segments during evolution can impact protein half-life

Figure S4A. Paralogous pairs with a negative half-life change have larger divergence in the length of N-terminal disorder
Figure S4B. Results of the paralogous protein analysis, without paralogs resulting from the ancestral yeast whole-genome duplication

Table S6A. Divergence of N-terminal or internal disordered segments is linked to protein half-life changes during evolution
Table S6B. Approximate permutation tests for N-terminal disorder in paralog pairs
Table S6C. Function enrichment analysis of paralog pairs that diverged in N-terminal or internal disordered segments

Table S7 (separate Excel file). Data on N-terminal disordered regions, internal disordered regions, and half-life for paralog pairs

Table 2. Conditional probabilities for intrinsically disordered segments and protein half-life in pairs of paralogs

Table S8A. Extended conditional probabilities for N-terminal disorder and protein half-life in pairs of paralogs
Table S8B. Extended conditional probabilities for internal disorder and protein half-life in pairs of paralogs

Figure 5. Concept describing the general relationship between the presence of long terminal or internal disordered segments and protein half-life

Supplemental Results

Results S1. The effects of disordered segments on half-life are independent of the overall disorder degree (extended)
Results S2. The results are independent of known degradation signals
Results S3. Disordered segments of proteins with short half-life lack enriched, uncharacterized sequence motifs that could explain the rapid degradation
Results S4. Paralogous pairs with a negative half-life change have larger divergence in the length of N-terminal disorder
Results S5. The reported trends are independent of confounding factors
Results S6. Highly stable proteins with undetermined or outlier half-life are generally less disordered
Results S7. The results are independent of protein length
Results S8. Degradation of membrane proteins
Results S9. The relationship between N-terminal disorder and half-life does not appear connected to the N-end rule

Supplemental Discussion

Discussion S1. Proteins without disordered segments can still be degraded quickly
Discussion S2. Disordered segments could influence the dynamics and regulation of signaling pathways (extended)

Supplemental Experimental Procedures

Supplemental References

SUPPLEMENTAL DATA

Figure S1

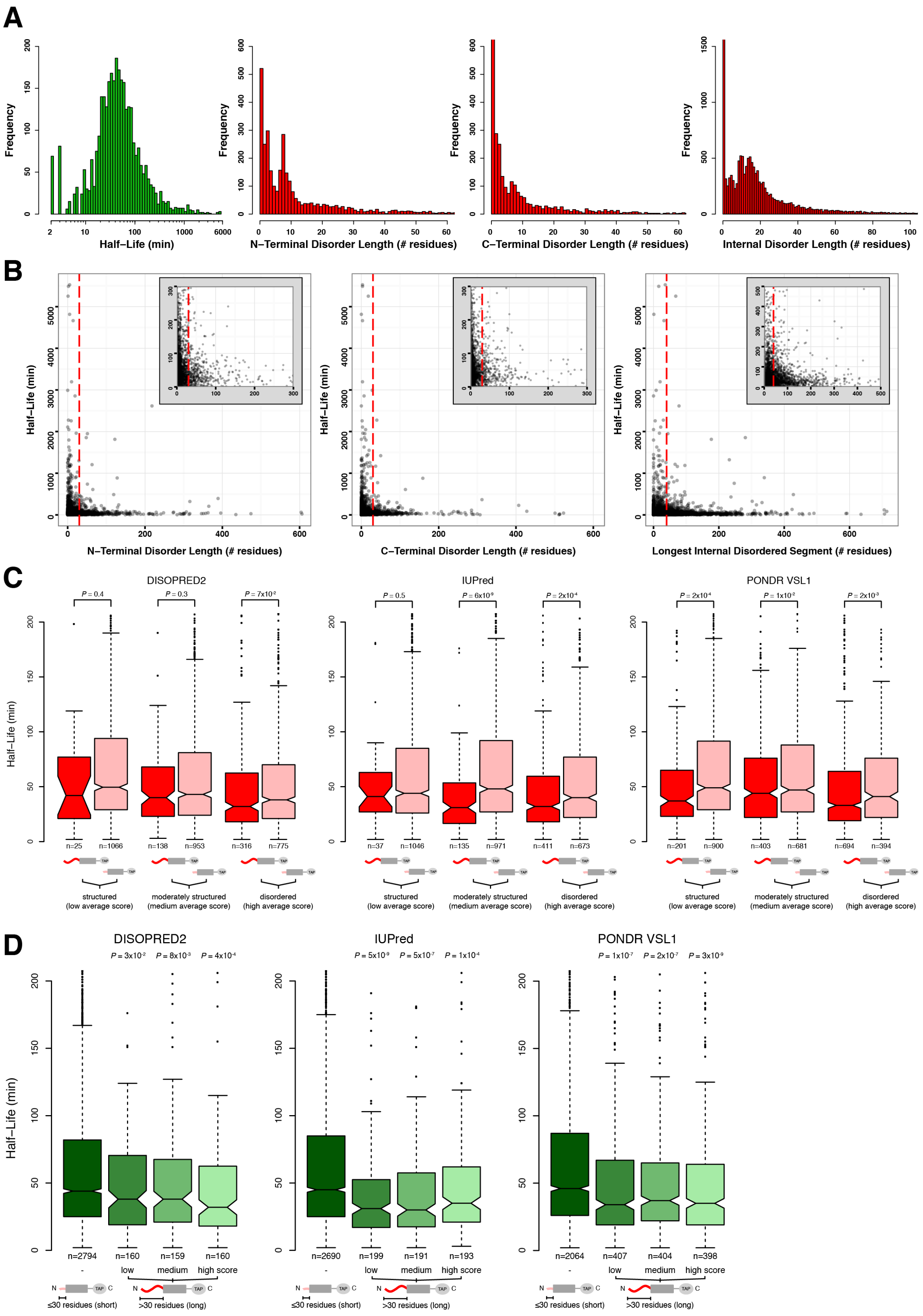

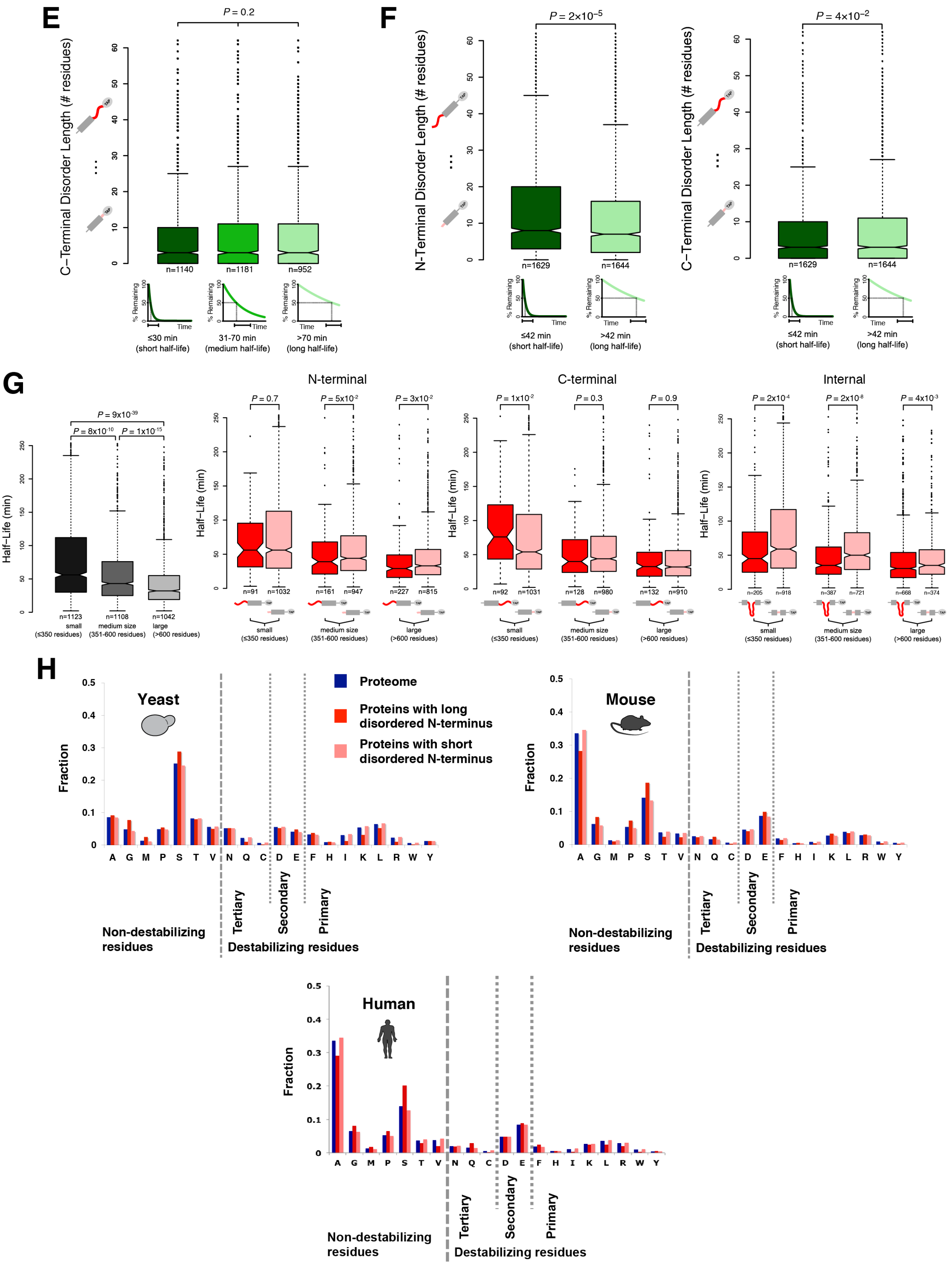
E
P = 0.2
C−Terminal Disorder Length (# residues)
n=1140
n=1181
n=952
% Remaining
Time
≤30 min
(short half-life)
31-70 min
(medium half-life)
>70 min
(long half-life)
F
P = 2x10−5
N-Terminal Disorder Length (# residues)
n=1629
n=1644
≤42 min
(short half-life)
>42 min
(long half-life)
P = 4x10−2
C−Terminal Disorder Length (# residues)
n=1629
n=1644
≤42 min
(short half-life)
>42 min
(long half-life)
G
P = 9x10-39
P = 8x10-10
P = 1x10-15
Half−Life (min)
n=1123
small
(≤350 residues)
n=1108
medium size
(351-600 residues)
n=1042
large
(>600 residues)
N-terminal
P = 0.7
P = 5x10-2
P = 3x10-2
n=91
n=1032
n=161
n=947
n=227
n=815
C-terminal
P = 1x10-2
P = 0.3
P = 0.9
n=92
n=1031
n=128
n=980
n=132
n=910
Internal
P = 2x10-4
P = 2x10-8
P = 4x10-3
n=205
n=918
n=387
n=721
n=668
n=374
small
(≤350 residues)
medium size
(351-600 residues)
large
(>600 residues)
H
Yeast
Mouse
Human
Proteome
Proteins with long disordered N-terminus
Proteins with short disordered N-terminus
Fraction
A G M P S T V N Q C D E F H I K L R W Y
Tertiary
Secondary
Primary
Non-destabilizing residues
Destabilizing residues

Figure S1. Supplemental data for the effects of terminal disordered segments on protein half-life, Related to Figure 1

Boxplots of the distribution of half-life values and lengths of disordered segments for the different groups of proteins in *S. cerevisiae*. For further information about the plot content see **Figure 1** in the main text.

(A) Description of the yeast data used in our study

The histograms show, from left to right, the distributions of protein half-life (log-scale), N-terminal disorder length, C-terminal disorder length, and internal disorder length. For clarity the axes were limited to 60 (N- and C-terminal disorder length) and 100 residues (internal disorder length); several longer disordered segments do occur, but only in low frequencies. Note that multiple internal disordered regions may occur in one protein, which increases the number of data points in the internal disorder panel.

(B) The raw data show the non-linear relationship between the length of disordered segments and protein half-life

Scatterplots of protein half-life versus N-terminal disorder length (left), C-terminal disorder length (middle), and the length of the longest internal disordered segment (right). Insets show the same plots, zoomed in on cluttered areas. Red dashed lines correspond to the cutoffs used for grouping the proteins into those with long and those with short disordered segments: 30 residues for both N- and C-terminal disorder, and 40 residues for internal disorder. In the N-terminal and C-terminal disorder plots, one data point above 600 residues in length was not shown to improve visualization. The same is true for one data point above 750 residues in length for the internal disorder plot.

(C) The results for N-terminal disorder are independent of the average disorder scores

Proteins were divided into tertiles according to the average disorder score for the whole protein as reported by the three disorder predictors: DISOPRED2 (left), IUPred (middle) and PONDR VSL1 (right). *P* values (Mann-Whitney *U* test) shown compare the disordered group to its non-disordered counterpart within the same score tertile.

(D) The results are independent of the average disorder scores of the long N-terminal disordered segments

Proteins were divided into tertiles according to the average disorder score for the long N-terminal disordered segments as reported by the three disorder predictors: DISOPRED2 (left), IUPred (middle) and PONDR VSL1 (right). The left-most boxplot in each panel (dark green) contains proteins without the disorder type of interest, i.e. proteins without a long disordered N-terminus. *P* values (Mann-Whitney *U* test) shown compare each score tertile to the non-disordered group.

(E) C-terminal disorder lengths for proteins in different half-life groups do not differ significantly as a result of the experimental design used for half-life measurements

The nature of the experimental design used to measure protein half-life necessarily ensured that all proteins have identical C-termini (i.e. the TAP-tag) (Belle et al., 2006). Therefore, as expected, the distributions of C-terminal disorder lengths, which were predicted based on the sequence of the untagged genomic proteins, are not significantly different ($P = 0.2$, Kruskal-Wallis test) between different half-life groups (indicated with exponential degradation curves; from short half-life, dark green, to long half-life, light green). Outliers with C-terminal disorder length >60 residues are not shown to improve visualization.

(F) Terminal disorder lengths for proteins in different half-life groups, divided along the median

Plots are shown for N-terminal disorder length (left) and C-terminal disorder length (right). Proteins were divided into two half-life groups: (i) shorter than or equal to the median (≤42 minutes, short half-life), and (ii) longer than the median (>42 minutes, long half-life). Division of the proteins into two half-life groups, instead of three (**Figures 1E** and **S1E**), does not affect the conclusions. Outliers with a length of >60 residues are not shown to improve visualization.

(G) The results are independent of protein length

Proteins were classified based on, from left to right: overall length (i.e. size), both the length of N-terminal disorder and overall length, both the length of C-terminal disorder and overall length, and both the presence of an internal disordered segment and overall length. Outliers with half-life >250 minutes are not shown to improve visualization.

(H) The relationship between N-terminal disorder and half-life does not appear connected to the N-end rule

Frequency distributions of N-terminal residues next to the initiator methionines in yeast (top-left), mouse (top-right) and human (bottom) for the entire proteome (blue), proteins with a long disordered N-terminus (dark red), and proteins with a short disordered N-terminus (light red). The N-end rule defines non-destabilizing and destabilizing (primary, secondary, and tertiary) residues. See also **Supplemental Results S9**.

Figure S2

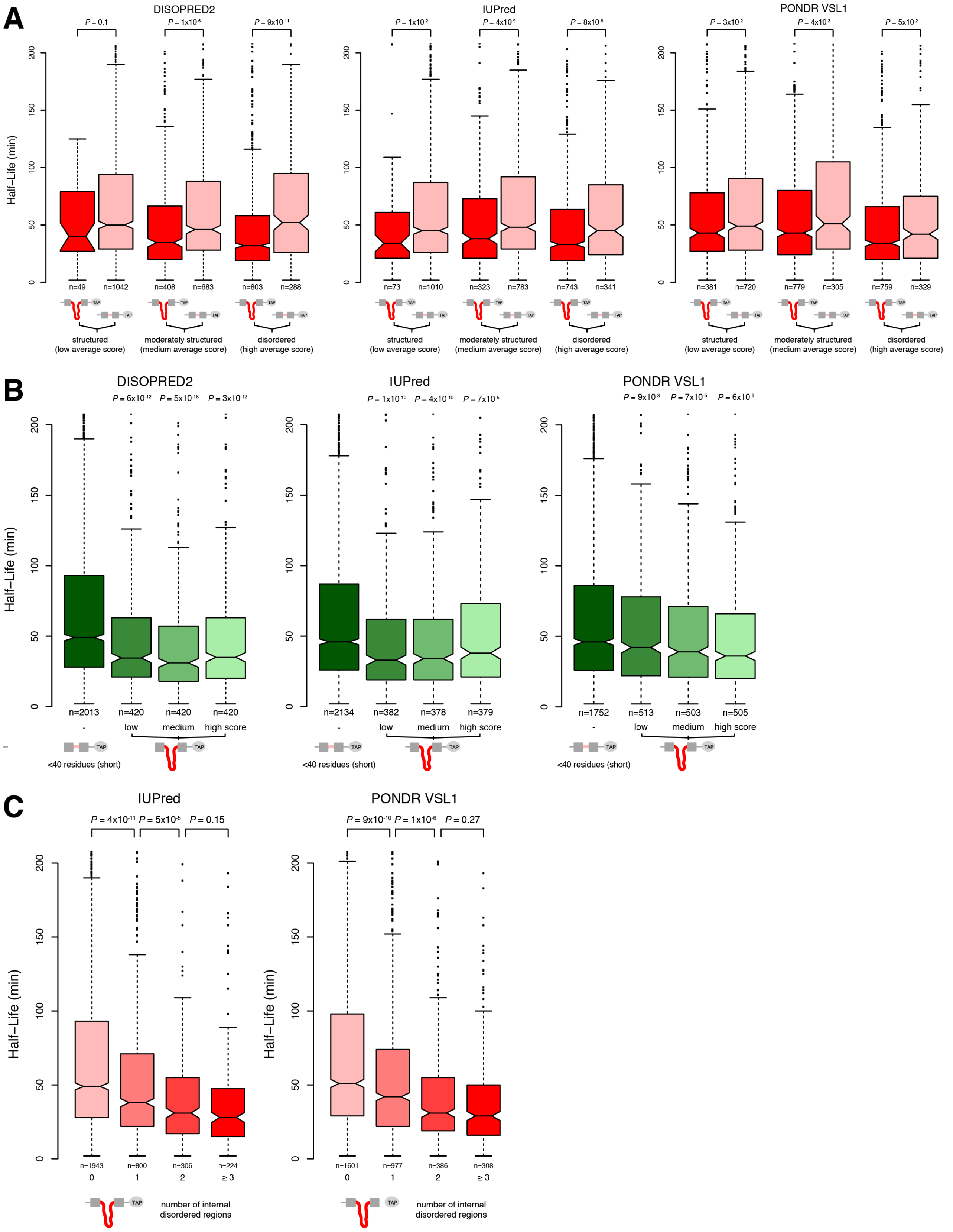
A
DISOPRED2
IUPred
PONDR VSL1
Half-Life (min)
structured (low average score)
moderately structured (medium average score)
disordered (high average score)
B
DISOPRED2
IUPred
PONDR VSL1
<40 residues (short)
low
medium
high score
C
IUPred
PONDR VSL1
number of internal disordered regions

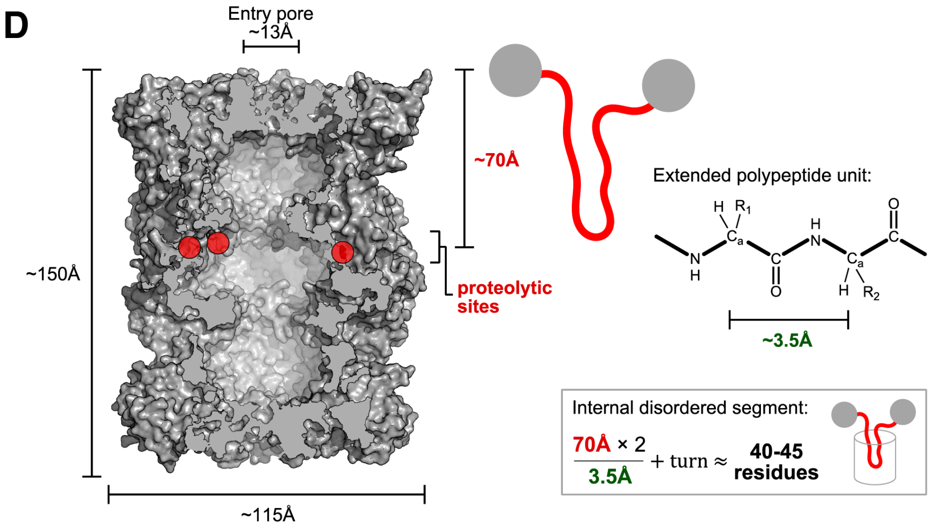

Figure S2. Supplemental data for the effects of internal disordered segments on protein half-life, Related to Figure 2

Boxplots of the distribution of half-life values and lengths of disordered segments for the different groups of proteins in *S. cerevisiae*. For further information about the plot content see **Figures 1** and **2** in the main text.

(A) The results for internal disorder are independent of the average disorder scores of the proteins

Proteins were divided into tertiles according to the average disorder score for the whole protein as reported by the three disorder predictors: DISOPRED2 (left), IUPred (middle) and PONDR VSL1 (right). The *P* values shown compare the disordered group to its non-disordered counterpart within the same score tertile, and were calculated using the Mann-Whitney *U* test.

(B) The results are independent of the average disorder scores of the internal disordered segments

Proteins were divided into tertiles according to the average disorder score for the internal disordered segment as reported by the three disorder predictors: DISOPRED2 (left), IUPred (middle) and PONDR VSL1 (right). The left-most boxplot in each panel (dark green) contains proteins without the disorder type of interest, i.e. proteins without an internal disordered segment. The *P* values shown compare each score tertile to the non-disordered group, using the Mann-Whitney *U* test.

(C) Proteins with multiple internal disordered segments generally have shorter half-lives than those with fewer

Proteins were classified based on the number of internal disordered segments (defined as continuous stretches of ≥40 disordered residues; from zero, to three or more, shown below each boxplot) as reported by disorder predictors IUPred (left) and PONDR VSL1 (right). The corresponding DISOPRED2 panel can be found in **Figure 2C** in the main text.

(D) Calculation of the minimal number of residues that may constitute an internal disordered segment that is directly cleavable by the 20S proteasome

The average distance from the 20S entry pore to the three proteolytic sites inside the proteasome is ~70Å. An extended polypeptide unit spans ~3.5Å. Therefore a conservative estimate of the minimum number of internal disordered residues that can be cleaved directly by the 20S proteasome is ~40-45 amino acids. Distance measurements were done on the crystal structure of the 20S yeast proteasome (PDB ID: 1RYP (Groll et al., 1997)) using the PyMOL Molecular Graphics System, Version 1.3 (Schrodinger, 2010).

Figure S3

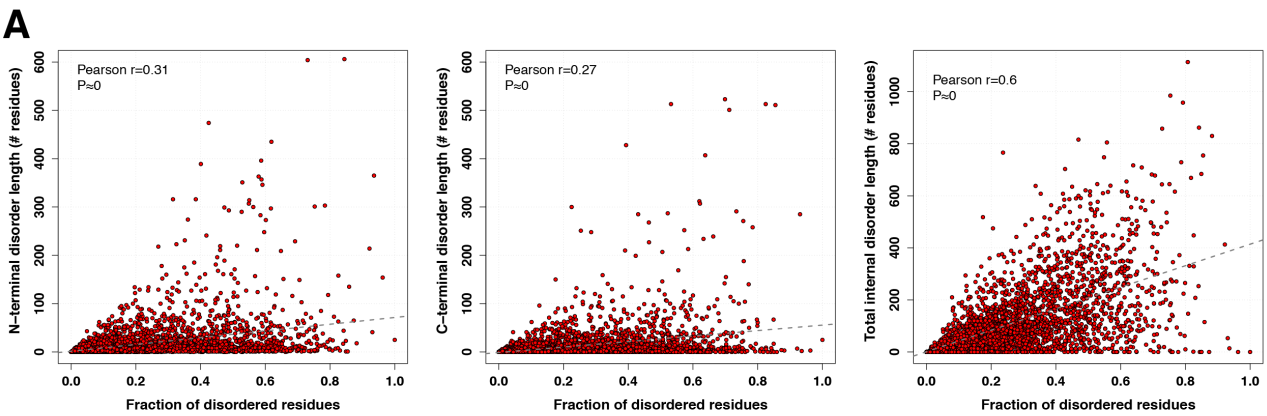

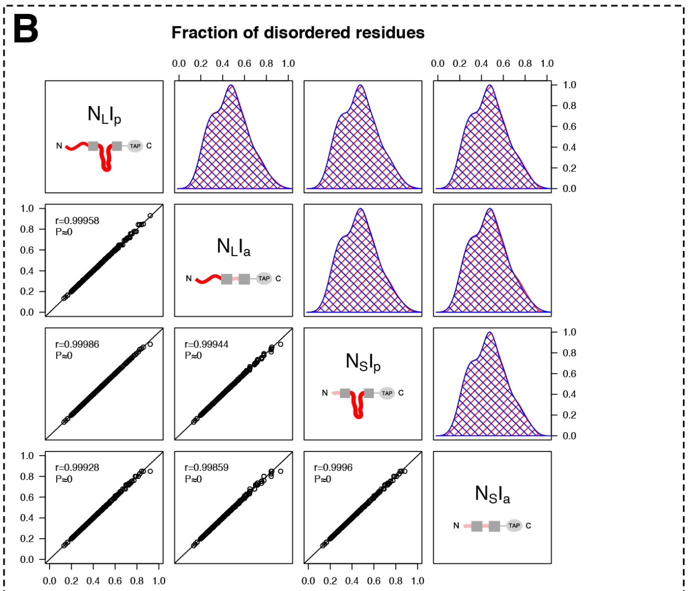

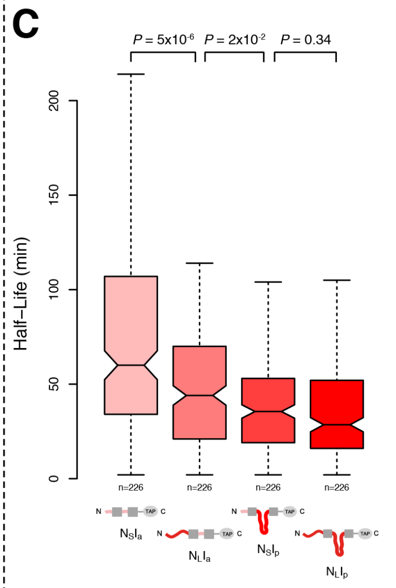

D

Proteins matched by overall disorder degree			
N_LI_p	7	15.5	31.5
0.34	N_SI_p	8.5	24.5
4e-3	2e-2	N_LI_a	16
8e-10	1e-8	5e-6	N_SI_a

Effect size / P value

All proteins			
N_LI_p	6.5	14	22
2e-2	N_SI_p	8	15
6e-5	4e-3	N_LI_a	7
3e-15	2e-24	3e-3	N_SI_a

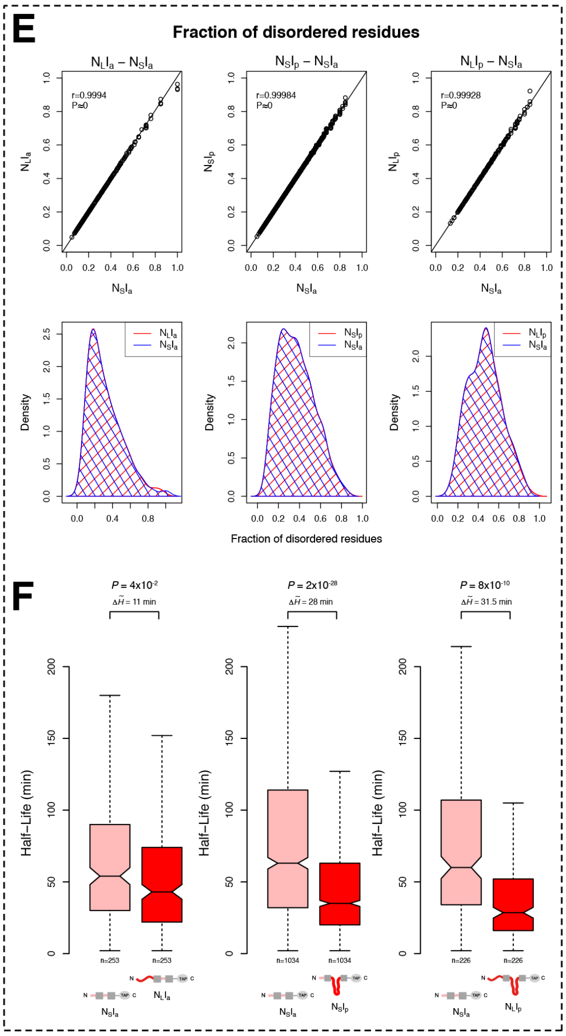

G

Pairing	Pairs	Unique N_SI_a proteins	Maximal occurrences of non-unique N_SI_a proteins
N_LI_a - N_SI_a	253	200 (79%)	6
N_SI_p - N_SI_a	1034	402 (39%)	20
N_LI_p - N_SI_a	226	125 (55%)	9

H

Pairing	Pairs with unique proteins	Effect size (N=1000)					*P* value (N=1000)				
		Original	*Median*	*Mean*	*Min*	*Max*	*Original*	*Median*	*Mean*	*Min*	*Max*
N_LI_a - N_SI_a	200	11	11	11.23	8.5	13.5	0.035	0.037	0.041	0.0060	0.14
N_SI_p - N_SI_a	402	28	18	18.21	14	22	1.6e-28	1.1e-7	9.8e-7	3.0e-11	6.0e-5
N_LI_p - N_SI_a	125	31.5	34	33.77	27	37	7.7e-10	9.6e-6	4e-5	2.9e-8	3.0e-3

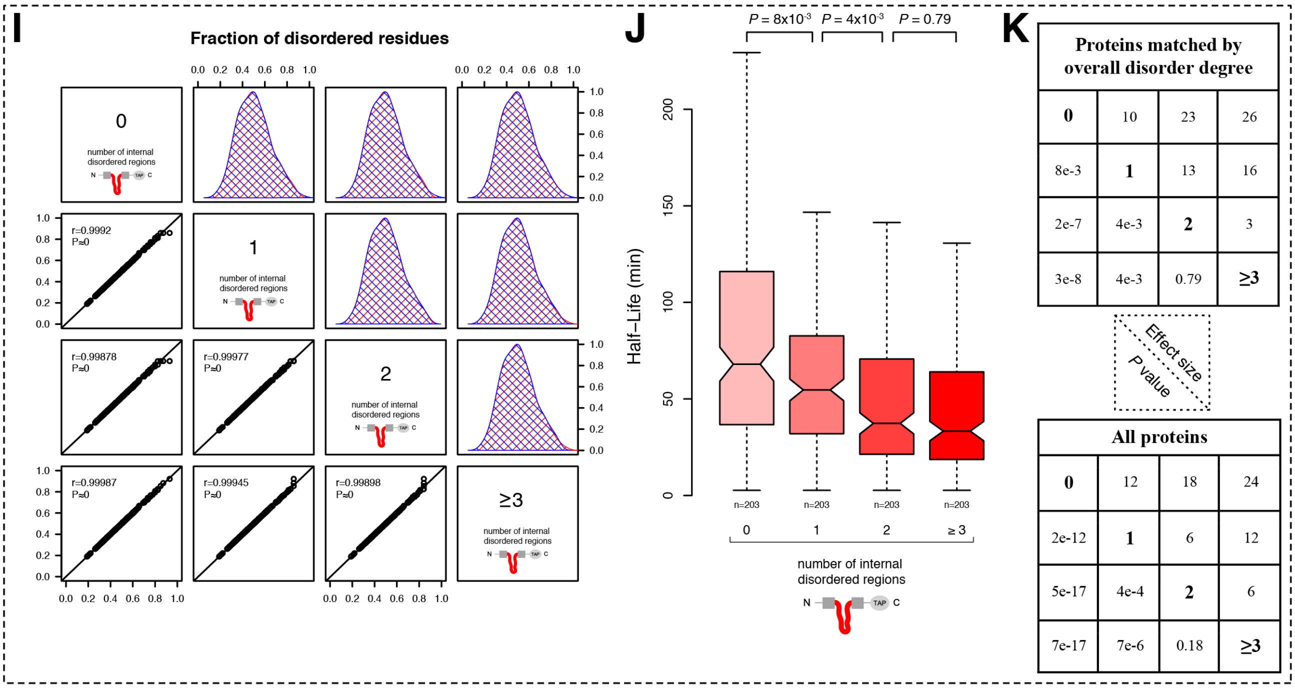

Figure S3. Supplemental data showing that the effects of terminal and internal disordered segments on protein half-life are independent of the overall degree of disorder, Related to Figures 1 and 2

For further information about the plot content see **Figures 1** and **2** in the main text.

(A) The overall degree of disorder correlates with the length of disordered segments

Correlations between N-terminal disorder length (left), C-terminal disorder length (middle), and total internal disorder length (right) and the fraction of disordered residues (i.e. overall degree of disorder) in a protein. Dotted gray lines represent the linear fits of y ~ x. Pearson r statistics and *P* values are reported within each plot. In the N-terminal (left) and C-terminal (middle) scatterplots, one data point above 600 residues in length was not shown to improve visualization.

(B) Proteins with various combinations of disordered segments were matched by their overall degree of disorder

Scatterplots (bottom-left triangle) and polygon overlays of the kernel density estimates (top-right triangle) of the overall disorder degree of pairs of proteins with various combinations of disordered segments (indicated in the diagonal squares). Pearson r statistics and *P* values are reported in the scatterplots. Disorder degree density estimates were calculated using a Gaussian kernel with a smoothing bandwidth given by Silverman's rule of thumb (~0.05 for all combinations) and were normalized to 1. The blue and red polygons correspond to the densities of the protein class to the left and to the bottom side of the plot, respectively. Correlations and density overlays indicate almost perfect linear correlation between the overall disorder degree of pairs of proteins from different classes, demonstrating that the matched proteins indeed have almost identical overall degrees of disorder.

(C) The combined effects of disordered segments on protein half-life are independent of the overall degree of disorder

Boxplots of the distribution of half-life values for proteins with various combinations of disordered segments in *S. cerevisiae*. Proteins were classified into those that have a long disordered N-terminus (N_LI_a), those that have an internal disordered segment (N_SI_p), those that have both a terminal and an internal disordered segment (N_LI_p), and those that have neither (N_SI_a). For each protein with a long N-terminal disordered segment that also has a long internal disordered segments (N_LI_p, 226 cases, the minority class), one protein from each of the other classes that is closest to the N_LI_p protein in terms of the fraction of disordered residues was selected. *P* values were calculated using the Wilcoxon Signed-Rank test, which is a non-parametric test for assessing difference between two paired samples. *P* values and median differences are also given in the top panel of **Figure S3D**.

(D) Statistics for the differences between half-life distributions are comparable when assessing only proteins with similar overall disorder degree or when assessing all proteins

P values (bottom-left triangles) and absolute differences in half-life medians (effect size; top-right triangles) between half-life distributions of groups of proteins with various combinations of disordered segments. The top panel shows statistics based on matched proteins with similar overall degrees of disorder. Here, *P* values were calculated using the Wilcoxon Signed-Rank test, which is a non-parametric test for assessing difference between two paired samples. The bottom panel shows statistics based on all proteins. Here, *P* values were calculated using the Mann-Whitney *U* test.

(E) Proteins with and without disordered segments were paired based on their overall degree of disorder

Scatterplots (top) and polygon overlays of the kernel density estimates (bottom) of the overall disorder degree of protein pairs. Each N_SI_a protein was paired with a protein from each of the other classes based on the overall degree of disorder, making N_LI_a - N_SI_a (left), N_SI_p - N_SI_a (middle), and N_LI_p - N_SI_a pairs (right). Proteins with disordered segments were selected based on the absolute difference with the overall disorder degree of the N_SI_a protein. Pearson r statistics and *P*

values are reported in the scatterplots. Disorder degree density estimates were calculated using a Gaussian kernel with a smoothing bandwidth given by Silverman's rule of thumb (~0.056 for N_LI_a - N_SI_a; ~0.038 for N_SI_p - N_SI_a; ~0.050 for N_LI_p - N_SI_a). Correlations and density overlays indicate almost perfect linear correlation between the overall disorder degree of the various pairings, demonstrating that paired proteins selected using the above described method indeed have almost identical overall degrees of disorder.

(F) The individual effects of disordered segments on protein half-life are independent of the overall degree of disorder

Boxplots of the distribution of half-life values for proteins with and without disordered segments, paired on similar overall degree of disorder. Proteins were classified into those that have a long disordered N-terminus (N_LI_a), those that have an internal disordered segment (N_SI_p), those that have both a terminal and an internal disordered segment (N_LI_p), and those that have neither (N_SI_a). Each N_SI_a was paired with a protein from each of the other classes based on the overall degree of disorder, making N_LI_a - N_SI_a (left), N_SI_p - N_SI_a (middle), and N_LI_p - N_SI_a pairs (right). Reported *P* values were calculated using the Wilcoxon Signed-Rank test, which is a non-parametric test for assessing differences between two paired samples. $\Delta\tilde{H}$ reports the difference between the half-life medians of the compared groups.

(G) The number of unique proteins in pairs with and without disordered segments

The approach for pairing proteins with disordered segments to proteins with no disordered segments (N_SI_a) based on overall disorder degree similarity can result in an N_SI_a protein being sampled more than once. 'Pairs' shows the total number of pairs and equals to the number of proteins from the class that has least proteins (in all pairings this is the class with disordered segments). 'Unique N_SI_a proteins' shows the number of unique proteins without disordered segments (N_SI_a); the difference with the 'Pairs' column indicates the number of pairs containing N_SI_a proteins that have been sampled more than once (i.e. in multiple pairs). 'Maximal occurrences of non-unique N_SI_a proteins' shows how often the most abundantly sampled N_SI_a protein occurs and is an indication of the number of duplicate data points resulting from the pairing based on disorder degree similarity.

(H) Statistics of half-life differences of paired proteins with and without disordered segments

Protein pairs containing a protein without disordered segments (N_SI_a) that occurs in multiple pairs were randomly removed until individual proteins occur only once. The procedure was repeated 1000 times to assess the robustness of the results. 'Pairs with unique proteins' shows the number of proteins pairs that remain after removing pairs containing non-unique N_SI_a proteins. Effect sizes (difference in median half-life) and *P* values (Wilcoxon Signed-Rank test) were calculated for the differences between half-life distributions of proteins with and without disordered segments (comparing N_LI_a with N_SI_a, top row; N_SI_p with N_SI_a, middle row; and N_LI_p with N_SI_a, bottom row). 'Original' columns show the effect sizes and *P* values for the full paired dataset (i.e. including non-unique N_SI_a proteins; **Figure S3F**). 'Median', 'Mean', 'Min', and 'Max' describe the effect size and *P* value distributions resulting from the 1000 randomizations.

(I) Proteins with different numbers of internal disordered segments were matched by their overall degree of disorder

Scatterplots (bottom-left triangle) and polygon overlays of the kernel density estimates (top-right triangle) of the overall disorder degree of pairs of proteins with different numbers of internal disordered segments (from zero to three or more, indicated in the diagonal squares). Pearson r statistics and *P* values are reported in the scatterplots. Disorder degree density estimates were calculated using a Gaussian kernel with a smoothing bandwidth given by Silverman's rule of thumb (~0.045 for all combinations) and were normalized to 1. The blue and red polygons correspond to the densities of the protein class to the left and to the bottom side of the plot, respectively. Correlations and density overlays indicate almost perfect linear correlation between the overall disorder degree of pairs of proteins from different classes, demonstrating that the matched proteins indeed have almost identical overall degree of disorder.

(J) The effects of multiple internal disordered segments on protein half-life are independent of the overall degree of disorder

Boxplots of the distribution of half-life values for proteins with different numbers of internal disordered segments (from zero to three or more) in *S. cerevisiae*. For each protein with three or more internal disordered segments (203 proteins, the minority class), one protein from each of the other classes that is closest in terms of the fraction of disordered residues was selected. *P* values were calculated using the Wilcoxon Signed-Rank test, which is a non-parametric test for assessing difference between two paired samples. *P* values and median differences are also given in the top panel of **Figure S3K**.

(K) Statistics for the differences between half-life distributions are comparable when assessing only proteins with similar overall disorder degree or when assessing all proteins

P values (bottom-left triangles) and absolute differences in half-life medians (effect size; top-right triangles) between half-life distributions of groups of proteins with different numbers of internal disordered segments. The top panel shows statistics based on matched proteins with similar overall degrees of disorder. Here, *P* values were calculated using the Wilcoxon Signed-Rank test, which is a non-parametric test for assessing difference between two paired samples. The bottom panel shows statistics based on all proteins. Here, *P* values were calculated using the Mann-Whitney *U* test.

Figure S4

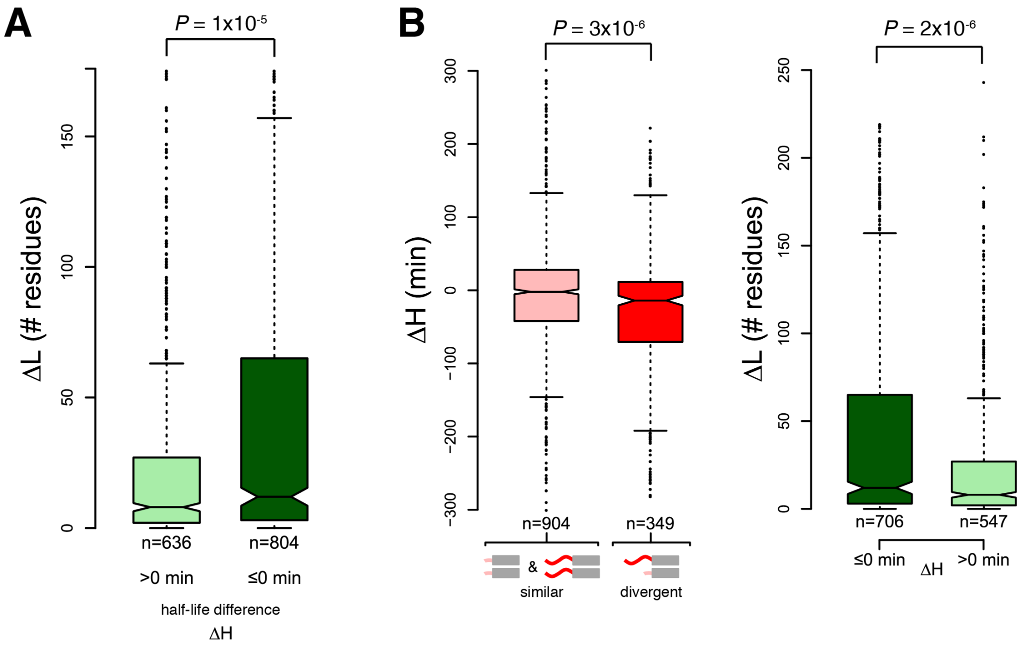

Figure S4. Supplemental data for the effects of N-terminal disorder on protein half-life in paralog pairs, Related to Figure 4

(A) Paralogous pairs with a negative half-life change have larger divergence in the length of N-terminal disorder

Boxplots of the distribution of N-terminal disorder difference values ΔL within paralog pairs (defined as $\Delta L = L_1 - L_2$; $L_1 \geq L_2$), grouped by their half-life difference ΔH. Left: ΔH >0 min. Right: ΔH ≤0 min. The length of N-terminal disordered segments changes more in paralog pairs where divergence in disorder coincides with half-life decrease (negative half-life change); see also **Supplemental Results S4**. Outliers with ΔL > 175 are not shown to improve visualization. For further information about the plot content see **Figure 4** in the main text.

(B) Results of the paralogous protein analysis, without paralogs resulting from the ancestral yeast whole-genome duplication

(**left**) Boxplots of the distribution of half-life difference values ΔH. Paralog pairs are grouped by the difference in their N-terminal disorder length: (i) either both paralogs have a short (≤30 residues) or both have a long (>30 residues) disordered N-terminus (light red, left boxplot of the two), and (ii) one paralog has a short, the other a long disordered N-terminus (dark red, right boxplot of the two). (**right**) Boxplots of the distribution of N-terminal disorder difference values ΔL. Paralog pairs have been grouped by their half-life difference ΔH: ΔH ≤ 0 min (dark green, left boxplot of the two) and ΔH > 0 min (light green, right boxplot of the two). Outliers with ΔH > 300 (left) and ΔL > 250 (right) are not shown to improve visualization. For further information about the plot content see **Figure 4** in the main text. The number of paralogous protein pairs resulting from the ancestral yeast whole genome duplication alone and for which we have half-life and disorder data was too small to make meaningful comparisons of their ΔH and ΔL. Therefore, an analysis of whole-genome paralogs alone could not be performed.

Supplemental Tables

Table S1, Related to Figures 1 and 2

Table S1A. Compendium of datasets used in our study

**Type of information** [source]	**Description of the method used to obtain the data**
**Disorder predictions** (Dosztanyi et al., 2005; Obradovic et al., 2005; Ward et al., 2004)	The disorder status of every residue in the yeast, mouse, and human proteomes was inferred using the DISOPRED2, IUPRED, and PONDR VLS1 predictors.
**Protein half-life**	
Yeast (Belle et al., 2006)	*In vivo* protein half-lives were determined by first inhibiting protein synthesis with the antibiotic cycloheximide and then monitoring the abundance of each C-terminally TAP-tagged protein in the yeast genome by quantitative Western blotting at three time points.
Mouse (Schwanhausser et al., 2011)	*In vivo* protein half-lives in NIH3T3 mouse fibroblasts were derived from the ratio between the heavy and light peptides, measured using mass spectrometry at different time points after the transfer of cells from light to heavy medium.
Human (Kristensen et al., 2013)	*In vivo* relative degradation rates in human THP-1 myelomonocytic leukemia cells, under conditions that stimulate cell proliferation, were determined using a similar SILAC MS approach as in mouse, above.
**Paralogs** (Altschul et al., 1990; Wolfe and Shields, 1997)	A list of paralogous proteins in yeast was obtained from an all-against-all protein sequence comparison followed by clustering using the program BLASTClust. This list was supplemented with more divergent pairs of paralogs that arose from the whole genome duplication event in yeast.
**Degradation signals**	
Ubiquitination (Uniprot-Consortium, 2011)	Experimentally determined ubiquitination sites were obtained from UniProtKB/Swiss-Prot.
KEN, Destruction box motifs (Liu et al., 2012; Pfleger and Kirschner, 2000)	KEN box and destruction box motifs were predicted using GPS-ARM 1.0.
PEST sequences (Rice et al., 2000; Rogers et al., 1986)	PEST regions were predicted using epestfind with default parameters, as included in EMBOSS 6.5.7.
N-end rule (Bachmair et al., 1986)	Frequencies of amino acids in the second N-terminal residue (after removal of the initiator methionine) were calculated for the yeast, mouse and human proteomes.
**Sequence motifs** (Davey et al., 2010; Neduva et al., 2005)	SLiMFinder and DiliMot were used to analyze the amino acid sequences of disordered segments of proteins with short half-life for shared sequence patterns that potentially facilitate rapid degradation.
**Protein abundance** (Newman et al., 2006)	Protein levels during log-phase growth of yeast were obtained by flow cytometry measurements of GFP-tagged strains.
**Subcellular localization** (Christie et al., 2004)	Subcellular localization information (cytoplasm and nucleus) was obtained from the Saccharomyces Genome Database.
**Membrane proteins** (Kim et al., 2006; Melen et al., 2003; Osterberg et al., 2006)	Membrane protein topologies were previously determined by measurements of the location of the C-terminus (cytosolic or extracellular, determined using tagged constructs), which was used to constrain topology predictions by TMHMM.

Median values ($\tilde{x}$) ± confidence interval (C.I. = $1.58(IQR/\sqrt{n})$, where IQR is the interquartile range and 'n' the group sample size) are reported for different groups of proteins. The IQR is calculated as the difference between the data points at the ×0.75 and ×0.25 quartiles. *P* values for the differences in the distributions of half-life values between the different groups ('long' versus 'short' and 'yes' versus 'no') were calculated using the Mann-Whitney *U* test and are reported in parentheses.

Table S1B. Summary of boxplot parameters and significance estimates for the effects of disordered segments on protein half-life in yeast (DISOPRED2)
Related to **Figures 1B**, **1C** and **2A**.

**Property**		$\tilde{x}$ **± C.I. (Half-Life [min])**	**n**
**N-terminal disorder length**	Long (>30 residues)	35 ± 3.4	479
	Short (≤30 residues)	44 ± 1.7 ($5x10^{-6}$)	2794
**C-terminal disorder length**	Long (>30 residues)	43.5 ± 4.7	352
	Short (≤30 residues)	43 ± 1.7 (0.99)	2921
**Internal disordered segment**	yes (≥40 residues)	34 ± 1.9	1260
	no (<40 residues)	49 ± 2.3 ($3x10^{-29}$)	2013

Table S1C. The results are independent of the method used to predict intrinsic protein disorder – IUPred

**Property**		$\tilde{x}$ **± C.I. (Half-Life [min])**	**n**
**N-terminal disorder length**	Long (>30 residues)	32 ± 2.5	694
	Short (≤30 residues)	46 ± 1.9 ($9x10^{-18}$)	2579
**C-terminal disorder length**	Long (>30 residues)	40 ± 3.4	642
	Short (≤30 residues)	43 ± 1.8 (0.13)	2631
**Internal disordered segment**	yes (≥40 residues)	34 ± 1.9	1330
	no (<40 residues)	49 ± 2.3 ($4x10^{-29}$)	1943

Table S1D. The results are independent of the method used to predict intrinsic protein disorder – PONDR VSL1

**Property**		$\tilde{x}$ **± C.I. (Half-Life [min])**	**n**
**N-terminal disorder length**	Long (>30 residues)	36 ± 2.4	875
	Short (≤30 residues)	45 ± 1.9 ($6x10^{-12}$)	2397
**C-terminal disorder length**	Long (>30 residues)	39 ± 3.1	809
	Short (≤30 residues)	44 ± 1.8 ($1x10^{-2}$)	2463
**Internal disordered segment**	yes (≥40 residues)	36 ± 1.7	1671
	no (<40 residues)	51 ± 2.7 ($3x10^{-27}$)	1601

Table S1E. Disordered segments of different lengths influence protein half-life

Length cutoffs of 30 terminal disordered residues and 40 internal disordered residues produce the largest differences between the half-lives of proteins with and without disordered segments, but shorter and longer segments also contribute to shorter protein half-life.

Property		$\tilde{x}$ ± C.I. (Half-Life [min])	n
**N-terminal disorder length in # residues**			
cutoff = 20	Long (>20 residues)	37 ± 3.0	739
	Short (≤20 residues)	44 ± 1.8 ($3x10^{-4}$)	2534
cutoff = 25	Long (>25 residues)	36 ± 3.2	588
	Short (≤25 residues)	44 ± 1.7 ($1x10^{-4}$)	2685
cutoff = 30 (standard)	Long (>30 residues)	35 ± 3.4	479
	Short (≤30 residues)	44 ± 1.7 ($5x10^{-6}$)	2794
cutoff = 35	Long (>35 residues)	34 ± 3.4	421
	Short (≤35 residues)	44 ± 1.7 ($2x10^{-6}$)	2852
cutoff = 40	Long (>40 residues)	33.5 ± 3.6	366
	Short (≤40 residues)	44 ± 1.7 ($1x10^{-5}$)	2907
**C-terminal disorder length in # residues**			
cutoff = 20	Long (>20 residues)	44 ± 3.8	498
	Short (≤20 residues)	42 ± 1.7 (0.44)	2775
cutoff = 25	Long (>25 residues)	44 ± 4.3	401
	Short (≤25 residues)	42 ± 1.7 (0.63)	2872
cutoff = 30 (standard)	Long (>30 residues)	43.5 ± 4.7	352
	Short (≤30 residues)	43 ± 1.7 (0.99)	2921
cutoff = 35	Long (>35 residues)	43 ± 5.3	292
	Short (≤35 residues)	43 ± 1.6 (0.98)	2981
cutoff = 40	Long (>40 residues)	43 ± 5.7	238
	Short (≤40 residues)	43 ± 1.6 (0.71)	3035
**Internal disordered segment in # residues**			
cutoff = 30	yes (≥30 residues)	36 ± 1.8	1573
	no (<30 residues)	50 ± 2.5 ($3x10^{-24}$)	1700
cutoff = 35	yes (≥35 residues)	35 ± 1.8	1428
	no (<35 residues)	49 ± 2.4 ($1x10^{-24}$)	1845
cutoff = 40 (standard)	yes (≥40 residues)	34 ± 1.9	1260
	no (<40 residues)	49 ± 2.3 ($3x10^{-29}$)	2013
cutoff = 45	yes (≥45 residues)	34 ± 1.9	1128
	no (<45 residues)	48 ± 2.1 ($4x10^{-25}$)	2145
cutoff = 50	yes (≥50 residues)	33 ± 2.1	1014
	no (<50 residues)	47 ± 2 ($1x10^{-23}$)	2259

Table S1F. Function enrichment analysis of proteins with long N-terminal disorder, long C-terminal disorder, and long N-terminal structure

Function enrichment was determined using the functional annotation tool of the DAVID suite version 6.7 (Huang da et al., 2009a, b), with default settings and a false discovery rate of 0.01. Entries from the Functional Annotation Chart are shown for each protein group, excluding UniProt sequence features. In cases where multiple entries form a cluster at medium stringency according to the Functional Annotation Clustering view, only the entry with the lowest q-value is shown for conciseness. Background: all yeast proteins.

Proteins with long N-terminal disorder (>30 aa) (n=479)

Source	Term	Term type or identifier	Percentage	Enrichment	q-value
SP_PIR_KEYWORDS	phosphoprotein	-	74.7%	1.84	1.1e-52
SP_PIR_KEYWORDS	serine/threonine-protein kinase	-	9.0%	4.78	1.9e-15
SP_PIR_KEYWORDS	nucleus	-	43.0%	1.70	2.1e-15
SP_PIR_KEYWORDS	transcription regulation	-	17.1%	2.33	1.9e-10
GOTERM_BP_FAT	regulation of transcription	GO:0045449	21.7%	1.79	8.3e-07
INTERPRO	AGC-kinase, C-terminal	IPR000961	1.9%	7.73	0.0048
GOTERM_CC_FAT	plasma membrane	GO:0005886	12.5%	1.80	0.006
GOTERM_CC_FAT	nuclear lumen	GO:0031981	14.6%	1.70	0.0069
GOTERM_CC_FAT	ribonucleoprotein complex	GO:0030529	15.4%	1.66	0.0083

Proteins with long C-terminal disorder (>30 aa) (n=352)

Source	Term	Term type or identifier	Percentage	Enrichment	q-value
SP_PIR_KEYWORDS	phosphoprotein	-	70.5%	1.74	3.9e-28
GOTERM_CC_FAT	nuclear lumen	GO:0031981	22.7%	2.37	3e-11
SP_PIR_KEYWORDS	nucleus	-	43.5%	1.71	5e-11
GOTERM_CC_FAT	nucleolus	GO:0005730	12.8%	2.71	1.1e-06
SP_PIR_KEYWORDS	rna-binding	-	11.4%	2.44	0.0003

Proteins with long N-terminal structure (>30 aa) (n=873)

Source	Term	Term type or identifier	Percentage	Enrichment	q-value
SP_PIR_KEYWORDS	cytoplasm	-	29.4%	1.36	7.7e-06
SP_PIR_KEYWORDS	wd repeat	-	3.9%	2.37	0.0025
SP_PIR_KEYWORDS	endoplasmic reticulum	-	8.9%	1.68	0.0029
SP_PIR_KEYWORDS	oxidoreductase	-	7.0%	1.77	0.0096

Table S1G. The results are independent of protein length
Related to **Figure S1G**.

**Property**		$\tilde{x}$ ± **C.I. (Half-Life [min])**	**n**
**Protein length**	*N-terminal disorder length*		
Small (≤350 residues)	Long (>30 residues)	56 ± 10.6	91
	Short (≤30 residues)	56 ± 4.1 (0.73)	1032
Medium (351-600 residues)	Long (>30 residues)	39 ± 5.9	161
	Short (≤30 residues)	44 ± 2.6 ($5x10^{-2}$)	947
Large (>600 residues)	Long (>30 residues)	29 ± 3.5	227
	Short (≤30 residues)	33 ± 2.0 ($3x10^{-2}$)	815
**Protein length**	*C-terminal disorder length*		
Small (≤350 residues)	Long (>30 residues)	76 ± 13.1	92
	Short (≤30 residues)	54 ± 3.9 (0.01, $\tilde{x}_{Long} > \tilde{x}_{Short}$)	1031
Medium (350-600 residues)	Long (>30 residues)	40 ± 6.7	128
	Short (≤30 residues)	44 ± 2.6 (0.30)	980
Large (>600 residues)	Long (>30 residues)	32.5 ± 4.7	132
	Short (≤30 residues)	32 ± 1.9 (0.92)	910
**Protein length**	*Internal disordered segment*		
Small (≤350 residues)	yes (≥40 residues)	45 ± 6.5	205
	no (<40 residues)	59 ± 4.5 ($2x10^{-4}$)	918
Medium (350-600 residues)	yes (≥40 residues)	35 ± 3.2	387
	no (<40 residues)	50 ± 3.2 ($2x10^{-8}$)	721
Large (>600 residues)	yes (≥40 residues)	30.5 ± 2.3	668
	no (<40 residues)	35 ± 2.9 ($4x10^{-3}$)	374

Table S1H. The results are independent of protein abundance
Data on protein abundance for *S. cerevisiae* grown in rich medium (YEPD) were obtained from Newman *et al*. (Newman et al., 2006). Proteins were divided into abundance tertiles, resulting in the low, medium and high abundance categories.

**Property**		$\tilde{x}$ ± **C.I. (Half-Life [min])**	**n**
**Protein abundance**	*N-terminal disorder length*		
Low (≤117.5 arb. units)	Long (>30 residues)	34 ± 7.5	93
	Short (≤30 residues)	41 ± 4.2 ($1x10^{-2}$)	500
Medium (117.5-324.5 a.u.)	Long (>30 residues)	46 ± 7.9	91
	Short (≤30 residues)	52 ± 4.4 ($3x10^{-2}$)	501
High (>324.5 a.u.)	Long (>30 residues)	54 ± 10.8	87
	Short (≤30 residues)	62 ± 6 ($8x10^{-2}$)	505
**Protein abundance**	*C-terminal disorder length*		
Low (≤117.5 a.u.)	Long (>30 residues)	30.5 ± 9.8	70
	Short (≤30 residues)	41 ± 3.9 ($2x10^{-2}$)	523
Medium (117.5-324.5 a.u.)	Long (>30 residues)	51.5 ± 8.1	76
	Short (≤30 residues)	51 ± 4.3 (0.39)	516
High (>324.5 a.u.)	Long (>30 residues)	54 ± 13.1	89
	Short (≤30 residues)	63 ± 5.9 (0.13)	503
**Protein abundance**	*Internal disordered segment*		
Low (≤117.5 a.u.)	yes (≥40 residues)	35 ± 4.2	262
	no (<40 residues)	45 ± 5.4 ($7x10^{-5}$)	331
Medium (117.5-324.5 a.u.)	yes (≥40 residues)	44 ± 4.6	232
	no (<40 residues)	56 ± 6 ($2x10^{-5}$)	360
High (>324.5 a.u.)	yes (≥40 residues)	44 ± 6.8	160
	no (<40 residues)	69 ± 7.2 ($1x10^{-7}$)	432

Table S1I. The trends are not affected by cytoplasmic or nuclear localization

Subcellular localization information (cytoplasm and nucleus) was obtained from the Saccharomyces Genome Database (Christie et al., 2004). Note that nuclear proteins display shorter half-lives and increased disorder relative to cytoplasmic proteins.

**Property**		$\tilde{x}$ **± C.I.**	**n**
		**Half-Life [min]**	
**Protein localization**	Nucleus	39 ± 2.3	1305
	Cytoplasm	45 ± 2.6 ($6x10^{-4}$)	1378
**Protein localization**	*N-terminal disorder length*		
Nucleus	Long (>30 residues)	33 ± 4.4	238
	Short (≤30 residues)	41 ± 2.7 ($2x10^{-4}$)	1067
Cytoplasm	Long (>30 residues)	35.5 ± 5	208
	Short (≤30 residues)	47 ± 3.1 ($2x10^{-4}$)	1170
**Protein localization**	*C-terminal disorder length*		
Nucleus	Long (>30 residues)	39.5 ± 6.1	172
	Short (≤30 residues)	39 ± 2.5 (0.61)	1133
Cytoplasm	Long (>30 residues)	44 ± 8.3	139
	Short (≤30 residues)	45 ± 2.8 (0.53)	1239
**Protein localization**	*Internal disordered segment*		
Nucleus	yes (≥40 residues)	32 ± 2.7	610
	no (<40 residues)	48 ± 3.9 ($4x10^{-14}$)	695
Cytoplasm	yes (≥40 residues)	33.5 ± 3.1	496
	no (<40 residues)	54 ± 4 ($1x10^{-19}$)	882

	**Half-life [min]**	
Nucleus	39 ± 2.3	1305
Cytoplasm	45 ± 2.6 ($6x10^{-4}$)	1378
	**N-terminal disorder length [residues]**	
Nucleus	9 ± 0.9	1305
Cytoplasm	8 ± 0.6 ($4x10^{-4}$)	1378
	**C-terminal disorder length [residues]**	
Nucleus	3 ± 0.5	1305
Cytoplasm	3 ± 0.4 (0.02)	1378

	**Internal disordered segment (≥40 residues)**		
	yes	no	
Nucleus	610	695	1305
Cytoplasm	496	882	1378
	1106	1577	

$P = 2x10^{-8}$ (chi-squared test)

	**Overall disorder degree [%]**	
Nucleus	27.3 ± 1.4	1305
Cytoplasm	19.6 ± 1.3 ($6x10^{-14}$)	1378

Table S1J. Removal of membrane proteins does not affect the observed trends

Property		$\tilde{x}$ ± C.I. (Half-Life [min])	n
**N-terminal disorder length**	Long (>30 residues)	35 ± 3.6	433
	Short (≤30 residues)	44 ± 1.8 ($3x10^{-5}$)	2585
**C-terminal disorder length**	Long (>30 residues)	43 ± 4.9	318
	Short (≤30 residues)	43 ± 1.7 (0.64)	2700
**Internal disordered segment**	yes (≥40 residues)	33 ± 2.4	1170
	no (<40 residues)	50 ± 2.4 ($2x10^{-29}$)	1848

Table S1K. Using the Kolmogorov-Smirnov test to assess half-life differences yields equivalent results

Property	***P*** **value**
**N-terminal disorder length**	
Long (>30 residues) vs. Short (≤30 residues)	$1x10^{-4}$
**C-terminal disorder length**	
Long (>30 residues) vs. Short (≤30 residues)	0.94
**Internal disordered segment**	
yes (≥40 residues) vs. no (<40 residues)	$< 2x10^{-16}$

Table S2, Related to Figures 1 and 2

Table S2 (separate Excel file). The primary data used in this study
This table is available as a separate Excel file.

Table S3, Related to Table 1

Table S3A. Extended conditional probabilities for N-terminal disorder and protein half-life
This table is an extension to **Table 1A**.

Half-life / ***N-terminal disordered segment***	**Short (≤30 min; H_S)** 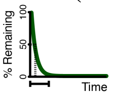	**Medium (31-70 min; H_M)** 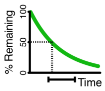	**Long (>70 min; H_L)** 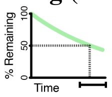	***Total***
**Long (>30 residues; N_L)** 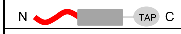	210	158	111	479
**Short (≤30residues; N_S)** 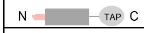	930	1023	841	2794
Total	1140	1181	952	3273

Conditional probability of observing a protein with:

(i) long N-terminal disorder among those that have short half-life:

$$P(N_L \mid H_S) = \frac{210}{1140} = 0.18$$

The low value suggests that there are factors other than long N-terminal disorder that can cause proteins to have a short half-life.

(ii) short half-life among those that have long N-terminal disorder:

$$P(H_S \mid N_L) = \frac{210}{479} = 0.44$$

The relatively higher value suggests that proteins with long N-terminal disorder are likely to be rapidly degraded.

(i) long N-terminal disorder among those that have long half-life:

$$P(N_L \mid H_L) = \frac{111}{952} = 0.12$$

The low value suggests that proteins that have a long half-life are unlikely to have long N-terminal disorder.

(ii) long half-life among those that have long N-terminal disorder:

$$P(H_L \mid N_L) = \frac{111}{479} = 0.23$$

This suggests that proteins with long N-terminal disorder do not usually have a long half-life, indicating that such proteins are often rapidly degraded.

(i) short N-terminal disorder among those that have short half-life:

$$P(N_S \mid H_S) = \frac{930}{1140} = 0.82$$

This suggests that proteins with short N-terminal disorder can still be actively degraded by other mechanisms.

(ii) short half-life among those that have short N-terminal disorder:

$$P(H_S \mid N_S) = \frac{930}{2794} = 0.33$$

This suggests that proteins with short N-terminal disorder usually do not have short half-lives.

(i) short N-terminal disorder among those that have long half-life:

$$P(N_S \mid H_L) = \frac{841}{952} = 0.88$$

The high value suggests that most long-lived proteins have short N-terminal disorder.

(ii) long half-life among those that have short N-terminal disorder:

$$P(H_L \mid N_S) = \frac{841}{2794} = 0.30$$

This suggests that proteins with short N-terminal disorder do not necessarily have long half-life, indicating that they can still be degraded by other mechanisms.

Table S3B. Extended conditional probabilities for internal disorder and protein half-life
This table is an extension to **Table 1B**.

Half-life / ***Internal disordered segment***	**Short (≤30 min; H_S)** 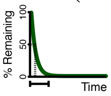	**Medium (31-70 min; H_M)** 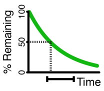	**Long (>70 min; H_L)** 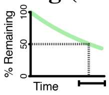	***Total***
**Present (≥40 residues; I_p)** 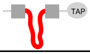	564	432	264	1260
**Absent (<40residues; I_a)** 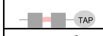	576	749	688	2013
Total	1140	1181	952	3273

Conditional probability of observing a protein with:

(i) an internal disordered segment among those that have short half-life:

$$P(I_p \mid H_S) = \frac{564}{1140} = 0.49$$

(ii) short half-life among those that have an internal disordered segment:

$$P(H_S \mid I_p) = \frac{564}{1260} = 0.45$$

(i) an internal disordered segment among those that have long half-life:

$$P(I_p \mid H_L) = \frac{264}{952} = 0.28$$

(ii) long half-life among those that have an internal disordered segment:

$$P(H_L \mid I_p) = \frac{264}{1260} = 0.21$$

(i) no internal disordered segment among those that have short half-life:

$$P(I_a \mid H_S) = \frac{576}{1140} = 0.51$$

(ii) short half-life among those that do not have an internal disordered segment:

$$P(H_S \mid I_a) = \frac{576}{2013} = 0.29$$

(i) no internal disordered segment among those that have long half-life:

$$P(I_a \mid H_L) = \frac{688}{952} = 0.72$$

(ii) long half-life among those that do not have an internal disordered segment:

$$P(H_L \mid I_a) = \frac{688}{2013} = 0.34$$

Table S4, Related to Figure 2

For information about the table content see **Table S1** above.

Table S4A. The results for internal disorder are independent of the length of N-terminal disorder of the proteins
Related to **Figure 2B**.

**Property**		$\tilde{x}$ ± **C.I. (Half-Life [min])**	**n**
**N-terminal disorder length**	*Internal disordered segment*		
Long (>30 residues)	yes (≥40 residues)	28.5 ± 3.8	226
	no (<40 residues)	43 ± 5.2 ($6x10^{-5}$)	253
Short (≤30 residues)	yes (≥40 residues)	35 ± 2.1	1034
	no (<40 residues)	50 ± 2.5 ($2x10^{-24}$)	1760

Table S4B. Function enrichment analysis of proteins with internal disordered segment(s)
For information about the table content see **Table S1F** above. Background: all yeast proteins.

Proteins with internal disordered segment(s) (≥40 aa) (n=1260)

**Source**	**Term**	**Term type or identifier**	**Percentage**	**Enrichment**	**q-value**
SP_PIR_KEYWORDS	phosphoprotein	-	74.5%	1.84	5.7e-161
SP_PIR_KEYWORDS	nucleus	-	41.8%	1.65	7.6e-44
SP_PIR_KEYWORDS	transcription regulation	-	17.8%	2.42	8.8e-43
SP_PIR_KEYWORDS	coiled coil	-	10.8%	2.18	7.3e-19
GOTERM_CC_FAT	site of polarized growth	GO:0030427	8.9%	2.25	5.1e-17
GOTERM_CC_FAT	nucleoplasm part	GO:0044451	9.2%	1.99	1.5e-12
GOTERM_BP_FAT	cell cycle	GO:0007049	18.2%	1.57	2.1e-12
SP_PIR_KEYWORDS	activator	-	6.5%	2.32	5.8e-12
GOTERM_BP_FAT	negative regulation of biosynthetic process	GO:0009890	8.6%	1.99	9.1e-12
KEGG_PATHWAY	Cell cycle	sce04111	4.4%	2.67	2.9e-11
SP_PIR_KEYWORDS	zinc-finger	-	8.2%	2.01	1.2e-10
GOTERM_MF_FAT	RNA polymerase II transcription factor activity	GO:0003702	6.0%	2.15	9.3e-10
GOTERM_BP_FAT	chromosome organization	GO:0051276	12.0%	1.68	1.3e-09
GOTERM_BP_FAT	transcription, DNA-dependent	GO:0006351	8.0%	1.90	2.6e-09
INTERPRO	Zinc finger, C2H2-like	IPR015880	2.9%	3.07	6.3e-09
SP_PIR_KEYWORDS	DNA binding	-	5.2%	2.24	2.7e-08
GOTERM_CC_FAT	chromosome	GO:0005694	10.6%	1.68	4e-08
SP_PIR_KEYWORDS	repressor	-	4.0%	2.53	4.9e-08
GOTERM_BP_FAT	cytoskeleton organization	GO:0007010	7.5%	1.83	2.7e-07
GOTERM_BP_FAT	cytokinesis	GO:0000910	4.6%	2.20	3.1e-07
GOTERM_BP_FAT	mitotic cell cycle	GO:0000278	9.5%	1.67	6.4e-07
GOTERM_BP_FAT	intracellular signaling cascade	GO:0007242	6.0%	1.90	2.6e-06
GOTERM_MF_FAT	lipid binding	GO:0008289	3.7%	2.28	6e-06
GOTERM_BP_FAT	growth	GO:0040007	5.1%	1.99	6.3e-06
GOTERM_BP_FAT	regulation of cell cycle	GO:0051726	6.1%	1.86	6.5e-06
GOTERM_CC_FAT	plasma membrane part	GO:0044459	5.8%	1.85	2.8e-05
GOTERM_MF_FAT	specific RNA polymerase II transcription factor activity	GO:0003704	2.7%	2.51	4.1e-05
GOTERM_CC_FAT	incipient cellular bud site	GO:0000131	2.3%	2.79	5.2e-05
GOTERM_MF_FAT	transcription activator activity	GO:0016563	3.0%	2.32	9.5e-05
GOTERM_BP_FAT	positive regulation of transcription	GO:0045941	5.7%	1.80	0.00012
SP_PIR_KEYWORDS	serine/threonine-protein kinase	-	3.9%	2.07	0.00027
SP_PIR_KEYWORDS	sh3 domain	-	1.3%	3.78	0.00034
SP_PIR_KEYWORDS	ATP	-	6.4%	1.71	0.00037
GOTERM_CC_FAT	transcription factor complex	GO:0005667	3.0%	2.24	0.00057
GOTERM_BP_FAT	histone modification	GO:0016570	3.7%	2.02	0.00091
GOTERM_MF_FAT	enzyme activator activity	GO:0008047	3.7%	1.96	0.002
INTERPRO	SANT, DNA-binding	IPR001005	1.2%	3.63	0.002

GOTERM_BP_FAT	response to osmotic stress	GO:0006970	3.7%	1.94	0.0026
KEGG_PATHWAY	Endocytosis	sce04144	1.4%	3.26	0.0033
GOTERM_MF_FAT	transcription repressor activity	GO:0016564	2.2%	2.36	0.005
GOTERM_BP_FAT	Ras protein signal transduction	GO:0007265	1.7%	2.70	0.0055
KEGG_PATHWAY	Meiosis	sce04113	3.3%	1.96	0.0071
SP_PIR_KEYWORDS	ubl conjugation pathway	-	3.8%	1.89	0.0078

Table S5, Related to Figure 3

For information about the table content see **Table S1** above.

Table S5. Summary of boxplot parameters and significance estimates for the effects of disordered segments on protein turnover in mouse and human

Note that the scale for protein half-life is in hours for mouse, rather than minutes as in yeast. Mouse values are half-lives, while human values are relative degradation rates. Thus, values are reversed for the human data: proteins with a short half-life have a high relative degradation rate and the other way around.

Property		$\tilde{x}$ ± C.I.	n
Mouse		**Half-Life [h]**	
**N-terminal disorder length**	Long (>30 residues)	43 ± 2.9	717
	Short (≤30 residues)	49.5 ± 1.7 ($5x10^{-5}$)	3785
**C-terminal disorder length**	Long (>30 residues)	44.5 ± 3.2	624
	Short (≤30 residues)	49 ± 1.7 ($6x10^{-2}$)	3878
**Internal disordered segment**	yes (≥40 residues)	36.5 ± 1.6	1626
	no (<40 residues)	57.5 ± 2.3 ($6x10^{-51}$)	2876
Human		**Relative degradation rate**	
**N-terminal disorder length**	Long (>30 residues)	-0.04 ± 0.07	658
	Short (≤30 residues)	-0.24 ± 0.03 ($9x10^{-8}$)	3313
**C-terminal disorder length**	Long (>30 residues)	-0.05 ± 0.07	625
	Short (≤30 residues)	-0.23 ± 0.03 ($7x10^{-7}$)	3346
**Internal disordered segment**	yes (≥40 residues)	0.09 ± 0.05	1493
	no (<40 residues)	-0.37 ± 0.03 ($4x10^{-65}$)	2478

Table S6, Related to Figure 4

For information about the table content see **Table S1** above.

Table S6A. Divergence of N-terminal or internal disordered segments is linked to protein half-life changes during evolution

Related to **Figures 4B**, **4C** and **S4A**.

'SS & LL' refers to paralog pairs where both proteins have the same type of terminal disorder, short (S) or long (L). 'SL' refers to divergent paralog pairs, where one protein has a short (S) and the other a long (L) disordered terminus. '$\Delta I = 0$' refers to paralog pairs with identical numbers of internal disordered segments. '$\Delta I \geq 1$' refers to paralog pairs where one of the two paralogs has a higher number of internal disordered segments. IL refers to the total number of residues that make up all internal disordered segments. See the **Supplemental Experimental Procedures** for details.

**Property**		$\tilde{x}$ **± C.I.**	**n**
**N-terminus**			
		**ΔH [min]**	
N-terminal disorder length difference ΔL	SS & LL	-2 ± 3.4	1049
	SL	-14 ± 6.6 ($9x10^{-6}$)	391
		**ΔL [# residues]**	
Half-life difference ΔH	≤0 min	12 ± 3.5	804
	>0 min	8 ± 1.6 ($1x10^{-5}$)	636
**C-terminus**			
		**ΔH [min]**	
C-terminal disorder length difference ΔL	SS & LL	7 ± 3.2	1154
	SL	-5.5 ± 7.5 ($3x10^{-3}$)	286
		**ΔL [# residues]**	
Half-life difference ΔH	≤0 min	5 ± 1.5	658
	>0 min	4 ± 0.8 (0.67)	782
**Internal**			
Internal disordered regions difference ΔI		**ΔH [min]**	
(a) $\Delta I = 0$;	$\Delta H = H_1 - H_2$, where $IL_1 \leq IL_2$	4 ± 4.1	879
(b) $\Delta I = 0$;	$\Delta H = H_1 - H_2$, where $IL_1 \geq IL_2$	-5 ± 4 ($2x10^{-5}$)	879
	(c) $\Delta I \geq 1$	-7 ± 4.1 ($1x10^{-5}$)	561

Table S6B. Approximate permutation tests for N-terminal disorder in paralog pairs

This table shows the results of four separate approximate permutation analyses, focused on the effect on half-life of changes in N-terminal disorder between pairs of paralogs (**Supplemental Experimental Procedures**). Averaged medians resulting from 1000 random permutations of the length of N-terminal disorder, protein half-lives, both, or random assignments of paralog pairs from BLASTClust singletons (those proteins lacking a paralog in yeast) are shown. The other properties were kept intact in each case, while the randomized property was drawn from its distribution without replacement. The *P* values reported here are equal to the fraction of Mann-Whitney *U* tests that gave a lower or identical *P* value to the one observed in the real data (**Figure 4** and **Table S6A**). A significant *P* value here indicates that our findings are not likely to have been observed by chance. 'SS & LL' refers to paralog pairs where both proteins have the same type of terminal disorder, short (S) or long (L). 'SL' refers to divergent paralog pairs, where one protein has a short (S) and the other a long (L) disordered terminus.

Randomly assigned property		Average of medians	Permutations
**N-terminal disordered regions**			
		**ΔH [min]**	
N-terminal disorder length difference ΔL	SS & LL	0.187	1000
	SL	0.106 (3x10^{-2})	
		**ΔL [# residues]**	
Half-life difference ΔH	≤0 min	9.073	1000
	>0 min	8.875 (4x10^{-3})	
**Half-lives**			
		**ΔH [min]**	
N-terminal disorder length difference ΔL	SS & LL	0.150	1000
	SL	-0.255 (4x10^{-2})	
		**ΔL [# residues]**	
Half-life difference ΔH	≤0 min	9.95	1000
	>0 min	9.904 (1x10^{-2})	
**N-terminal disordered regions and half-lives**			
		**ΔH [min]**	
N-terminal disorder length difference ΔL	SS & LL	-0.049	1000
	SL	0.118 (4x10^{-2})	
		**ΔL [# residues]**	
Half-life difference ΔH	≤0 min	9.917	1000
	>0 min	9.872 (8x10^{-3})	
**Paralog pairs from singletons**			
		**ΔH [min]**	
N-terminal disorder length difference ΔL	SS & LL	-1.321	1000
	SL	-3.385 (<1x10^{-3})	
		**ΔL [# residues]**	
Half-life difference ΔH	≤0 min	10.201	1000
	>0 min	9.183 (<1x10^{-3})	

Table S6C. Function enrichment analysis of paralog pairs that diverged in N-terminal or internal disordered segments
For information about the table content see **Table S1F** above. Background: paralogous proteins in yeast (see **Supplemental Experimental Procedures**).

Proteins in paralog pairs where N-terminal disorder length diverged (short & long) (n=251 unique proteins in 391 pairs)

Source	Term	Term type or identifier	Percentage	Enrichment	q-value
SP_PIR_KEYWORDS	serine/threonine-protein kinase	-	14.7%	3.82	6.6e-11
INTERPRO	Helicase, superfamily 1 and 2, ATP-binding	IPR014021	8.4%	4.80	2.3e-07
GOTERM_MF_FAT	RNA helicase activity	GO:0003724	6.8%	4.43	7.8e-05
INTERPRO	Amino acid permease, fungi	IPR004762	4.4%	5.34	0.005

Proteins in paralog pairs that diverged in the number of internal disordered segments (n=369 unique proteins in 561 pairs)

Source	Term	Term type or identifier	Percentage	Enrichment	q-value
SP_PIR_KEYWORDS	ATP	-	17.3%	2.48	7.7e-11
SP_PIR_KEYWORDS	phosphoprotein	-	65.3%	1.35	8.9e-10
SP_PIR_KEYWORDS	ubl conjugation pathway	-	5.4%	4.07	5.2e-06
SP_PIR_KEYWORDS	nucleotide binding	-	10.0%	2.60	9.8e-06
KEGG_PATHWAY	Cell cycle	sce04111	6.0%	3.41	2.3e-05
GOTERM_BP_FAT	cell division	GO:0051301	13.0%	1.97	0.0012
GOTERM_BP_FAT	response to organic substance	GO:0010033	8.1%	2.33	0.0052

Table S7, Related to Figure 4

Table S7 (separate Excel file). Data on N-terminal disordered regions, internal disordered regions, and half-life for paralog pairs
This table is available as a separate Excel file.

Table S8, Related to Table 2

Table S8A. Extended conditional probabilities for N-terminal disorder and protein half-life in pairs of paralogs
This table is an extension to **Table 2A**.

	**Negative half-life difference (ΔH < 0 min)**	**Positive half-life difference (ΔH > 0 min)**	***Total (including $\Delta H = 0$ min)***
**Short-short(SS) & Long-long(LL)** similar 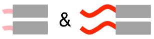	535	501	1049
**Short-long(SL)** divergent 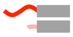	252	135	391
Total	787	636	1440

Conditional probability of observing a paralogous pair with:

(i) negative half-life difference among all pairs where one paralog has long, the other short N-terminal disorder:

$$P(\Delta H < 0\,\text{min} \mid N_{SL}) = \frac{252}{391} = 0.64$$

The relatively high value suggests that paralogous pairs that have diverged the length of their N-terminal disorder are likely to have altered half-life in a manner that is consistent with the reported observation (i.e. proteins with longer terminal disorder tend to have shorter half-life).

(ii) one long, one short disorder N-terminus among all pairs with negative half-life difference:

$$P(N_{SL} \mid \Delta H < 0\,\text{min}) = \frac{252}{787} = 0.32$$

The relatively low value suggests that regulatory mechanisms other than divergence in N-terminal disorder control the turnover of individual proteins after gene duplication.

Table S8B. Extended conditional probabilities for internal disorder and protein half-life in pairs of paralogs
This table is an extension to **Table 2B**.
IL refers to the total number of residues that make up all internal disordered segments. $I_{n \Rightarrow n+x}$ denotes pairs where one of the two paralogs has a higher number of internal disordered segments (ΔI ≥1). See the **Supplemental Experimental Procedures** for details.

	**Negative half-life difference (ΔH < 0 min)**	**Positive half-life difference (ΔH > 0 min)**	***Total (including $\Delta H = 0$ min)***
**Identical number of internal disordered segments (ΔI = 0)** paralog pair, ancestral protein 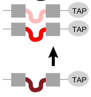	476 *($\Delta H = H_1 - H_2$, with $IL_1 \geq IL_2$)*	391 *($\Delta H = H_1 - H_2$, with $IL_1 \geq IL_2$)*	879
**Diverged number of internal disordered segments (ΔI ≥1)** paralog pair, ancestral protein 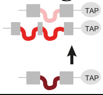	323	233	561
Total	799	624	1440

Conditional probability of observing a paralogous pair with:

(i) negative half-life difference among all pairs where one paralog has more internal disordered segments than the other:

$$P(\Delta H < 0\,\text{min} \mid I_{n \rightarrow n+x}) = \frac{323}{561} = 0.58$$

(ii) pairs with a diverged number of internal disordered segments among all pairs with negative half-life difference:

$$P(I_{n \rightarrow n+x} \mid \Delta H < 0\,\text{min}) = \frac{323}{799} = 0.40$$

SUPPLEMENTAL RESULTS

Results S1. The effects of disordered segments on half-life are independent of the overall disorder degree (extended)

The fraction of disordered residues (i.e. overall degree of disorder), which is an estimate of the packing, folding and structural stability of a protein, by itself correlates with protein half-life, although previous studies disagree on the extent of the effect (Gsponer et al., 2008; Tompa et al., 2008; Yen et al., 2008). Proteins with a greater overall disorder degree generally contain longer terminal and internal disordered segments (**Figure S3A**). To determine whether the effects of continuous stretches of disordered residues (i.e. disordered segments) on protein turnover (**Figures 1** and **2**) are independent of the overall degree of disorder, we matched proteins that have a similar fraction of disordered residues but have varying combinations of disordered segments (long or short N-terminal disorder and/or presence or absence of internal disordered segments; **Figure S3B**).

For each protein with a long N-terminal disordered segment that also has a long internal disordered segments (N_LI_p, 226 cases, the minority class) we selected one protein from each of the other classes that is closest to the N_LI_p protein in terms of the overall disorder degree (i.e. fraction of disordered residues across the full protein). This yields combinations of proteins from different classes (N_LI_p - N_SI_p - N_LI_a – N_SI_a) with almost identical overall degrees of disorder (**Figure S3B**). R values between the overall disorder degree of pairs of proteins from various classes are ~1 (almost perfect linear correlation), demonstrating that paired proteins selected using the above method indeed have almost identical overall degrees of disorder. Comparison of the half-lives of proteins from different classes with similar overall disorder degrees (**Figure S3C**) reveals similar trends as the analysis that uses all proteins (**Figure 2D**). Proteins with both long N-terminal and internal disordered segments (N_LI_p) typically have the shortest half-lives, independent of whether all proteins are considered, or only proteins with highly similar overall disorder degree. Then come proteins with either long internal (N_SI_p), or long N-terminal disordered segments (N_LI_a). Proteins with no long disordered segments (N_SI_a) typically have the longest half-lives.

The effect sizes of the differences between the half-life distributions are comparable when proteins are grouped based on the overall degree of disorder or when all proteins are considered (**Figure S3D**, upper triangles). Furthermore, most half-life distributions are significantly different, though *P* values for proteins grouped by overall disorder degree are less significant than when using all proteins due to smaller sample sizes (**Figure S3D**, lower triangles). Alternative comparison of proteins by the number of internal disordered segments (**Figures S3I**, **S3J** and **S3K**), and divided into structural classes based on the scores of the various disorder predictors (**Figures S1C**, **S1D**, **S2A** and **S2B**) confirms that the observed effects are independent of the overall degree of disorder. These results indicate that long disordered segments (continuous stretches of disordered residues) at the N-terminus or internally contribute to shorter protein half-life in living cells, and that this effect is independent of the fraction of disordered residues across the whole protein. It should however be noted that this does not rule out an additional effect of the overall degree of disorder on protein half-life, i.e. among proteins that do or do not have a disordered segment, proteins with higher degrees of overall disorder tend to have a lower half-life compared to those with a lower degree of disorder (see **Discussion** in the main text).

Comparing individual classes of proteins with disordered segments

We also compared the individual classes of proteins with disordered segments (N_LI_p, N_SI_p, or N_LI_a) to proteins without any such segments (N_SI_a). For that, we paired each N_SI_a protein with a protein from each of the other classes that has the closest overall degree of disorder (making N_LI_a - N_SI_a, N_SI_p - N_SI_a, and N_LI_p - N_SI_a pairs, **Figure S3E** and **S3F**). This is different from the approach above, which makes combinations of N_LI_p - N_SI_p - N_LI_a – N_SI_a proteins. We then compared the half-lives. One confounding factor in the approach for pairing proteins with disordered segments to proteins with no disordered segments (N_SI_a) based on the overall disorder degree, is that a N_SI_a protein can be sampled more than once if it happens to be closest in terms of overall disorder degree to multiple proteins with disordered segments. This effect is most pronounced in the N_SI_p - N_SI_a and N_LI_p - N_SI_a pairs (**Figure S3G**), but does not seem to be a problem since many N_SI_a proteins occur few (more than once, but not very often) times, rather than there being very few N_SI_a proteins that account for very many of the data points. E.g. in the N_SI_p - N_SI_a pairs, 10.2% of the non-unique proteins account for 34.6% of all data points, and in the N_LI_p - N_SI_a pairs, 9.6% of the non-unique proteins account for 27.8% of all data points. None of the three pairings have a single or a couple of proteins that are present an extreme number of times (**Figure S3G**, 'Maximal occurrences' column).

Nevertheless, to control for potential biases caused by having duplicate data points in the group of proteins with no disordered segment, we randomly removed protein pairs containing a multiple-occurring N_SI_a protein, until individual proteins occur only once. We then calculated effect sizes and statistical differences between half-life distributions of proteins with and without disordered segments as before. The procedure was repeated 1000 times to estimate the robustness of the results. The means and medians of the resulting effect sizes (difference in median half-life) are close to the original values for N_LI_a - N_SI_a and N_LI_p - N_SI_a (**Figure S3H**). The effect sizes for the N_SI_p - N_SI_a comparison differ a lot more between the analysis that includes multiple-occurring N_SI_a proteins (**Figure S3F**), and the analysis that excludes such duplicates (**Figure S3H**): not a single of the 1000 N_SI_p - N_SI_a sets results in an effect size that is as big (28 minutes)

as the set that includes duplicates. This has likely to do with the larger number of duplicates in the N_SI_p - N_SI_a comparison than in N_LI_a - N_SI_a and N_LI_p - N_SI_a (**Figure S3G**). Importantly, however, the effect sizes of the comparisons without duplicates are in all cases very similar to the values when using all data, i.e. not combined by disorder degree similarity (minimally 8.5 min. for N_LI_a - N_SI_a, 14 min. for N_SI_p - N_SI_a, 27 min. for N_LI_p - N_SI_a - **Figure S3H** - compared to the upper triangle of the 'All proteins' part of **Figure S3D** - 7, 15, and 22 min., respectively).

The *P* values are generally less significant due to a reduction in the number of data points (**Figure S3H**). About three quarters of the 1000 generated N_SI_a sets for the N_LI_a - N_SI_a pairing have significantly higher half-life distributions than the corresponding N_LI_a set at a confidence level of 5%. The smallest effect size is still 8.5 minutes, which is substantial in the context of the yeast cell cycle and comparable to the difference found in the original comparison of half-lives between proteins with long and short N-terminal disordered segments (**Figure 1**, 9 minutes). Furthermore, all 1000 tested N_SI_a sets in the N_SI_p - N_SI_a and N_LI_p - N_SI_a pairings have significantly longer half-lives compared to the paired proteins with a disordered segment, with large effect sizes (**Figure S3H**). Together, these results again indicate that presence of disordered segments leads to shorter protein half-life and that this is likely to be independent of the overall degree of disorder.

Our approach for selecting protein pairs with similar overall disorder degree made sure that the differences between the overall disorder degrees of the individual proteins are fully controlled for (**Figure S3B** and **S3E**). We did however ask how often, within the protein pairs with similar overall disorder degree, the protein with a disordered segment (N_LI_p, N_SI_p, or N_LI_a) has the highest fraction of disordered residues and the protein without a disordered segment (N_SI_a) the lowest, because if the proteins with a disordered segment always have the higher overall disorder degree, then overall disorder degree might still be the cause of their shorter half-life (even though this is highly unlikely due to the negligible differences in overall disorder degree between protein pairs). In all three pairings, the percentage of pairs where the protein with a disordered segment has the higher overall disorder value compared to the N_SI_a protein is close to 50%, being 44.7% (N_LI_a), 48.1% (N_SI_p), and 53.5% (N_LI_p). Therefore, we can discard biases towards higher overall disorder degree values in proteins with disordered segment as a possible reason for why these proteins typically have shorter half-lives compared to proteins without such segments.

Results S2. The results are independent of known degradation signals

Aside from ubiquitination, several signals have been shown to regulate protein degradation. For example, short sequence motifs, such as the destruction box and the KEN box act as recognition surfaces for ubiquitin ligases and thereby signal for the destruction of specific proteins, mostly involved in the cell cycle (Pfleger and Kirschner, 2000). Another feature of proteins that has been proposed to lead to rapid degradation is the presence of regions that are enriched in proline, glutamic acid, serine, and threonine residues (PEST regions), although the mechanism is unknown (Rogers et al., 1986). To investigate if the effects of disordered segments on protein half-life could be explained by the presence of such destruction signals, we collected data describing the presence of four known signals: experimentally determined ubiquitination sites, KEN box motifs, destruction box motifs, and PEST sequence regions. Experimentally determined ubiquitination sites were obtained from UniProtKB/Swiss-Prot release 2011_04 (Uniprot-Consortium, 2011). PEST regions were predicted using epestfind with default parameters, as included in EMBOSS 6.5.7 (Rice et al., 2000). Matches marked as "potential" were included, while lowest-confidence "poor" matches were excluded. KEN box and destruction box motifs were predicted using GPS-ARM 1.0 with default parameters (Liu et al., 2012). When determining overlap between a motif and a disordered region, partial overlap was considered sufficient.

Only 13% of experimentally determined yeast ubiquitination sites fall into the long terminal or internal disordered segments examined in our study. Similarly, only 28% of predicted KEN box motifs, 24% of destruction box motifs, and 45% of PEST sequences fall into these disordered segments. Furthermore, more than half (56% of all proteins, 54% of proteins with short half-life) of the proteins with long terminal or internal disordered segments do not contain a single destruction signal (experimentally determined ubiquitination sites, KEN box, destruction box, or PEST sequence) in these disordered segments. This number is conservative since many of the predicted motifs will not be biologically relevant, and because it describes the presence of any of these destruction signals. The fraction of proteins with no individual destruction signals in the long terminal or internal disordered segments is much higher: 99% for ubiquitination sites, 93% for KEN box, 90% for destruction box, and 66% for PEST regions. Consistent with this, the distributions of half-lives of proteins with and without predicted destruction signals within the disordered regions were not significantly different ($P = 0.1$ for N-terminal disorder; $P = 0.2$ for internal disorder; Mann-Whitney *U* test; data not shown). Finally, the probability that a protein has a short half-life, given that it has a long terminal or internal disordered segment is similar regardless of whether we consider the presence of destruction signals in the whole protein (0.39) or not (0.43, **Table 1C**). These results indicate that many disordered segments examined in our study contain no predicted destruction motifs or experimentally determined ubiquitination sites that could explain the effects of these segments on protein half-life. Thus, the short half-life of proteins with long disordered segments is likely due to the direct effects of these segments on proteasomal degradation, rather than due to indirect effects by incorporating destruction signals.

Results S3. Disordered segments of proteins with short half-life lack enriched, uncharacterized sequence motifs that could explain the rapid degradation

We have shown that the effects of disordered segments on protein half-life are unlikely to result from the presence of known destruction signals (ubiquitination sites, KEN box, destruction box, or PEST sequence) in these regions (**Supplemental Results S2**). Another possible explanation for the short half-life of proteins with long disordered segments is that these segments are enriched for uncharacterized sequence patterns such as short linear motifs (SLiMs) (Davey et al., 2012) that confer susceptibility to rapid degradation. These hypothetical motifs could represent novel protein degradation biology as they might facilitate for example interaction with the proteasome or serve as docking motifs for ubiquitin ligases, leading to faster turnover. To discover such uncharacterized motifs that might be responsible for the effects of disordered regions on protein degradation, we used SLiMFinder (Davey et al., 2010) and DiliMot (Neduva et al., 2005) to analyze the amino acid sequences of long N-terminal disordered segments (210 sequences in 210 proteins) and long internal disordered segments (999 sequences in 564 proteins) of proteins with short half-life (half-life ≤30 minutes).

SLiMFinder uses BLAST (Altschul et al., 1990) to identify short linear motifs that are shared by unrelated proteins. We used SLiMFinder version 4.5 with the following settings (parameters that are not reported were set with default values):

- dismask=F. We chose not to mask structured regions as we preferred to rely on the definitions of disordered and structured protein regions by DISOPRED2 used in our other analyses (**Supplemental Experimental Procedures**), which were already used to select sequences in which to search for over-represented motifs (i.e. only the sequences of long disordered regions of proteins with short half-lives were searched).
- ftmask=F. The whole sequence corresponding to a disordered region was considered for searching motifs, rather than masking parts of the sequences that correspond to annotated uniprot features such as transmembrane helices and protein domains.
- compmask=5,8. Prevents the detection of low-complexity repeat-like motifs, such as poly-Q stretches, which are common in disordered regions (Jorda et al., 2010; Simon and Hancock, 2009).
- metmask=T/F. This mask is activated for the detection of motifs in N-terminal disordered regions, because artefactual motifs starting with a methionine were reported otherwise. The mask is disabled, however, for internal disordered sequences.
- consmask=F. Less conserved parts of the sequences were not masked as we considered the whole sequence corresponding to a disordered segment for searching motifs.
- posmask=F. We have no reason to assume over-representation of certain position-specific amino acids. For example, we found no enrichment of alanines after the N-terminal initiator methionine in the N-terminal disordered sequences analyzed for motifs.
- slimlen=10. Annotated instances of short linear motifs are usually 3-10 amino acids long (Davey et al., 2012; Dinkel et al., 2012).
- maxwild=3. The vast majority (>90%) of consecutive wildcard positions in definitions of known motif classes in the ELM database (Dinkel et al., 2012) are up to three residues in length.

The motif KR..[DE] occurs 27 times in 201 sequence clusters of long N-terminal disordered segments from proteins with short half-life ($P_{corrected} = 3.9 \times 10^{-2}$, enrichment after Bonferroni-like correction for testing multiple motifs). SLiMFinder also detects 25 occurrences of the overlapping motif L.{0,1}KR (which itself is not significantly enriched, $P_{corrected} = 5.7 \times 10^{-2}$). Together, these two motifs are present in 39 of 210 (~19%) long N-terminal disordered segments of short-lived proteins, which means that the majority of such regions (more than 80%) do not contain the enriched motifs.

Long internal disordered segments from proteins with short half-life are enriched for several groups of motifs composed of short and simple overlapping instances (typically three residues in length with all positions defined, i.e. no wildcard positions). They contain largely positively charged motifs (e.g. KRK), serine/proline-rich motifs (e.g. SLP), and several singular motifs such as the negatively charged DEE motif. These motifs are in agreement with the general sequence preferences of disordered regions: enrichment for charged and polar amino acids and depletion of hydrophobic amino acids (Romero et al., 2001). Thus, these motifs seem to reflect general sequence characteristics of protein disorder rather than being sequences that for example regulate specific interactions between the proteasome and its substrates. Furthermore, combined, the enriched motifs are present in the internal disordered segments of 462 of 999 proteins with short half-life (~46%), which again shows that most short-lived proteins do not even contain the enriched motifs in their disordered segments.

DiliMot (Neduva et al., 2005) did not detect any enriched motif for either long N-terminal or internal disordered sequences of short-lived proteins. DiliMot was set to detect motifs that are fixed in at least two positions (L parameter), could be up to 10 residues in length (W parameter), and occur in at least three of the sequences searched. We tried various combinations of settings: removing or keeping parts of the sequence that (i) overlap with known domains and (ii) show similarity with other sequences in the set, using or not using information on evolutionary conservation based on (i) only other yeast species or (ii) all available species including species that are distant from *Saccharomyces cerevisiae* such as human and mouse.

Taken together, sequence analysis indicates that the majority of long disordered segments from proteins with short half-lives lack enriched, uncharacterized sequence motifs that could facilitate degradation. Furthermore, even the identified motifs that are enriched in the disordered sequences of short-lived proteins are unlikely to represent uncharacterized degradation motifs but rather reflect the general sequence properties of disordered regions. However, different subtypes of protein disorder exist, that could each have a different effect of protein half-life: some types might be able to interact with the proteasome to speed up degradation, while others might not (see **Discussion** in the main text). The broad distributions of half-lives observed in our study support this idea as they reflect the combined properties of many possible subtypes of disordered segments, some of which are able to efficiently initiate degradation, while others may not.

Results S4. Paralogous pairs with a negative half-life change have larger divergence in the length of N-terminal disorder

Paralogous proteins pairs that during evolution diverged in the length of the N-terminal disordered segments generally show changes in half-life in a manner that is in agreement with the relationship that is reported in the main text: the protein of a paralogous pair with longer N-terminal disorder usually has a shorter half-life compared with its paralog (**Figure 4B**).

We also calculated, for every pair of paralogs, the difference in the half-life of the proteins. Pairs where the paralog with the longer disordered N-terminus has the shorter half-life of the two are assigned to one group ($\Delta H \leq 0$ minutes), whereas pairs where the paralog with the longer disordered N-terminus has the longer half-life ($\Delta H > 0$ minutes) are assigned to the other group. Consistently, we find that paralogous pairs with a negative half-life change show significantly larger divergence in the length of N-terminal disorder ($P = 1 \times 10^{-5}$, Mann-Whitney *U* test, **Figure S4A**). This means that if the half-life of two paralogous proteins differs in a manner that is in agreement with the previous observations (i.e. longer disordered terminus, shorter half-life), then the changes in the length of N-terminal disorder are generally much bigger compared to paralogs that differ in their half-lives the other way around. Thus, it appears that divergence in N-terminal disorder does indeed result in a change in half-life of paralogous proteins in a manner consistent with what is reported in the main text.

Results S5. The reported trends are independent of confounding factors

We performed a number of control calculations to ensure that the observations are independent of confounding factors. The findings are independent of the method used to predict intrinsic disorder in proteins: the DISOPRED2 calculations were repeated using two alternative methods, IUPred and PONDR VSL1, which employ distinct prediction strategies and gave consistent results (**Supplemental Experimental Procedures**, **Figures S1C**, **S1D** and **S2A-C**, **Tables S1B-D**). The conclusions are also independent of different criteria and cutoffs used to group the proteins, including the overall degree of disorder (**Supplemental Results S1** and **Figure S3**), the average disorder scores both for the entire protein (**Figures S1C** and **S2A**) and for the disordered regions alone (**Figures S1D** and **S2B**), and the cutoffs used for detecting terminal and internal disordered segments (**Table S1E)** and different half-life groups (**Figures 1E**, **S1E** and **S1F**). Furthermore, outlier half-life values (**Supplemental Results S6**), protein length (**Supplemental Results S7**, **Figure S1G**, **Table S1G**), protein abundance (**Table S1H**), subcellular localization (cytoplasm versus nucleus, **Table S1I**), and the removal of membrane proteins, which may be degraded in a proteasome-independent manner (**Supplemental Results S8** and **Table S1J**), did not affect the observed trends. The results are independent of known degradation signals (**Supplemental Results S2**) and uncharacterized sequence motifs that could facilitate degradation (**Supplemental Results S3**). An analysis of residues following the initiator methionine indicates that the nature of the N-terminal residue does not account for the global difference in half-life between proteins with long or short N-terminal disorder (**Supplemental Results S9** and **Figure S1H**).

The observations on paralogs in yeast are similar if we do not consider paralogs that originated from the ancestral whole genome duplication (**Figure S4B**). Moreover, the results on paralogous pairs are robust in different kinds of permutation tests (**Table S6B** and **Supplemental Experimental Procedures**). We could not perform the paralog analysis in mouse or human because the mass spectrometry strategy used for measuring half-life is unable to differentiate between similar proteins with identical short peptide regions, such as paralogs and different splice forms (**Supplemental Experimental Procedures**). Finally, though the distributions in our analyses are broad and overlap, most differences are significant with both the Mann-Whitney *U* test and the Kolmogorov-Smirnov test (**Supplemental Experimental Procedures** and **Table S1K**), which are two distinct non-parametric statistical tests for evaluating whether two samples of observations come from the same distribution or not. Thus, the reported trends on protein half-life appear attributable to the presence and number of sufficiently long terminal and internal disordered segments.

Results S6. Highly stable proteins with undetermined or outlier half-life are generally less disordered

We discarded 366 proteins with a half-life of exactly 300 minutes, as the original paper (Belle et al., 2006) assigned this value to stable proteins for which degradation curves could not be fitted by an exponential decay function and thus half-life could not be determined. Moreover, to make sure that clear outliers would not affect the statistics, we removed seven

proteins with extremely long half-lives of >6000 minutes from the data.

To make sure that removal of these 373 proteins did not bias our analyses, we investigated the presence and length of disordered segments at various locations in these 'highly stable' proteins as well as their overall degree of disorder. We found that:

1. The removed, highly stable proteins contain significantly less disorder than the 3273 proteins in our main dataset: they tend to have shorter N-terminal disordered segments, less total internal disorder, and be more structured overall ($P = 2 \times 10^{-2}$, $P = 5 \times 10^{-8}$, $P = 3 \times 10^{-6}$, respectively, Mann-Whitney *U* tests, data not shown).
2. The discarded, stable proteins less often have a long (>30 residues) N-terminal disordered segment (51/373=13.7%) than proteins in our main dataset (479/3273=14.6%), although this difference is not statistically significant (odds ratio = 0.94; $P = 0.7$, chi-squared test).
3. There is a significant difference in the number of proteins that have a long internal disordered segment (94/373=25% of discarded proteins, 1260/3273=38% of included proteins; odds ratio = 0.66; $P = 6 \times 10^{-7}$, chi-squared test).
4. As mentioned in the main text, the experimental method for measuring protein half-lives involved C-terminal tagging with a TAP-tag (Belle et al., 2006). As a result, proteins with long and short C-terminal disordered segments display similar distributions of protein half-life (**Figure 1C**). The distribution of lengths of the disordered segments at the C-terminus as characterized from the original genome sequence is highly similar for proteins in our main dataset and for discarded highly stable proteins ($P = 0.3$, Mann-Whitney *U* tests, data not shown), which falls in line with the idea that the TAP-tag shields the contribution of the disordered segment at the C-terminus to half-life.

In short, the 373 highly stable proteins that we discarded in our main analyses generally contain shorter and less disordered segments, which, in line with the findings from our study, corresponds to them having long half-lives. Thus, removal of these highly stable proteins with long half-lives does not bias the results of our analyses, but rather strengthens our conclusions.

Results S7. The results are independent of protein length

Size is one of the determinants of the *in vivo* degradation rate of a protein, with large proteins being degraded more quickly than smaller ones (Dice et al., 1973). This observation has been confirmed by analyses of large-scale protein half-life data (Belle et al., 2006; Tompa et al., 2008). To ensure that our observations regarding the presence of disordered segments and protein half-life are not influenced by protein length, we classified the proteome into three groups of roughly equal size: (i) small proteins (≤350 residues), (ii) medium size proteins (351-600 residues), and (iii) large proteins (>600 residues). Indeed, large proteins have a significantly shorter half-life than small proteins (**Figure S1G**). For each length group, we further divided the proteins into those that contain a long or short N-terminal disordered segment, a long or short C-terminal disordered segment, or an internal disordered segment. Where sample size is sufficient, we find that, regardless of protein size, the distribution of half-life values for proteins with long N-terminal disorder is significantly smaller than that of proteins with short N-terminal disorder (**Figure S1G** and **Table S1G**). There is generally no difference in half-life between proteins with long or short C-terminal disorder (**Figure S1G** and **Table S1G**). An exception to this appears to be the group of small proteins, although the number of small proteins with a long disordered C-terminus is small (92 cases). Finally, proteins that contain an internal disordered segment have a significantly shorter half-life, irrespective of their length (**Figure S1G** and **Table S1G**). These results suggest that our observations are independent of protein length.

Results S8. Degradation of membrane proteins

The majority of transmembrane proteins in eukaryotic cells are degraded in the lumen of lysosomes, independent of the proteasome (Piper and Katzmann, 2007; Raiborg and Stenmark, 2009). Ubiquitin functions as the signal that specifies which membrane proteins should be degraded. Following ubiquitination, endocytosis brings membrane proteins inside the cell into early endosomes. Subsequently, a variety of protein sorting machines, such as the endosomal sorting complex required for transport (ESCRT), sort the ubiquitin-flagged proteins to multivesicular endosomes (MVEs) or bodies (MVBs). These then fuse with a lysosome, where proteases in the acidic lumen digest the vesicles. It should be noted that proteasome-mediated degradation of membrane proteins does occur to some extent through the process of endoplasmic reticulum–associated degradation (ERAD) (Meusser et al., 2005; Vembar and Brodsky, 2008). However, this mechanism seems to apply mainly to damaged or misfolded membrane proteins and does not seem to be responsible for degradation of the majority.

To ensure that our observations regarding the effects of disordered segments on protein half-life do indeed apply to degradation by the proteasome, we performed control calculations on datasets that lack membrane proteins. We obtained a set of yeast membrane proteins from Österberg *et al.* (Osterberg et al., 2006), subtracted these proteins (255) from our original dataset and redid the analysis. We find that the membrane proteins, which are degraded mainly in a proteasome-independent manner, do not influence our conclusions (**Table S1J**).

Even though the effects of disordered segments on protein half-life are primarily mediated through interaction with the proteasome, which is not the main route for degradation of transmembrane proteins, we were still interested to look into a potential connection between disordered regions in membrane proteins and their turnover rates. Therefore we asked: Do membrane proteins with long terminal or internal disordered segments in their cytosolic loops have shorter half-lives? To answer this question, we obtained information on membrane protein topology from Kim *et al.* (Kim et al., 2006). In that study, the location of the C-terminus of multiple-spanning (two or more transmembrane helices) membrane proteins in yeast was determined using C-terminally tagged constructs. Cytosolic or extracellular location of the C-terminus was used to constrain topology predictions by TMHMM (Melen et al., 2003). We inferred the disorder status of every residue in the predicted cytosolic loops and integrated this information with the half-life data (220 proteins in total). We then grouped proteins as in the original analyses: (i) by the length of the disordered termini if these are present on the cytosolic side (short, ≤30 residues; long, >30 residues), treating the N- and C-termini separately, and (ii) by the presence or absence of long internal disordered segments (at least 40 residues) in the cytosolic loops.

Membrane proteins with a cytosolic, long disordered N-terminus (27 in total) have a significantly shorter half-life compared to the group with no cytosolic N-terminus, or with a cytosolic, short disordered N-terminus ($P = 7 \times 10^{-3}$, Mann-Whitney *U* test, data not shown). Half-lives of membrane proteins with cytosol-localized long disordered segments at the C-terminus (which is tagged with a TAP-tag for the half-life measurements) are similar to half-lives of membrane proteins with no cytosolic C-terminus, or with a short disordered segment at the C-terminus ($P = 0.3$, Mann-Whitney *U* test, data not shown). These results are similar to the observed effects of terminal disordered segments on half-life for non-membrane proteins. However, proteins with one or more cytosol-localized long internal disordered segment (15 in total) have similar half-lives to proteins without such segments ($P = 0.76$, Mann-Whitney *U* test, data not shown). This is in contrast to the typically strongly decreased half-life of proteins with internal disordered segments for non-membrane proteins.

As discussed above, these results might be less biologically meaningful due to the established difference in mechanisms by which membranous and intracellular proteins are degraded. It should also be noted that the numbers of membrane proteins in each 'disorder' category are very small (27, 28 and 15), and much smaller than the numbers for non-membrane proteins. Furthermore, the disorder prediction algorithms that are the basis for calculating the presence of disordered segments have been developed for cytosolic proteins and are biased towards the amino acid composition of such proteins. Membrane proteins have different sequence compositions, which means that the confidence in the identified cytosolic disordered segments is lower. Thus far, no predictor has been developed specifically for identifying structural disorder in the loops of membrane proteins. Taking these points into account, the current observations could mean that membrane proteins with long terminal disordered regions might be more susceptibility to degradation, though these effects are not seen for internal disordered segments.

Results S9. The relationship between N-terminal disorder and half-life does not appear connected to the N-end rule

One mechanism regulating protein stability is the N-end rule, which links the identity of the N-terminal residue of a protein to its half-life. According to the N-end rule, a protein is stable if the exposed N-terminal residue is a small amino acid and unstable if it is large and bulky (Varshavsky, 2011). To establish whether the difference in half-life between proteins with long or short N-terminal disorder could be explained by differences in degradation dynamics due to the N-end rule, we compared the frequencies of amino acids in the second N-terminal residue (after removal of the initiator methionine by methionine aminopeptidases) in proteins with long or short disordered N-termini in yeast, mouse and human (**Figure S1H**). We also calculated the frequency of each amino acid in the second N-terminal residue for the entire proteomes.

In all analyzed organisms, the distributions of destabilizing amino acid frequencies (primary, secondary, or tertiary destabilizing, according to the N-end rule, see **Figure S1H**) are not significantly different between proteins with long N-terminal disordered segments and the entire proteomes ($P = 0.2$ in yeast, $P = 0.5$ in mouse, $P = 0.1$ in human, chi-squared test). The same is true in yeast and mouse for the distributions of destabilizing amino acid frequencies between the groups of proteins with long or short N-terminal disorder ($P = 0.1$ in yeast, $P = 0.2$ in mouse, chi-squared test). In human, the distributions of destabilizing amino acid frequencies are different between proteins with long or short N-terminal disorder ($P = 1 \times 10^{-2}$). It is not the case, however, that all types of destabilizing N-terminal amino acids are more common in human proteins with long N-terminal disordered regions, and thus account for their shorter half-life. In fact, several destabilizing residue types are more common in proteins with short N-terminal disorder (**Figure S1H**). These results indicate that the N-end rule does not account for the global differences in half-life among proteins with long or short N-terminal disorder.

It should be noted that this analysis makes the simplifying assumption that the initiator methionine is removed from all expressed proteins. The action of the N-terminal methionine amino-peptidase pathway, or the activity of proteases such as signal peptidases and caspases, removes the first methionine of most proteins or cleaves an internal recognition site

and exposes the amino-acid next to the methionine or any internal residue next to a cleavage site, respectively (Meinnel et al., 2006). Although a large fraction of the proteins in most proteomes are estimated to lose their N-terminal methionine, it is not clear which proteins are trimmed by exopeptidases after translation *in vivo*. Moreover, it is often unclear to what extent the N-terminus is trimmed. Recently, Lange and Overall assembled a database that includes results from several "terminomics" studies in which *in vivo* information about the actual protein N- and C- termini is collected (Lange and Overall, 2011). We used this database to find proteins for which there is experimental evidence for the removal of the first methionine residue. We then analyzed the distribution of amino acids at the N-terminus for these proteins with proven records for *in vivo* trimming. As before, we compared the groups of proteins with long or short N-terminal disordered segments for which protein half-life is available. Not enough proteins have proven records for *in vivo* methionine removal, have long N-terminal disordered segments, and have half-life information in yeast and human to get reliable results. However, amino acid frequency distributions could be calculated for mouse and statistical analysis revealed no significant difference between the frequencies of N-terminal amino acids after trimming between groups of proteins with long or short N-terminal disordered regions ($P > 0.1$, Wilcoxon signed-rank test). These results, combined with our analyses above (**Figure S1H**), indicate that the N-end rule is unlikely to account for the global differences in half-life of proteins with long or short N-terminal disorder.

SUPPLEMENTAL DISCUSSION

Discussion S1. Proteins without disordered segments can still be degraded quickly
Not only can proteins with long disordered segments still have a long half-life, proteins without disordered segments can still be degraded quickly (**Table 1C**). Several factors may target a protein more efficiently to the proteasome and thus shorten its half-life. For example, post-translational modifications such as phosphorylation, methylation, N-acetylation, and ubiquitination itself may destabilize or unfold protein regions (Hagai et al., 2011; Hagai and Levy, 2010; Hwang et al., 2010; Kim et al., 2014; Lee et al., 2012) or direct the activity of accessory factors that unfold substrates and present them to the proteasome as discussed in the main text for the p97/VCP ATPase (Beskow et al., 2009). Other factors that contribute to shorter half-life of individual or groups of proteins are the availability of ubiquitinating enzymes and sequence determinants (e.g. KEN box and destruction box motifs, N-end rule, PEST sequences)(Bachmair et al., 1986; Pfleger and Kirschner, 2000; Rogers et al., 1986). Nevertheless our observations suggest that, upon recruitment of a substrate to the proteasome, terminal or internal disordered segments influence protein half-life as an underlying factor and that this can be modulated by other cellular mechanisms, sequence determinants and the structural stability of the substrate.

Discussion S2. Disordered segments could influence the dynamics and regulation of signaling pathways (extended)
Internal disordered segments in proteins can be cleaved by the proteasome to generate functionally active partial fragments that have crucial regulatory functions as demonstrated for the transcription factors NF-kB and Ci (Chen et al., 1999; Palombella et al., 1994; Piwko and Jentsch, 2006; Tian et al., 2005). If the protein functions in a homo- or a heteromeric complex, then the cleaved fragments can have important regulatory properties such as inducing a switch-like behavior by sequestering full-length proteins and acting in a dominant negative manner (Buchler and Louis, 2008). Furthermore, since the presence of a subunit containing disordered segments can target an entire protein complex for degradation (Prakash et al., 2009), variation in disordered segments of individual subunits may also influence the half-life of their interaction partners and that of the homo- or hetero-oligomeric complexes involving such proteins (Lin et al., 2000), thereby regulating the abundance of entire protein complexes.

Disordered segments could also be exploited to design signaling or transcription circuits composed of proteins with defined turnover rates, thereby generating networks with desired properties. For example, engineered kinases or transcription factors with a long terminal disordered segment may turn over rapidly and hence contribute to an ultra-sensitive or all-or-none response (Alon, 2007; Kiel et al., 2010).

SUPPLEMENTAL EXPERIMENTAL PROCEDURES

Yeast protein half-life data

Data on *in vivo* protein half-life for *Saccharomyces cerevisiae* was obtained from Belle *et al.* (Belle et al., 2006), who measured protein turnover by Western blot analysis of TAP-tagged genes as a function of time following the inhibition of protein synthesis. We discarded 366 proteins with a half-life of exactly 300 minutes, as the original paper assigned this value to stable proteins for which degradation curves could not be fitted by an exponential decay function and thus half-life could not be determined. Moreover, to make sure that clear outliers would not affect the statistics, we removed seven proteins with extremely long half-lives of >6000 minutes from the data. Removal of these 373 highly stable proteins with long half-lives does not bias the results of our analyses (see **Supplemental Results S6**).

Half-life groups

The yeast proteome was classified into three groups of roughly equal size based on half-life: (i) short-lived proteins (half-life ≤30 minutes), (ii) medium half-life proteins (half-life 31-70 minutes), and (iii) long-lived proteins (half-life >70 minutes). We analyzed the length of N- and C-terminal disorder within each group (**Figures 1E** and **S1E**). In order to assess the robustness of our results, we performed control calculations with variations on the half-life cutoffs: we grouped the proteome into two half-life groups, based on the median (≤42 minutes, short half-life; >42 minutes, long half-life; **Figure S1F**).

Disorder calculations and groups

All validated ORFs in the yeast genome were downloaded from UniProtKB/Swiss-Prot release 2010_11 (Uniprot-Consortium, 2011). Intrinsic disorder was predicted for all protein sequences using the DISOPRED2 (Ward et al., 2004) software, with default settings. DISOPRED2 is a support vector machine-based classifier, trained on missing electron density in solved crystal structures, which performs well in CASP assessments (Bordoli et al., 2007; Noivirt-Brik et al., 2009). Based on the predicted disorder, we calculated several properties relating to the length and location of disordered segments in the protein sequences. We then used this information to divide the yeast proteome into different groups:

(a) The length of the disordered segment at the protein N- and C-terminus

We counted the number of residues predicted to be disordered at the protein termini, treating the N- and C-termini separately. For this, we allowed for minor (up to three consecutive residues) stretches of structured residues. This means that we considered a disordered region ended when encountering a stretch of minimally four structured residues. Thus, continuous stretches of three or less structured residues were regarded as belonging to the disordered terminus and included in the calculation of the length of the disordered terminus (except when they were the start of stretches of four or more structured residues that would end the disordered region). Depending on the length of the disordered terminus, we classified the proteins into two groups: (i) those that have short (≤30 residues) and (ii) those that have long (>30 residues) disordered termini (**Figure 1**). We based the cutoff for long and short disordered termini on recent molecular models of the proteasome (da Fonseca et al., 2012; Lander et al., 2012; Lasker et al., 2012; Matyskiela et al., 2013; Sledz et al., 2013), and *in vitro* experimental studies using purified proteasomes showing that there is a critical minimum length of about 30 residues that allows a disordered protein terminus to efficiently initiate degradation (Inobe et al., 2011). In order to assess the robustness of our results, we performed control calculations with variations on the length cutoff (**Table S1E**).

(b) The presence of an internal disordered segment

The proteolytic sites are buried deep within the proteasome core particle, accessible only through a long narrow channel, and the same is true for the ATPase motor that drives protein substrates through the degradation channel (da Fonseca et al., 2012; Lander et al., 2012; Lasker et al., 2012; Matyskiela et al., 2013; Sledz et al., 2013). To investigate if the presence of internal disordered regions in a protein influences its half-life, we identified internal disordered segments as continuous stretches of at least 40 disordered amino acids in the middle of a protein (see main text). As for terminal disordered regions, we allowed for minor stretches of up to three structured residues in-between the disordered residues (see above). We discarded any N- and C-terminal disordered segments as defined above from the calculation of internal disordered segments. According to these definitions, we grouped the yeast proteome into two groups: (i) proteins that contained internal stretches of intrinsic disorder of at least 40 residues, and (ii) proteins that did not (**Figure 2**). In order to assess the robustness of our results, we performed control calculations with variations on the length cutoff (**Table S1E**). Systematically varying the length cutoff for identifying an internal intrinsically disordered segment revealed that maximal difference in median half-life and statistical significance was obtained for a value of 40 amino acids. We also investigated the half-lives of proteins with multiple internal disordered regions (**Figures 2C** and **S2C**).

(c) The overall degree of disorder

The fraction of disordered residues (i.e. overall degree of disorder), which is an estimate of the packing, folding and structural stability of a protein, by itself correlates with protein half-life, although previous studies disagree on the extent of the effect (Gsponer et al., 2008; Tompa et al., 2008; Yen et al., 2008). Proteins with a greater overall disorder degree contain longer terminal and internal disordered segments (**Figure S3A**). To determine whether the effects of continuous

stretches of disordered residues (i.e. disordered segments) on protein turnover (**Figures 1** and **2**) are independent of the overall degree of disorder, we calculated disorder degree as the fraction of residues in the protein that was predicted to be disordered (number of disordered residues divided by sequence length). We then matched proteins that have a similar fraction of disordered residues but have varying combinations of disordered segments (long or short N-terminal disorder and/or presence or absence of internal disordered segments). The results are shown in **Figure S3** and are reported in the main text (section "The effects of disordered segments on protein half-life are independent of the overall disorder degree"), and in an extended section in **Supplemental Results S1**. We also made classifications based on the scores reported by the disorder predictors, both for the average score of the entire protein (**Figures S1C** and **S2A**) and of the disordered regions alone (**Figures S1D** and **S2B**).

Unless otherwise noted, the disorder predictor used in our analyses is DISOPRED2. To ensure that our results are independent of the method used for intrinsic disorder prediction, we repeated the same calculations using disorder information from the IUPred (Dosztanyi et al., 2005) and PONDR VSL1 (Obradovic et al., 2005) predictors (**Figures S1C-D** and **S2A-C**, **Tables S1B-D**). IUPred was run in 'long' disorder prediction mode, and the disorder thresholds were adjusted to a score of 0.4 for IUPred and 0.6 for PONDR. These thresholds were chosen to maximize agreement with the average level of disorder observed in the DISPROT database release 5.7 (Sickmeier et al., 2007). We chose to complement our DISOPRED2 calculations with IUPred and PONDR because the three methods are very different, and are therefore likely to provide good control data. DISOPRED2 is a support vector machine-based classifier, trained on missing electron density in solved crystal structures, while IUPred is a sequence-based method that estimates inter-residue interactions. Sequences with less favorable predicted pairwise interaction energies are more likely to be disordered, due to a lack of stabilizing contacts. The third predictor, PONDR VSL1, employs logistic regression models of various sequence attributes, and is trained on missing electron density in crystal structures and disordered regions identified by other means. Consistently, we get very similar results with the three different disorder predictors (**Figures S1C-D** and **S2A-C**, **Tables S1B-D**).

Data integration and description

Integration of the data on yeast protein half-life, and various other datasets (**Table S1A**), with the length and location of intrinsic disorder within the protein sequences resulted in a dataset of 3273 proteins. This covers about two-thirds of the complete yeast proteome (Christie et al., 2004). The data for our DISOPRED2-based analyses is available in **Table S2**.

The distribution of half-life values is approximately log-normal (**Figure S1A**) with an enrichment for proteins with a very fast turnover rate of 2 and 3 minutes, as was also noted by Belle *et al.* (Belle et al., 2006). The half-lives have a mean of 98 minutes and a median of 43 minutes. N- and C-terminal disorder length distributions are similar to each other (**Figure S1A**): there are a large number of proteins with very short terminal disorder, and much fewer proteins with very long terminal disorder. The means for N- and C-terminal disorder length are 19 and 13 residues, respectively. The medians are 8 and 3 residues. In both datasets the frequency of encountering a terminal disordered segment of a certain length decreases rapidly with increasing length. Linear regression analysis of the two distributions plotted in log-log fashion showed a reasonable fit (N-terminal: $R^2 = 0.86$; C-terminal: $R^2 = 0.84$, data not shown), which could suggest a power law distribution (Stumpf and Porter, 2012). Larger lengths are observed for internal disordered segments (**Figure S1A**): the mean length of an internal disordered segment is 24 residues, while the median length is 14 residues. The shape of the distribution is similar to the terminal disorder types, and linear regression analysis of a log-log plot also shows a reasonable fit ($R^2 = 0.87$, data not shown).

Statistical methods and estimation of significance

All analyses and statistics employed in our study were selected to be highly robust. As discussed above, most datasets are not normally distributed (**Figure S1A**). Therefore we exclusively employed statistical tests that do not assume the data to come from a specific type of probability distribution and do not infer parameters of such distributions (such as the mean and variance for Gaussian distributions), i.e. we exclusively employed non-parametric statistics for estimating statistical significant (Mann-Whitney *U*, Wilcoxon Signed-Rank, Kruskal-Wallis, and Kolmogorov-Smirnov tests). Although parametric statistics generally have more power, which means that they have a smaller chance to commit a type II error (failure to reject a false null hypothesis), they commonly assume normally distributed data and violations of these and other assumptions can lead to misleading results. In contrast, non-parametric statistics rely of few assumptions about the data and, given that our data is not normally distributed, these are more robust than parametric statistics.

We primarily use the Mann-Whitney *U* test to compare half-life distributions of different groups of proteins and to estimate significance (but reach the same conclusions using the Kolmogorov-Smirnov test, **Table S1K**). The Mann-Whitney *U* test, also known as the Wilcoxon Rank-Sum test, evaluates whether two samples are likely to come from the same underlying distribution (H_0), and can be used to assess whether the medians of two distributions are significantly different. The Mann-Whitney *U* test assumes that the compared distributions have similar shapes, which is fair for our data, because the groups that we compare are always subsets of the whole data and have very similar overall shapes as demonstrated by the various boxplots throughout the paper. Boxplots are non-parametric visualizations for gaining insights into various properties of the distribution, such as the median, interquartile range, minimum and maximum, outliers, and skewness. The Kolmogorov-Smirnov test also evaluates whether two samples of observations come from

the same distribution or not, and does so by determining the maximum vertical deviation between the empirical distribution functions of the two samples. The Kruskal-Wallis test extends the Mann-Whitney *U* test to three or more sample groups. When comparing half-life distributions of proteins paired by overall disorder degree, *P* values were calculated using the Wilcoxon Signed-Rank test, which is a non-parametric test for assessing difference between two paired samples.

Throughout our analyses, we report medians and interquartile ranges, which are robust measures of central tendency and dispersion, rather than means and standard deviations, which are sensitive to outliers and less resistant to errors produced by deviations from assumptions. Furthermore, we not only report *P* values of statistical differences between distributions, but also show that the magnitudes of the differences (effect sizes, which we report as the difference between the medians of the compared distributions) are of a biologically relevant order of magnitude.

Our primary analyses rely on binning and cutoffs for the data describing structural disorder, rather than for example on linear regression and correlation analyses, because this approach best captures the biology of protein degradation influenced by disordered segments: half-life does not depend linearly on the length of the disordered segment, which becomes only relevant from a minimal value (see main text and **Figure S1B**). Thus, we do not presume the existence of a linear relationship, but simply investigate whether the group of proteins with disordered segments has a different half-life when compared to the group without disordered segments by binning proteins into groups based on the critical cutoff found in biochemical studies. Grouping of data points into classes and assessing the difference between the distributions is a powerful way to identify the existence of a relationship without assuming any underlying model of correlation.

Even if one assumes a linear relationship, calculation of the best linear fit for a large number of experimentally determined data points is unlikely to yield high correlation values. Indeed, although the length of disordered segments at various positions in proteins negatively correlates with half-life, the *r* values are of a very small magnitude (Pearson r = -0.02 for the correlation between the length of N-terminal disorder and protein half-life, r = -0.01 for C-terminal disorder, and r = -0.05 for the longest internal disordered segment, **Figure S1B**). Only the correlation between internal disorder length and protein half-life is statistically significant ($P = 3 \times 10^{-3}$). These results agree with our reported observations suggesting an inverse relationship between the presence of disordered segments and protein half-life, but, more importantly, underscore that correlation analyses and assumptions about a linear relationship are insufficient to capture the biology of protein degradation influenced by disordered segments.

Plots of the data and all statistical tests to estimate significance were carried out using the R statistical package (R Development Core Team).

Paralog calculations

A complete list of paralogous proteins in yeast was obtained in two steps:

1. First, we ran the program BLASTClust (Altschul et al., 1990) on the *S. cerevisiae* proteome. BLASTClust works by performing pairwise sequence comparisons among all yeast proteins and subsequently grouping the proteins by single-linkage clustering. It accepts various parameters affecting the stringency of clustering – in this study, sequences were registered as a pairwise match when they are at least 25% identical (parameter S) over an area covering 60% of the length (parameter L). This ensured, on the one hand, that pairs are sufficiently similar to reduce the number of false-positive paralog pairs and, on the other hand, that the genes could have diverged enough to allow for their half-lives and/or disordered regions to change. The heuristic list of paralogs was obtained by forming all possible pairs within each cluster.
2. In order to include more divergent paralogs that were not picked up by the procedure in step 1, we added to the list all known paralog pairs that resulted from the whole genome duplication in yeast (Wolfe and Shields, 1997). This additional data was obtained from the Yeast Gene Order Browser (http://wolfe.gen.tcd.ie/ygob/) Version 3 (Gordon et al., 2009).

Table S7 shows all 1440 pairs for which protein half-life data is available for both paralogs. To calculate the differences in half-life ΔH and N-terminal disorder length ΔL between the individual proteins in a paralog pair, the following convention is made: ΔL is defined to be always positive (**Figure 4**). In other words, we define "paralog 1" to be the paralog with the longer N-terminal disorder and "paralog 2" the protein with the shorter N-terminal disorder of the paralog pair. ΔL is then obtained by subtracting the N-terminal disorder length of paralog 2 from the N-terminal disorder length of paralog 1 ($\Delta L = L_1 - L_2$, with $L_1 \geq L_2$). To calculate ΔH, the order of paralogs in a pair is maintained, so that ΔH can be positive or negative ($\Delta H = H_1 - H_2$). Thus, ΔH is negative whenever the relationship "longer disordered N-terminus = shorter half-life" holds true. We separated the paralog pairs into two groups according to the difference in the length of their N-terminal disordered segments: pairs where one paralog has a short and the other paralog a long disordered N-terminus, and pairs where both paralogs have short or both have long disordered N-termini. Similarly, for internal disorder, we define the difference in the number of internal disordered regions (ΔI) to be always positive ($\Delta I = I_1 - I_2$, with $I_1 \geq I_2$). We separated the paralog pairs into two categories: pairs with an identical number of sites ($\Delta I = 0$), and pairs where one paralog has one or more sites more than the other ($\Delta I \geq 1$). Since the $\Delta I = 0$ pairs cannot be arranged based on the number of internal disordered segments (i.e. $I_1 = I_2$ and thus $I_1 > I_2$ is never true), we ordered the members of

such pairs by the total number of residues that make up all internal disordered segments (internal disorder length, IL) in these proteins (analogous to N-terminal disorder length ordering used above). We did this twice to simulate two different evolutionary scenarios: once we subtracted the half-life of the paralog with the longest total internal disorder from the half-life of the paralog with the shorter total internal disorder (length of internal disorder increased during evolution), and once the other way around (length of internal disorder decreased during evolution). Thus, for each $\Delta I = 0$ pair, we once calculate ΔH as $H_1 - H_2$ where $IL_1 \geq IL_2$, and once as $H_1 - H_2$ where $IL_1 \leq IL_2$

We calculated the half-life and N-terminal disorder length differences for several randomized controls to ensure that the trend observed in the paralog pair analysis is not merely a product of chance. Specifically, we randomized (i) the disordered N-terminus length, (ii) the protein half-life and (iii) both values among all proteins (**Table S6B**). Thus, the overall distribution of values remains intact during the randomization, while the individual assignment to paralog pair groups may change. To make sure that the observed effect is paralog pair specific we also generated a random set of protein pairs from the singletons (clusters of size 1, corresponding to proteins that lack a paralog) in the BLASTClust analysis (**Table S6B**).

Mouse and human data

To assess the effects of terminal and internal disordered segments on protein half-life in other organisms than yeast, we performed the same analyses with mouse protein half-life data and human relative degradation rates, with the exception of the paralogs analysis (see section "The experimental design used to measure protein half-life in mouse and human does not permit a confident investigation of half-life differences among paralogous proteins", below). Reviewed protein sequences for mouse and human were downloaded from UniProtKB/Swiss-Prot release 2011_4 (Uniprot-Consortium, 2011). Data on half-life for ~4500 proteins in NIH3T3 mouse fibroblasts was obtained from Schwanhäusser *et al.* (Schwanhausser et al., 2011). Data on relative degradation rates for ~4000 proteins in human THP-1 myelomonocytic leukemia cells, under conditions that stimulate proliferation, was obtained from Kristensen *et al.* (Kristensen et al., 2013). Both studies make use of stable isotope labeling by amino acids in cell culture (SILAC) in combination with mass spectrometry. Upon transferring cells from light to heavy medium, newly synthesized proteins incorporate heavy labeled amino acids, while the pre-existing proteins remain in the light from. Protein half-lives and degradation rates were derived from the ratio between the heavy and light peptides, measured using mass spectrometry at different time points after the transfer of cells to heavy medium.

The experimental design used to measure protein half-life in mouse and human does not permit a confident investigation of half-life differences among paralogous proteins

Protein turnover in mouse (Schwanhausser et al., 2011) and human (Kristensen et al., 2013) has been measured using stable isotope labeling by amino acids in cell culture (SILAC) in combination with mass spectrometry. Upon transferring cells from light to heavy medium, newly synthesized proteins incorporate heavy labeled amino acids, while the pre-existing proteins remain in the light from. Protein half-lives and degradation rates were derived from the ratio between the heavy and light peptides, measured using mass spectrometry at different time points after the transfer of cells to heavy medium.

Peptides that are detected using mass spectrometry need to be assigned to proteins. In most cases, unique peptides can be mapped to proteins with reasonable confidence. However, some proteins give rise to identical peptides, in which case it becomes hard to determine which protein the peptides originated from (Li et al., 2009; Nesvizhskii and Aebersold, 2005). Most methods for assigning MS peptides to proteins require that multiple, distinct peptides are present in order for a protein to be identified. Nevertheless, when proteins are very similar, problems arise, as they may not give rise to enough unique peptides to reliably quantify similar proteins. Since paralogous proteins are similar to each other by definition, and have in some cases hardly diverged during evolution, such sequences can be hard or impossible to differentiate using mass spectrometry. Similarly, proteins arising from alternative splicing or alternative initiation during transcription or translation are difficult to characterize. Thus, the current limitation of mass spectrometry to reliably distinguish peptides arising from proteins with similar sequences restricts us from performing an analysis of half-life differences among paralogous proteins in mouse and human.

Protein structures

The model of the 26S proteasome (**Figure 5**) is based on the electron microscopy density map from (Lander et al., 2012) (EMDataBank ID: 1992). The figure was prepared using the UCSF Chimera package (Pettersen et al., 2004). Protein structure figures of Cytochrome C oxidase (PDB ID: 1U96 (Abajian et al., 2004)) and Triosephosphate isomerase (PDB ID: 1YPI (Lolis et al., 1990)) (**Figure 1A**) were prepared using YASARA (Krieger et al., 2002). Distance measurements on the crystal structure of the 20S yeast proteasome (PDB ID: 1RYP (Groll et al., 1997)) were done using the PyMOL Molecular Graphics System, Version 1.3 (Schrodinger, 2010) (**Figure S2D**).

SUPPLEMENTAL REFERENCES

Abajian, C., Yatsunyk, L.A., Ramirez, B.E., and Rosenzweig, A.C. (2004). Yeast cox17 solution structure and Copper(I) binding. J. Biol. Chem. *279*, 53584-53592.

Alon, U. (2007). Network motifs: theory and experimental approaches. Nat. Rev. Genet. *8*, 450-461.

Altschul, S.F., Gish, W., Miller, W., Myers, E.W., and Lipman, D.J. (1990). Basic local alignment search tool. J. Mol. Biol. *215*, 403-410.

Bachmair, A., Finley, D., and Varshavsky, A. (1986). In vivo half-life of a protein is a function of its amino-terminal residue. Science *234*, 179-186.

Belle, A., Tanay, A., Bitincka, L., Shamir, R., and O'Shea, E.K. (2006). Quantification of protein half-lives in the budding yeast proteome. Proc. Natl. Acad. Sci. USA *103*, 13004-13009.

Beskow, A., Grimberg, K.B., Bott, L.C., Salomons, F.A., Dantuma, N.P., and Young, P. (2009). A conserved unfoldase activity for the p97 AAA-ATPase in proteasomal degradation. J. Mol. Biol. *394*, 732-746.

Bordoli, L., Kiefer, F., and Schwede, T. (2007). Assessment of disorder predictions in CASP7. Proteins *69 Suppl 8*, 129-136.

Buchler, N.E., and Louis, M. (2008). Molecular titration and ultrasensitivity in regulatory networks. J. Mol. Biol. *384*, 1106-1119.

Chen, C.H., von Kessler, D.P., Park, W., Wang, B., Ma, Y., and Beachy, P.A. (1999). Nuclear trafficking of Cubitus interruptus in the transcriptional regulation of Hedgehog target gene expression. Cell *98*, 305-316.

Christie, K.R., Weng, S., Balakrishnan, R., Costanzo, M.C., Dolinski, K., Dwight, S.S., Engel, S.R., Feierbach, B., Fisk, D.G., Hirschman, J.E.*, et al.* (2004). Saccharomyces Genome Database (SGD) provides tools to identify and analyze sequences from Saccharomyces cerevisiae and related sequences from other organisms. Nucleic Acids Res. *32*, D311-314.

da Fonseca, P.C., He, J., and Morris, E.P. (2012). Molecular model of the human 26S proteasome. Mol. Cell *46*, 54-66.

Davey, N.E., Haslam, N.J., Shields, D.C., and Edwards, R.J. (2010). SLiMFinder: a web server to find novel, significantly over-represented, short protein motifs. Nucleic Acids Res. *38*, W534-539.

Davey, N.E., Van Roey, K., Weatheritt, R.J., Toedt, G., Uyar, B., Altenberg, B., Budd, A., Diella, F., Dinkel, H., and Gibson, T.J. (2012). Attributes of short linear motifs. Mol. Biosyst. *8*, 268-281.

Dice, J.F., Dehlinger, P.J., and Schimke, R.T. (1973). Studies on the correlation between size and relative degradation rate of soluble proteins. J. Biol. Chem. *248*, 4220-4228.

Dinkel, H., Michael, S., Weatheritt, R.J., Davey, N.E., Van Roey, K., Altenberg, B., Toedt, G., Uyar, B., Seiler, M., Budd, A.*, et al.* (2012). ELM--the database of eukaryotic linear motifs. Nucleic Acids Res. *40*, D242-251.

Dosztanyi, Z., Csizmok, V., Tompa, P., and Simon, I. (2005). The pairwise energy content estimated from amino acid composition discriminates between folded and intrinsically unstructured proteins. J. Mol. Biol. *347*, 827-839.

Gordon, J.L., Byrne, K.P., and Wolfe, K.H. (2009). Additions, losses, and rearrangements on the evolutionary route from a reconstructed ancestor to the modern Saccharomyces cerevisiae genome. PLoS Genet. *5*, e1000485.

Groll, M., Ditzel, L., Lowe, J., Stock, D., Bochtler, M., Bartunik, H.D., and Huber, R. (1997). Structure of 20S proteasome from yeast at 2.4 A resolution. Nature *386*, 463-471.

Gsponer, J., Futschik, M.E., Teichmann, S.A., and Babu, M.M. (2008). Tight regulation of unstructured proteins: from transcript synthesis to protein degradation. Science *322*, 1365-1368.

Hagai, T., Azia, A., Toth-Petroczy, A., and Levy, Y. (2011). Intrinsic disorder in ubiquitination substrates. J. Mol. Biol. *412*, 319-324.

Hagai, T., and Levy, Y. (2010). Ubiquitin not only serves as a tag but also assists degradation by inducing protein unfolding. Proc. Natl. Acad. Sci. USA *107*, 2001-2006.

Huang da, W., Sherman, B.T., and Lempicki, R.A. (2009a). Bioinformatics enrichment tools: paths toward the comprehensive functional analysis of large gene lists. Nucleic Acids Res. *37*, 1-13.

Huang da, W., Sherman, B.T., and Lempicki, R.A. (2009b). Systematic and integrative analysis of large gene lists using DAVID bioinformatics resources. Nat. Protoc. *4*, 44-57.

Hwang, C.S., Shemorry, A., and Varshavsky, A. (2010). N-terminal acetylation of cellular proteins creates specific degradation signals. Science *327*, 973-977.

Inobe, T., Fishbain, S., Prakash, S., and Matouschek, A. (2011). Defining the geometry of the two-component proteasome degron. Nat. Chem. Biol. *7*, 161-167.

Jorda, J., Xue, B., Uversky, V.N., and Kajava, A.V. (2010). Protein tandem repeats - the more perfect, the less structured. FEBS J. *277*, 2673-2682.

Kiel, C., Yus, E., and Serrano, L. (2010). Engineering signal transduction pathways. Cell *140*, 33-47.

Kim, H., Melen, K., Osterberg, M., and von Heijne, G. (2006). A global topology map of the Saccharomyces cerevisiae membrane proteome. Proc. Natl. Acad. Sci. USA *103*, 11142-11147.

Kim, H.K., Kim, R.R., Oh, J.H., Cho, H., Varshavsky, A., and Hwang, C.S. (2014). The N-terminal methionine of cellular proteins as a degradation signal. Cell *156*, 158-169.

Krieger, E., Koraimann, G., and Vriend, G. (2002). Increasing the precision of comparative models with YASARA NOVA--a self-parameterizing force field. Proteins *47*, 393-402.

Kristensen, A.R., Gsponer, J., and Foster, L.J. (2013). Protein synthesis rate is the predominant regulator of protein expression during differentiation. Mol. Syst. Biol. *9*, 689.

Lander, G.C., Estrin, E., Matyskiela, M.E., Bashore, C., Nogales, E., and Martin, A. (2012). Complete subunit architecture of the proteasome regulatory particle. Nature *482*, 186-191.

Lange, P.F., and Overall, C.M. (2011). TopFIND, a knowledgebase linking protein termini with function. Nat. Methods *8*, 703-704.

Lasker, K., Forster, F., Bohn, S., Walzthoeni, T., Villa, E., Unverdorben, P., Beck, F., Aebersold, R., Sali, A., and Baumeister, W. (2012). Molecular architecture of the 26S proteasome holocomplex determined by an integrative approach. Proc. Natl. Acad. Sci. USA *109*, 1380-1387.

Lee, J.M., Lee, J.S., Kim, H., Kim, K., Park, H., Kim, J.Y., Lee, S.H., Kim, I.S., Kim, J., Lee, M., *et al.* (2012). EZH2 generates a methyl degron that is recognized by the DCAF1/DDB1/CUL4 E3 ubiquitin ligase complex. Mol. Cell *48*, 572-586.

Li, Y.F., Arnold, R.J., Li, Y., Radivojac, P., Sheng, Q., and Tang, H. (2009). A bayesian approach to protein inference problem in shotgun proteomics. J. Comput. Biol. *16*, 1183-1193.

Lin, L., DeMartino, G.N., and Greene, W.C. (2000). Cotranslational dimerization of the Rel homology domain of NF-kappaB1 generates p50-p105 heterodimers and is required for effective p50 production. EMBO J. *19*, 4712-4722.

Liu, Z., Yuan, F., Ren, J., Cao, J., Zhou, Y., Yang, Q., and Xue, Y. (2012). GPS-ARM: computational analysis of the APC/C recognition motif by predicting D-boxes and KEN-boxes. PLoS One *7*, e34370.

Lolis, E., Alber, T., Davenport, R.C., Rose, D., Hartman, F.C., and Petsko, G.A. (1990). Structure of yeast triosephosphate isomerase at 1.9-A resolution. Biochemistry *29*, 6609-6618.

Matyskiela, M.E., Lander, G.C., and Martin, A. (2013). Conformational switching of the 26S proteasome enables substrate degradation. Nat. Struct. Mol. Biol. *20*, 781-788.

Meinnel, T., Serero, A., and Giglione, C. (2006). Impact of the N-terminal amino acid on targeted protein degradation.

Biol. Chem. *387*, 839-851.

Melen, K., Krogh, A., and von Heijne, G. (2003). Reliability measures for membrane protein topology prediction algorithms. J. Mol. Biol. *327*, 735-744.

Meusser, B., Hirsch, C., Jarosch, E., and Sommer, T. (2005). ERAD: the long road to destruction. Nat. Cell Biol. *7*, 766-772.

Neduva, V., Linding, R., Su-Angrand, I., Stark, A., de Masi, F., Gibson, T.J., Lewis, J., Serrano, L., and Russell, R.B. (2005). Systematic discovery of new recognition peptides mediating protein interaction networks. PLoS Biol. *3*, e405.

Nesvizhskii, A.I., and Aebersold, R. (2005). Interpretation of shotgun proteomic data: the protein inference problem. Mol. Cell. Proteomics *4*, 1419-1440.

Newman, J.R., Ghaemmaghami, S., Ihmels, J., Breslow, D.K., Noble, M., DeRisi, J.L., and Weissman, J.S. (2006). Single-cell proteomic analysis of S. cerevisiae reveals the architecture of biological noise. Nature *441*, 840-846.

Noivirt-Brik, O., Prilusky, J., and Sussman, J.L. (2009). Assessment of disorder predictions in CASP8. Proteins *77 Suppl 9*, 210-216.

Obradovic, Z., Peng, K., Vucetic, S., Radivojac, P., and Dunker, A.K. (2005). Exploiting heterogeneous sequence properties improves prediction of protein disorder. Proteins *61 Suppl 7*, 176-182.

Osterberg, M., Kim, H., Warringer, J., Melen, K., Blomberg, A., and von Heijne, G. (2006). Phenotypic effects of membrane protein overexpression in Saccharomyces cerevisiae. Proc. Natl. Acad. Sci. USA *103*, 11148-11153.

Palombella, V.J., Rando, O.J., Goldberg, A.L., and Maniatis, T. (1994). The ubiquitin-proteasome pathway is required for processing the NF-kappa B1 precursor protein and the activation of NF-kappa B. Cell *78*, 773-785.

Pettersen, E.F., Goddard, T.D., Huang, C.C., Couch, G.S., Greenblatt, D.M., Meng, E.C., and Ferrin, T.E. (2004). UCSF Chimera--a visualization system for exploratory research and analysis. J. Comput. Chem. *25*, 1605-1612.

Pfleger, C.M., and Kirschner, M.W. (2000). The KEN box: an APC recognition signal distinct from the D box targeted by Cdh1. Genes Dev. *14*, 655-665.

Piper, R.C., and Katzmann, D.J. (2007). Biogenesis and function of multivesicular bodies. Annu. Rev. Cell. Dev. Biol. *23*, 519-547.

Piwko, W., and Jentsch, S. (2006). Proteasome-mediated protein processing by bidirectional degradation initiated from an internal site. Nat. Struct. Mol. Biol. *13*, 691-697.

Prakash, S., Inobe, T., Hatch, A.J., and Matouschek, A. (2009). Substrate selection by the proteasome during degradation of protein complexes. Nat. Chem. Biol. *5*, 29-36.

R Development Core Team. R: A language and environment for statistical computing (Vienna, Austria: R Foundation for Statistical Computing).

Raiborg, C., and Stenmark, H. (2009). The ESCRT machinery in endosomal sorting of ubiquitylated membrane proteins. Nature *458*, 445-452.

Rice, P., Longden, I., and Bleasby, A. (2000). EMBOSS: the European Molecular Biology Open Software Suite. Trends Genet. *16*, 276-277.

Rogers, S., Wells, R., and Rechsteiner, M. (1986). Amino acid sequences common to rapidly degraded proteins: the PEST hypothesis. Science *234*, 364-368.

Romero, P., Obradovic, Z., Li, X., Garner, E.C., Brown, C.J., and Dunker, A.K. (2001). Sequence complexity of disordered protein. Proteins *42*, 38-48.

Schrodinger, LLC (2010). The PyMOL Molecular Graphics System, Version 1.3r1.

Schwanhausser, B., Busse, D., Li, N., Dittmar, G., Schuchhardt, J., Wolf, J., Chen, W., and Selbach, M. (2011). Global

quantification of mammalian gene expression control. Nature *473*, 337-342.

Sickmeier, M., Hamilton, J.A., LeGall, T., Vacic, V., Cortese, M.S., Tantos, A., Szabo, B., Tompa, P., Chen, J., Uversky, V.N.*, et al.* (2007). DisProt: the Database of Disordered Proteins. Nucleic Acids Res. *35*, D786-793.

Simon, M., and Hancock, J.M. (2009). Tandem and cryptic amino acid repeats accumulate in disordered regions of proteins. Genome Biol. *10*, R59.

Sledz, P., Unverdorben, P., Beck, F., Pfeifer, G., Schweitzer, A., Forster, F., and Baumeister, W. (2013). Structure of the 26S proteasome with ATP-gammaS bound provides insights into the mechanism of nucleotide-dependent substrate translocation. Proc. Natl. Acad. Sci. USA *110*, 7264-7269.

Stumpf, M.P., and Porter, M.A. (2012). Mathematics. Critical truths about power laws. Science *335*, 665-666.

Tian, L., Holmgren, R.A., and Matouschek, A. (2005). A conserved processing mechanism regulates the activity of transcription factors Cubitus interruptus and NF-kappaB. Nat. Struct. Mol. Biol. *12*, 1045-1053.

Tompa, P., Prilusky, J., Silman, I., and Sussman, J.L. (2008). Structural disorder serves as a weak signal for intracellular protein degradation. Proteins *71*, 903-909.

Uniprot-Consortium (2011). Ongoing and future developments at the Universal Protein Resource. Nucleic Acids Res. *39*, D214-219.

Varshavsky, A. (2011). The N-end rule pathway and regulation by proteolysis. Protein Sci. *20*, 1298-1345.

Vembar, S.S., and Brodsky, J.L. (2008). One step at a time: endoplasmic reticulum-associated degradation. Nat. Rev. Mol. Cell Biol. *9*, 944-957.

Ward, J.J., Sodhi, J.S., McGuffin, L.J., Buxton, B.F., and Jones, D.T. (2004). Prediction and functional analysis of native disorder in proteins from the three kingdoms of life. J. Mol. Biol. *337*, 635-645.

Wolfe, K.H., and Shields, D.C. (1997). Molecular evidence for an ancient duplication of the entire yeast genome. Nature *387*, 708-713.

Yen, H.C., Xu, Q., Chou, D.M., Zhao, Z., and Elledge, S.J. (2008). Global protein stability profiling in mammalian cells. Science *322*, 918-923.
